# Metabolomic characterization and multi-target biological activities of *Saponaria pumila* and *Suaeda confusa*: integrating *in vitro* and *in silico* approaches

**DOI:** 10.1039/d6ra06442f

**Published:** 2026-09-29

**Authors:** Nilofar Nilofar, Emre Can Buluz, Savas Kaya, Jasmina Glamočlija, Neda Popović, Dejan Stojković, Josef Jeko, Cziaky Zoltan, Nikolaos Tzortzakis, Evren Yildiztugay, Gokhan Zengin

**Affiliations:** a Department of Pharmacy, University of Pisa Via Bonanno 6 56126 Pisa Italy; b Institute of Science, Department of Physics, Ege University İzmir 35100 Turkey; c Sivas Cumhuriyet University, Faculty of Science, Department of Chemistry 58140 Sivas Turkey; d Department of Plant Physiology, Institute for Biological Research “Siniša Stanković”-National Institute of Republic of Serbia, University of Belgrade Bulevar Despota Stefana 142 11108 Belgrade Serbia; e Science Park, University of Nyíregyháza Nyíregyháza Hungary; f Department of Agricultural Sciences, Biotechnology and Food Science, Cyprus University of Technology Limassol 3036 Cyprus; g Department of Biotechnology, Science Faculty, Selcuk University Konya Turkey; h Department of Biology, Science Faculty, Selcuk University Konya Turkey gokhanzengin@selcuk.edu.tr

## Abstract

In the present study, several extracts (ethyl acetate, methanol, and water) of *Saponaria pumila* and *Suaeda confusa* were used to characterize and identify their biological activities. The chemical characterization was performed using HPLC-ESI-Orbitrap-MS/MS. The total phenolic and flavonoid contents were determined by the Folin assay and AlCl_3_ assay, respectively. The antioxidant capacity of the extracts was determined by DPPH, ABTS, CUPRAC, FRAP, MCA, and PBD assays. The enzyme inhibitory activity of the tested extracts was examined against several enzymes, namely, cholinesterase (AChE and BChE), tyrosinase, and carbohydrate digestive enzymes (α-amylase and α-glucosidase). Among the tested extracts of both plants, the *S. pumila* MeOH extracts exhibited the highest total phenolic and flavonoid contents, measuring 44.82 mg GAE per g and 72.60 mg RE per g, respectively. The lowest values, 12.30 mg GAE per g and 8.33 mg RE per g, respectively, were recorded by the *S. confusa* water extract. The antioxidant capacity of the *S. pumila* extracts in radical scavenging and reducing power assays was in the order of methanol (MeOH) > water > ethyl acetate (EA) extract. In the case of the plant species *S. confusa*, the MeOH extracts again yielded the highest value, with the same pattern as in *S. pumila*. Water extracts comparatively showed the highest ABTS scavenging activity, with a value of 75.70 mg TE per g. The water extract of *S. pumila* exhibited the strongest inhibitory effect against AChE (2.00 mg GALAE per g) and tyrosinase (42.47 mg KAE per g), while its EA extract gave the highest BChE inhibitory activity (1.87 mg GALAE per g). The MeOH extracts of *S. confusa* exhibited the highest inhibition of AChE (2.59 mg GALAE per g) and tyrosinase (46.28 mg KAE per g). The EA extract of *S. pumila* showed no anti-AChE activity, while all extracts of *S. confusa* showed no activity against α-glucosidase. The antimicrobial activity of the extracts was screened, revealing species- and solvent-dependent activities. The highest susceptibility was observed against *Listeria monocytogenes* and *Aspergillus fumigatus* (MICs of 0.25–0.5 mg mL^−1^), while *Pseudomonas aeruginosa* showed notable resistance. The extracts of *S. pumila* and *S. confusa* possess strong antioxidant potential, along with promising neuroprotective and dermoprotective properties, warranting further investigation in future studies.

## Abstract 2

The tested plant species may serve as alternative raw materials for the development of functional applications.

## Graphical abstract

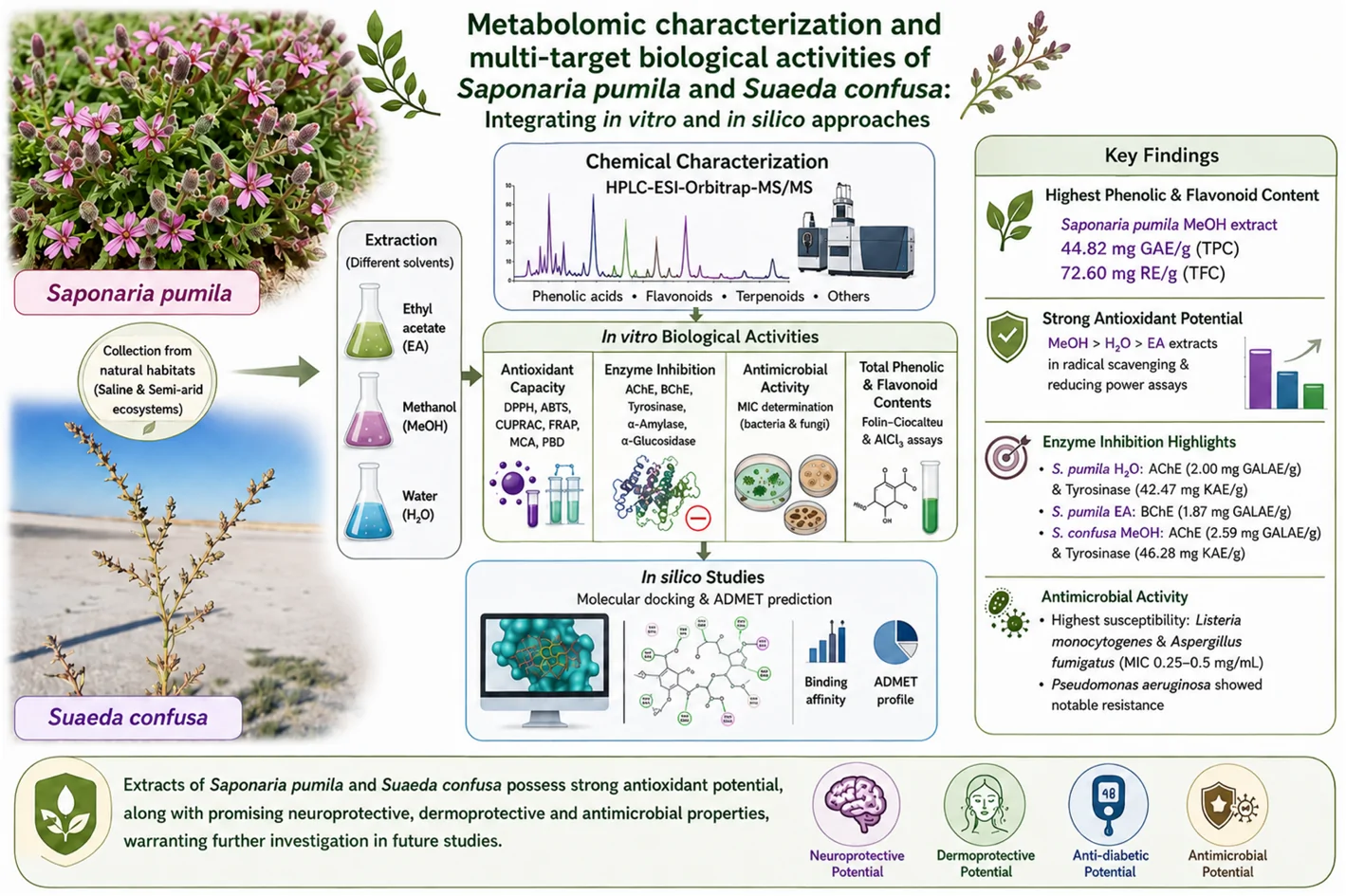

## Introduction

Plants have long been used for medicinal purposes, including lowering blood pressure and blood glucose, fighting viruses and fungi infections, easing diarrhea, and reducing inflammation; many also double as nutrient-dense “superfoods” thanks to their range of phytochemicals.^1–10^ That interest has now spread to cosmetics and pharmaceuticals, where plant extracts show up in dermo-cosmetic and cosmeceutical formulas.^10^ Modern chromatography and spectrometry make it convenient to analyze the bioactive compounds in these extracts. However, the chemical profiles and bioactivity of plant extracts are strongly influenced by factors such as the plant part used, cultivation and harvesting conditions, and the extraction process, including the choice of solvent.^11–14^ Solvent choice matters the most for antioxidant recovery—extraction yield and potency shift with the polarity of the solvent. Polar solvents, particularly aqueous blends of ethyl acetate, acetone, methanol, or ethanol, tend to pull the most polyphenols out of plant materials.^15^ Our previous research,^16^ along with other studies,^17–20^ has shown that methanol, ethanol, and ethyl acetate were successfully utilized to extract phenolic antioxidants from some plant sources like fruits, vegetables, grains, and peels. Therefore, selecting a suitable extraction solvent is critical for the efficient extraction of phytochemicals from plant materials, as the chemical nature and polarity of the solvent strongly influence both the yield and range of the bioactive compounds extracted.

Reactive oxygen species (ROS) are continuously generated in living systems as the by-products of normal cellular metabolism. Free radicals, with their unpaired electron, readily interact with biological macromolecules and induce oxidative damage. The uncontrolled activity of the ROS is inextricably linked with numerous health pathologies, including diabetes, cancer, neurological disorders, and premature aging.^21,22^ Plant secondary metabolites, particularly phenolic acids, flavonoids, and tannins, are among the most studied natural antioxidants in combating such harmful activities.^23^ Given these remarkable properties, plants represent valuable resources for developing novel therapeutic agents and natural health products.

Given this context, this study selected two plant species, *Saponaria pumila* and *Suaeda confusa*, due to the limited or lack of prior research on their bioactive compounds and biological activities. Plant species *Saponaria pumila* (Caryophyllaceae) is a perennial herb that forms cushions with compact, low-growth and large, pink flowers featuring a balloon-like calyx and protandrous flowering.^24^ Water extracts of *S. pumila* serve as active ingredients in skin-care products and are currently utilized in the cosmetics industry.^25^ Several studies reported the biologically active compounds in the members of the genus *Saponaria*. For example, in a previous study by Charalambous *et al.*,^26^ several saponins (saponarioside A, G octasaccharide, *etc.*) and phenolics (4,5-di-*O*-caffeoylquinic acid, rutin, *etc.*) were identified in *S. cypria*. Again, Smulek *et al.*^27^ identified several saponins (gypsogenin, hederagenin, hydroxyhederagenin and quillaic acid) in the methano extract of *S. officinalis*. In another study by Ozay *et al.*,^28^ the methanolic extract of *S. mesogitana* was rich in terms of caffeic and 2,5-dihydroxybenzoic acids.

Another plant species, *Suaeda confusa* (Chenopodiaceae), is now recognized as a synonym of *Suaeda acuminata* (C.A. Mey.) Moq., with taxonomic revisions replacing its earlier usage in floristic and phytosociological studies.^29^ In a previous study by Litvinenko *et al.*,^30^ the presence of apigenin, isorhamnetin, and cosmosiin was reported in the ethanolic extract of *S. acuminata*. In another study by Oueslati *et al.*,^31^ some phenolics including kaempferol, rosmarinic, gallic, and caffeic acids were found in several members of the genus *Suaeda*. The hydromethanolic extract of *S. fructicosa* was richer in terms of flavonoids such as luteolin and apigenin derivatives as reported by Zaier *et al.*^32^ In another study by Qasim *et al.*,^33^ the hydromethanolic extract of *S. fructicosa* contained a significant level of catechin.

Previously, no research has examined the influence of different commonly used solvents on the efficiency of extraction of phenolic components from *S. pumila* and *S. confusa*. To fill this gap in the literature, the current work investigated the influence of the solvents ethyl acetate (EA), methanol (MeOH), and water on the recovery of bioactive compounds from the plant species *S. pumila* and *S. confusa*. In addition to the phenolic and flavonoid determinations, the study analyzed the antioxidant capacity, enzyme inhibitory activities, and antibacterial activities of these plant extracts to identify the best solvent system for the efficient extraction and isolation of the bioactive constituents.

## Materials and methods

### Plant collection

Plant samples were collected in 2022 from Konya, Turkey (*S. confusa*: around Bolluk Lake, 950 m; *S. pumila*: from Dedem location to Egrigöl, 5 km, 1800 m). Botanical identification was officially carried out by Dr Evren Yildiztugay. Reference specimens bearing the code EY-3044 and EY-3112 were placed at the Selcuk University herbarium. The aerial components were separated immediately after collection and allowed to air-dry at room temperature in a well-ventilated, shady area. The plant material was pulverized into a fine powder once it had dried completely. The powdered samples were kept under constant conditions in light-proof containers to maintain chemical stability and prevent spoiling.

### Plant extract preparation

To separate bioactive chemicals, three solvents of varying polarities (distilled water, methanol, and ethyl acetate) were used. Ten grams of dried plant material were mixed with two hundred milliliters of each solvent. Depending on the type of solvent used, the extraction process was different. The plant material was macerated by soaking it in organic solvents for a whole day at room temperature. Instead, a 15 minutes hot-water infusion was used for water-based extraction. Following extraction, the organic solvent extracts were concentrated under reduced pressure using a rotary evaporator, and the aqueous extract was freeze-dried.

### Total phenolic and flavonoid contents

Total phenolic^34^ and total flavonoid^35^ contents in the extracts were determined using validated colorimetric methods, following established procedures. Calibration curves were prepared using gallic acid for phenolics and rutin for flavonoids, enabling the results to be expressed as GAE and RE per gram, respectively.

### Phytochemical analysis by HPLC-ESI-Orbitrap-MS/MS

The liquid chromatographic system was a Dionex 3000RS Ultimate system, where separations were achieved using a Thermo Accucore C18 (100 mm × 2.1 mm i.d., 2.6 µm) column themostated at 25 °C (±1 °C). A previously developed and successfully applied method was used.^36^ All analytical details are given in the supplementary materials.

### Antioxidant assays

A set of *in vitro* assays were employed to evaluate the antioxidant activity, following the protocol described by ref. 37. Reducing power was determined using the FRAP and CUPRAC methods, while the radical scavenging capacity was assessed by DPPH and ABTS assays. The outcomes of these four tests were converted into Trolox equivalents and reported as mg TE per gram of dried extract. In addition, the total antioxidant capacity was measured using a phosphomolybdenum (PBD) assay (mmol TE per g), and the metal chelating activity was expressed as mg EDTA equivalents per gram (mg EDTAE per g).

### Enzyme inhibitory activity assays

Following established colorimetric methods,^37^ the extracts were evaluated for their ability to inhibit five key enzymes: acetylcholinesterase (AChE), butyrylcholinesterase (BChE), tyrosinase, α-amylase, and α-glucosidase. To ensure comparability among samples, the inhibition results were expressed relative to appropriate reference compounds. Accordingly, cholinesterase inhibition was reported as mg galantamine equivalent (GALAE) per g, α-amylase and α-glucosidase inhibition as mmol acarbose equivalent (ACAE) per g, and tyrosinase inhibition as mg kojic acid equivalent (KAE) per g.

### Antimicrobial properties

Antibacterial and antifungal activities were determined using the microdilution method described previously.^38^ The microorganisms were obtained from the Department of Plant Physiology, Institute for Biological Research “Siniša Stanković,” University of Belgrade, National Institute of the Republic of Serbia. The Gram-positive bacteria *Staphylococcus aureus* (ATCC 6538), *Bacillus cereus* (clinical isolate), and *Listeria monocytogenes* (NCTC 7973), as well as Gram-negative bacteria *Pseudomonas aeruginosa* (ATCC 27853) and *Escherichia coli* (ATCC 35210), were used. A modified microdilution method was used to perform the antibacterial assay.^39^

The following micromycetes were used for the evaluation of antifungal activity: *Aspergillus fumigatus* (ATCC 1022), *Aspergillus niger* (ATCC 6275), *Trichoderma viride* (IAM 5061), *Penicillium funiculosum* (ATCC 36839) and *Penicillium verrucosum* var. *cyclopium* (food isolate). The antifungal activity was assessed using the modified microdilution method in 96-well microplates.^40^

The extracts' antimicrobial activity was determined in comparison to antibiotics streptomycin (Sigma Aldrich) or ampicillin (Sigma Aldrich) in sterile saline for antibacterial activity and fungicides bifonazole (Srbolek) and ketoconazole (Sigma Aldrich) for antifungal activity.

### Molecular docking

Twenty-three selected molecules were subjected to molecular docking analysis using the PLANTS program.^41^ The 23 compounds included in the molecular docking study were selected based on the chemical profiling of the *Suaeda confusa* and *Saponaria pumila* extracts, considering only the metabolites that were positively identified in the MeOH, water, and EA extracts. The three-dimensional structures of the selected proteins for the ten different microorganisms used in this study were retrieved from the RCSB Protein Data Bank.^42^ The selected targets included DNA gyrase (*E. coli*, PDB ID: 6RKS),^43^ arginine rhamnosyltransferase (*P. aeruginosa*, PDB ID: 6 J7J),^44^ tyrosyl-tRNA synthetase (*S. aureus*, PDB ID: 1JIJ),^45^ sphingomyelinase C (*B. cereus*, PDB ID: 2DDR),^46^ listeriolysin O (*L. monocytogenes*, PDB ID: 4CDB),^47^ thioredoxin reductase (*A. fumigatus*, PDB ID: 6BPY),^48^ endoglucanase A (*A. niger*, PDB ID: 1KS4),^49^ endo-1,4-β-xylanase C (*P. funiculosum*, PDB ID: 1TE1),^50^l-lysine oxidase (*T. viride*, PDB ID: 3X0V),^51^ and mono-/diacylglycerol lipase (*P. cyclopium*, PDB ID: 5CH8),^52^ which was also included as one of the target proteins. Molecular docking analysis was designed to investigate the potential interactions between the identified phytochemicals and biologically relevant microbial protein targets. Since the tested bacterial and fungal species do not share a common molecular target (AChE, BChE, α-amylase, α-glucosidase, and tyrosinase), representative proteins that play essential roles in microbial growth, survival, or virulence were selected for each microorganism. These targets are widely recognized in antimicrobial drug discovery, and were chosen to provide a mechanistic basis for the experimentally observed antimicrobial activity. The co-crystallized ligands present in the target protein structures were used as reference points to define the docking binding site. These native ligands served as guides for accurately determining the center of the binding site. Protein preparation was performed using the Dock Prep tool in UCSF Chimera,^53^ which involved removing non-standard residues and water molecules, adding hydrogen atoms, and assigning partial charges. The resulting protein–ligand complexes were visualized in PyMOL^54^ and further evaluated using BIOVIA Discovery Studio Visualizer (BIOVIA, 2020).

### Molecular dynamics (MD) simulations and free energy calculations

Molecular dynamics (MD) simulations were carried out using GROMACS^55^ to examine the structural stability and dynamic behavior of the docked protein–ligand complexes. For each protein, the five molecules with the highest docking scores were selected, and MD simulations were subsequently performed on these complexes. The AMBER99-SB force field^56^ was applied for the parameterization of the protein, while the ligand topology and parameters were generated using the Acpype tool.^57^ Each protein–ligand complex was embedded in a cubic simulation box with a minimum of 1.0 nm padding from the solute, solvated using TIP3P water, and neutralized by adding the necessary counterions. Before the production runs, all systems underwent energy minimization using the steepest-descent algorithm to remove any steric clashes. Equilibration was then performed in two steps: 250 ps under the NVT ensemble, followed by 250 ps under the NPT ensemble, during which positional restraints were applied to the heavy atoms. Temperature was regulated at 300 K using the V-rescale thermostat, while pressure was maintained at 1 bar with the Parrinello–Rahman barostat. Long-range electrostatics were computed using the Particle Mesh Ewald (PME) method, with cutoffs of 1.4 nm applied to both Coulomb and van der Waals interactions. Following equilibration, 100 ns production MD simulations were performed for each complex without restraints. The resulting trajectories were analyzed using standard GROMACS tools to evaluate the structural stability and flexibility, including the calculations of root mean square deviation (RMSD), root mean square fluctuation (RMSF), radius of gyration (Rg), and hydrogen bonding (H-bond) profiles. To quantify the binding energies of the protein–ligand complexes, binding free energy calculations were performed using the gmx_MMPBSA tool,^58^ which applies the Molecular Mechanics/Poisson–Boltzmann Surface Area (MM-PBSA) method. Binding free energies (ΔTOTAL) were estimated from snapshots extracted across the full simulation trajectory.

### Statistical analysis

All experiments were conducted in triplicate, and variations among extract samples were evaluated for statistical significance. The data were analyzed using one-way ANOVA (GraphPad Prism, version 9.2), followed by Tukey's post hoc test for pairwise group comparisons. A *p*-value below 0.05 was regarded as statistically significant. A Pearson correlation between total phenolic/flavonoid contents and biological activities was also performed using GraphPad Prism.

## Results and discussion

### Total phenolic and flavonoid contents

The total phenolic contents (TPC) and total flavonoid contents of plant species *S. pumila* and *S. confusa* were determined by the Folin assay and the AlCl_3_ assay, respectively. The results are given in Table 1. *S. pumila* MeOH extracts exhibited the highest TPC and TFC, measuring 44.82 mgGAE per g and 72.60 mgRE per g, respectively. The water extract of *S. pumila* showed TPC 40.62 mgGAE per g, while the EA extract recorded the lowest TPC, measuring 21.00 mgGAE per g. However, EA TFC (25.69 mgRE per g) was higher than that of the water extract (21.97 mgRE per g). Ozay *et al.*‘s investigation showed that the methanolic extracts of *S. kotschyi* and *S. Pumilio* exhibited greater levels of secondary metabolites and stronger antioxidant activity.^59^ In another species of the *Saponaria* genus, *S. officinalis*, acetone root extracts showed the highest content of total phenolics (17.813 mg GAE per g), yielding a value greater than those of methanolic (0.159 mg GAE per g) and ethanolic (0.973 mg GAE per g) extracts.^60^ This variation may be attributed to the inherent differences between the plant species.

**Table 1:** Extraction yields (%) and total phenolic and flavonoid contents in the tested extracts^a^

Species	Solvent	Extraction yield (%)	TPC (mgGAE per g)	TFC (mgRE per g)
*Saponaria pumila*	EA	11.58	21.00 ± 0.49^d^	25.69 ± 0.28^b^
	MeOH	13.45	44.82 ± 0.75^a^	72.60 ± 0.69^a^
	Water	9.24	40.62 ± 0.75^b^	21.97 ± 0.16^c^
*Suaeda confusa*	EA	2.00	23.43 ± 0.40^c^	17.80 ± 0.03^d^
	MeOH	24.11	15.05 ± 0.78^e^	13.92 ± 0.12^e^
	Water	21.06	12.30 ± 0.08^f^	8.33 ± 0.21^f^

a Values are reported as mean ± SD of three parallel measurements. GAE: gallic acid equivalents; RE: rutin equivalents. Different letters in the same column indicate significant differences among the tested extracts (*p* < 0.05).

In the case of *S. confusa*, the highest TPC (23.42 mgGAE per g) and TFC (17.80 mgRE per g) were recorded by the EA extract. In contrast, the lowest values, 12.30 mgGAE per g and 8.33 mgRE per g, respectively, were recorded by the water extract. The *S. confusa* MeOH extraction produced a moderate level (15.05 mgGAE per g and 13.92 mgRE per g, respectively), indicating species-dependent variation in the efficiency of the extraction solvent. A previous study^61^ has reported comparable findings, showing that higher TPC (11.09 mg GAE per g extract) and TFC (20.50 mg QE per g extract) were observed in the methanolic extracts of *S. fruticosa*.

### Phenolic profile by HPLC-ESI-Orbitrap-MS/MS

Ethyl acetate, methanolic, and water extracts of *Saponaria pumila* and *Suaeda confusa were* used to characterize and identify their biological activities. The non-targeted chemical characterization was performed through HPLC-ESI-Orbitrap-MS/MS. Chromatographic data, exact molecular mass/adducts, fragmentation patterns, isotopic distributions and on-line databases and own tandem mass spectral library (MS/MS) were applied to correctly identify unknown compounds in the extracts. The results are shown in Tables 2 and 3. All details for identified compounds are given in Tables S1–S6.

**Table 2:** Chemical profiles of the *Saponaria pumila* extracts (+: present; − absent)

Number	Compound	MeOH	Water	EA
1	Quinic acid	+	+	−
2	Citric acid	+	+	−
3	Catechin-*O*-hexoside	+	−	−
4	Tryptamine	−	+	−
5	Catechin	+	+	+
6	Epicatechin-*O*-hexoside	+	−	−
7	Caffeic acid	+	+	+
8	Vanillin (4-hydroxy-3-methoxybenzaldehyde)	+	−	+
9	Licoagroside B or isomer	+	+	+
10	Kynurenic acid	+	+	−
11	Epicatechin	+	+	+
12	Quercetin-di-*O*-rhamnosylhexoside isomer 1	−	+	−
13	Icariside B5 or isomer	+	−	+
14	Dihydroferulic acid (3-(4-hydroxy-3-methoxyphenyl)propionic acid)	+	+	+
15	Riboflavin	−	+	−
16	*p*-Coumaric acid	+	+	+
17	Phaselic acid (2-*O*-caffeoylmalic acid)	+	+	−
18	Abiesinol G (vitisinol) or isomer	+	−	+
19	Ferulic acid	+	+	+
20	Eriodictyol-*O*-hexoside	+	−	+
21	Sinapic acid	+	+	+
22	Quercetin-*O*-dirhamnosylhexoside	+	+	−
23	Quercetin-di-*O*-rhamnosylhexoside isomer 2	−	+	−
24	Quercetin-di-*O*-rhamnosylhexoside isomer 3	−	+	−
25	O-Benzyl-*O*-(3-hydroxy-3-methylglutaroyl)-hexose	+	+	+
26	Kaempferol-*O*-dirhamnosylhexoside	+	−	−
27	Quercetin-*O*-rhamnosyldihexoside	+	−	−
28	Naringenin-*O*-glucuronide	+	+	−
29	Hyperoside (quercetin-3-*O*-galactoside)	+	+	+
30	Rutin (quercetin-3-*O*-rutinoside)	+	+	+
31	Isoquercitrin (quercetin-3-*O*-glucoside)	+	+	+
32	Dimethoxycinnamic acid	+	+	+
33	Quercetin-*O*-pentoside isomer 1	+	−	−
34	Cosmosiin (apigenin-7-*O*-glucoside)	+	−	+
35	Azelaic acid (nonanedioic acid)	+	+	+
36	Chrysoeriol-7-*O*-glucoside	+	−	+
37	Quercetin-*O*-pentoside isomer 2	+	−	−
38	*O-p*-Coumaroyl-*O*-hydroxybenzoyl-rhamnosylhexoside	+	−	+
39	Eriodictyol (3′,4′,5,7-tetrahydroxyflavanone)	+	+	+
40	Astragalin (kaempferol-3-*O*-glucoside)	+	+	+
41	Apigenin-7-*O*-glucuronide	+	+	−
42	Kaempferol-3-*O*-rutinoside (nicotiflorin)	+	+	+
43	Isorhamnetin-*O*-hexoside	+	+	−
44	*O*-Feruloyl-*O*-hydroxybenzoyl-hexose	+	−	+
45	Isorhamnetin-3-*O*-rutinoside (narcissin)	+	+	+
46	Naringenin (4′,5,7-trihydroxyflavanone)	+	−	+
47	Quercetin (3,3′,4′,5,7-pentahydroxyflavone)	+	+	+
48	Luteolin (3′,4′,5,7-tetrahydroxyflavone)	+	+	+
49	Kaempferol (3,4′,5,7-tetrahydroxyflavone)	+	+	+
50	Isorhamnetin (3′-methoxy-3,4′,5,7-tetrahydroxyflavone)	+	+	+
51	Apigenin (4′,5,7-trihydroxyflavone)	+	+	+
52	Methyl-trihydroxyxanthone	+	−	+
53	Chrysoeriol (3′-methoxy-4′,5,7-trihydroxyflavone)	+	+	+
54	Malyngic acid (9,12,13-trihydroxy-10*E*,15*Z*-octadecadienoic acid)	+	+	+
55	Pinellic acid (9,12,13-trihydroxy-10*E*-octadecenoic acid)	+	+	+
56	Dihydroxy-methoxy(iso)flavone	+	−	+
57	Artemetin (5-hydroxy-3,3′,4′,6,7-pentamethoxyflavone)	+	−	−
58	Kumatakenin (4′,5-dihydroxy-3,7-dimethoxyflavone)	+	−	+
59	Hydroxy-trimethoxy(iso)flavone	+	−	+
60	Apigenin-4′,7-dimethyl ether (4′,7-dimethoxy-5-hydroxyflavone)	+	−	+
61	α-Linolenic acid	−	−	+
62	Palmitic acid	−	−	+
63	Oleic acid	−	−	+

**Table 3:** Chemical profiles of the *Suaeda confusa* extracts (+: present; −: absent)

Number	Compound	MeOH	Water	EA
1	Trigonelline	+	+	+
2	Citric acid	+	+	−
3	Uralenneoside	+	+	−
4	Caffeic acid	+	+	+
5	Vanillin (4-hydroxy-3-methoxybenzaldehyde)	+	−	+
6	Kynurenic acid	+	+	−
7	Tomenin (tomentin-5-*O*-glucoside)	+	−	+
8	12-Hydroxyjasmonic acid or tuberonic acid	+	+	+
9	*p*-Coumaric acid	+	+	+
10	*N-cis*-Feruloyloctopamine	+	+	+
11	Indole-3-carbaldehyde	−	−	+
12	Indole-3-carboxylic acid	−	−	+
13	Ferulic acid	+	+	+
14	*p*-Coumaroylmalic acid	+	+	−
15	Isorhamnetin-di-*O*-hexoside isomer 1	+	+	−
16	Isorhamnetin-di-*O*-hexoside isomer 2	+	+	−
17	*N-trans*-Feruloyloctopamine	+	+	+
18	Isorhamnetin-3-*O*-rutinoside-7-*O*-glucoside	+	+	−
19	Tomentin (6,7-dimethoxy-5-hydroxycoumarin)	−	+	+
20	6,7-Dihydroxycoumarin-3-carboxylic acid	+	+	+
21	*N-cis*-Feruloyltyramine	+	−	+
22	*N-trans*-Caffeoyltyramine	+	−	+
23	Isorhamnetin-3-*O*-robinobioside	+	+	+
24	Hyperoside (quercetin-3-*O*-galactoside)	+	+	+
25	Isoquercitrin (quercetin-3-*O*-glucoside)	+	+	+
26	Rutin (quercetin-3-*O*-rutinoside)	+	+	+
27	Jaslanceoside B	+	+	+
28	Quercetin-3-*O*-(6″-malonyl)glucoside	+	−	−
29	*N-trans*-Feruloyltyramine	+	+	+
30	Abscisic acid	−	−	+
31	Isorhamnetin-3-*O*-glucoside	+	+	+
32	Isorhamnetin-3-*O*-rutinoside (narcissin)	+	+	+
33	Naringenin (4′,5,7-trihydroxyflavanone)	+	+	+
34	Homoeriodictyol (3′-methoxy-4′,5,7-trihydroxyflavanone)	+	−	+
35	Quercetin (3,3′,4′,5,7-pentahydroxyflavone)	+	−	+
36	Luteolin (3′,4′,5,7-tetrahydroxyflavone)	+	−	+
37	Isorhamnetin (3′-methoxy-3,4′,5,7-tetrahydroxyflavone)	+	−	+
38	Chrysoeriol (3′-methoxy-4′,5,7-trihydroxyflavone)	+	−	+
39	Traumatic acid (2-dodecenedioic acid)	+	−	+
40	Traumatic acid isomer	+	−	+
41	α-Linolenic acid	−	−	+
42	Linoleic acid	−	−	+
43	Palmitic acid	−	−	+
44	Oleic acid	−	−	+

The chemical composition of *Saponaria pumila* extracts: the extracts showed similar chromatographic profiles, and a wide range of compounds were characterized (Table 2). The methanolic extract contained a greater number of chemical compounds (55 compounds). 40 compounds were detected in the aqueous extract. In the ethyl acetate extract, 43 compounds were identified. There were 26 compounds that were identified in all three extracts, which are as follows: catechin, caffeic acid, licoagroside B or isomer, epicatechin, dihydroferulic acid (3-(4-hydroxy-3-methoxyphenyl)propionic acid), phaselic acid (2-*O*-caffeoylmalic acid), ferulic acid, sinapic acid, *O*-benzyl-*O*-(3-hydroxy-3-methylglutaroyl)-hexose, hyperoside (quercetin-3-*O*-galactoside), rutin (quercetin-3-*O*-rutinoside), isoquercitrin (quercetin-3-*O*-glucoside), dimethoxycinnamic acid, azelaic acid (nonanedioic acid), eriodictyol (3′,4′,5,7-tetrahydroxyflavanone), kaempferol-3-*O*-rutinoside (nicotiflorin), isorhamnetin-3-*O*-rutinoside (narcissin), naringenin (4′,5,7-trihydroxyflavanone), quercetin (3,3′,4′,5,7-pentahydroxyflavone), luteolin (3′,4′,5,7-tetrahydroxyflavone), kaempferol (3,4′,5,7-tetrahydroxyflavone), isorhamnetin (3′-methoxy-3,4′,5,7-tetrahydroxyflavone), apigenin (4′,5,7-trihydroxyflavone), methyl-trihydroxyxanthone, chrysoeriol (3′-methoxy-4′,5,7-trihydroxyflavone), malyngic acid (9,12,13-trihydroxy-10*E*,15*Z*-octadecadienoic acid), and pinellic acid (9,12,13-trihydroxy-10*E*-octadecenoic acid).

Ethyl acetate, methanol, and water extracts of *Suaeda confusa*: these extracts also showed similar chromatographic profiles. The employed untargeted molecular profiling results showed the same number (36–36) of components in the methanolic and ethyl acetate extracts and only 25 compounds were characterized in the aqueous extract. The results are shown in Table 3. There were 17 compounds that were identified in all three extracts, which are as follows: trigonelline, caffeic acid, 12-hydroxyjasmonic acid, *p*-coumaric acid, *N-cis*-feruloyloctopamine, ferulic acid, *N-trans*-feruloyloctopamine, 6,7-dihydroxycoumarin-3-carboxylic acid, isorhamnetin-3-*O*-robinobioside, hyperoside (quercetin-3-*O*-galactoside), isoquercitrin (quercetin-3-*O*-glucoside), rutin (quercetin-3-*O*-rutinoside), jaslanceoside B, *N-trans*-feruloyltyramine, isorhamnetin-3-*O*-glucoside, isorhamnetin-3-*O*-rutinoside (narcissin), and naringenin (4′,5,7-trihydroxyflavanone). In addition to these, similar compounds were identified in some samples, mainly acids (citric, kynurenic, traumatic, linoleic, palmitic, oleic), quercetin, luteolin, isorhamnetin, and chrysoeriol.

### Antioxidant properties

The antioxidant activities of both plants are given in Table 4. In *S. pumila*, the MeOH extracts consistently exhibited the highest antioxidant capacity across most assays, with the highest value recorded in CUPRAC (401.36 mg TE per g), FRAP (297.00 mg TE per g), ABTS (160.40 mg TE per g), and DPPH (65.36 mg TE per g), reflecting its highest TPC and TFC. The water extraction exhibited a DPPH activity of 58.93 mg TE per g, moderately close to MeOH extract and ABTS (160.56 mg TE per g), nearly similar to MeOH extracts, despite its comparatively low TPC and TFC. The increased activity is mainly due to the ability of MeOH to solidly extract bioactive compounds, particularly phenolic compounds, which are renowned antioxidants.^62^ Several studies have revealed that MeOH and water extracts exhibit excellent antioxidant activities and are generally more potent than extracts.^63–65^ On the other hand, this study found that the EA extract comparatively recorded the lowest antioxidant activity in DPPH, ABTS, CUPRAC, and FRAP assays. In MCA and PBD assays, the EA extract recorded peak antioxidant values, measuring 36.25 mg EDTAE per g and 2.90 mmol TE per g, respectively. This pattern indicated that phenolic and flavonoid contents strongly contributed to radical scavenging and reducing power activity, while having less to metal chelating and PBD, supporting the previous study of Ahmed *et al.*^66^ Previously, the *S. officinalis* acetone extract of root showed the highest antioxidant activity in SH-SY5Y cells, then ethanolic and methanolic extracts, which showed a relatively poor activity.^60^

**Table 4:** Antioxidant properties of the tested extracts^a^

Species	Solvents	DPPH (mg TE per g)	ABTS (mg TE per g)	CUPRAC (mg TE per g)	FRAP (mg TE per g)	Chelating (mg EDTAE per g)	PBD (mmol TE per g)
*Saponaria pumila*	EA	21.52 ± 1.43^c^	66.72 ± 2.50^c^	116.33 ± 1.90^c^	58.86 ± 4.23^c^	36.25 ± 0.90^a^	2.90 ± 0.11^a^
	MeOH	65.36 ± 0.13^a^	160.40 ± 0.29^a^	401.36 ± 21.27^a^	297.00 ± 14.21^a^	29.84 ± 0.61^c^	2.15 ± 0.12^b^
	Water	58.93 ± 1.69^b^	160.56 ± 0.11^a^	219.95 ± 7.98^b^	188.40 ± 5.16^b^	30.31 ± 0.14^bc^	1.39 ± 0.01^d^
*Suaeda confusa*	EA	8.33 ± 1.10^f^	41.78 ± 1.59^e^	69.55 ± 1.74^e^	38.27 ± 5.18^d^	36.46 ± 0.23^a^	1.89 ± 0.03^c^
	MeOH	17.27 ± 0.09^d^	59.13 ± 0.65^d^	106.53 ± 2.36 ^cd^	69.31 ± 1.09^c^	13.15 ± 1.44^d^	1.32 ± 0.02^d^
	Water	13.37 ± 0.90^e^	75.70 ± 3.68^b^	83.28 ± 1.47^de^	68.74 ± 2.28^c^	32.34 ± 0.46^b^	0.51 ± 0.06^e^

a Values are reported as mean ± SD of three parallel measurements. TE: Trolox equivalents; EDTAE: EDTA equivalent. Different letters in the same column indicate significant differences among the tested extracts (*p* < 0.05).

In the case of the plant species *S. confusa*, MeOH extracts again yielded the highest value in radical scavenging and reducing power assays in the same pattern as in *S. pumila*. The antioxidant value recorded 106.53 mg TE per g in CUPRAC, 69.31 mg TE per g in FRAP, and 59.13 mg TE per g in DPPH. Water extracts showed comparatively the highest ABTS scavenging activity, recorded 75.70 mg TE per g, and in the FRAP assay, the antioxidant activity recorded 68.74 mg TE per g close to the MeOH value. Saleem *et al.*^67^ reported the highest radical scavenging and reducing power of *S. fruticosa* methanolic extract.

### Enzyme inhibitory properties

Enzyme inhibition is a key therapeutic approach for managing various health issues, including Alzheimer's,^68,69^ diabetes,^70^ and skin disorders.^71^ The enzyme inhibitory activities of *S. pumila* varied noticeably among the solvent extracts. The results are given in Table 5. The water extract exhibited the strongest inhibitory effect against AChE, with a concentration of 2.00 mg GALAE per g, highlighting its neuroprotective effect.^69^ However, this plant EA extract showed no AChE inhibition, while the highest BChE inhibitory activity was measured at 1.87 mg GALAE per g, compared to other extracts. In the case of tyrosinase inhibitory activity, the water extract exhibited the highest inhibitory activity, with the values of 42.47 mg KAE per g. On the other hand, the MeOH extract exhibited a moderate activity (31.36 mg KAE per g) comparatively. Previous studies have also revealed that aqueous extracts of *S. pumila* are used in cosmetics as a result of their impressive dermo-protective activity.^72^ In the case of α-amylase and α-glucosidase, all the tested extracts showed a relatively low inhibitory activity. EA and MeOH extracts showed significantly the highest anti-α-amylase activity, measuring 0.96 mmol ACAE per g and 0.55 mmol ACAE per g, respectively, while the water extract showed a progressively lower inhibitory activity. Similarly, for α-glucosidase inhibition, the MeOH extract showed a higher inhibitory activity, 0.68 mmol ACAE per g. The significant inhibition of α-amylase and α-glucosidase indicates that *S. pumila* extracts may play a key role in managing metabolic disorders, such as obesity and diabetes.^73^ These results are consistent with a previous study by Chonsut *et al.*,^74^ which found that the alcoholic extracts of the five flowers of various plants exhibited strong α-amylase inhibitory activity. However, a notable difference was observed for α-glucosidase inhibition; their aqueous extracts exhibited stronger inhibitory activity.

**Table 5:** Enzyme inhibitory properties of the tested extracts^a^

Species	Solvents	AChE (mg GALAE per g)	BChE (mg GALAE per g)	Tyrosinase (mg KAE per g)	Amylase (mmol ACAE per g)	Glucosidase (mmol ACAE per g)
*Saponaria pumila*	EA	Na	1.87 ± 0.12^a^	25.62 ± 6.78^c^	0.96 ± 0.03^a^	0.18 ± 0.08^b^
	MeOH	1.50 ± 0.13^c^	0.38 ± 0.05^c^	31.36 ± 2.19^bc^	0.55 ± 0.01^c^	0.68 ± 0.03^a^
	Water	2.00 ± 0.01^b^	1.06 ± 0.09^b^	42.47 ± 0.84^a^	0.20 ± 0.01^e^	0.26 ± 0.04^b^
*Suaeda confusa*	EA	1.46 ± 0.18^c^	1.58 ± 0.19^a^	37.83 ± 0.71 ^ab^	0.68 ± 0.01^b^	Na
	MeOH	2.59 ± 0.01^a^	0.99 ± 0.07^b^	46.28 ± 5.77^a^	0.42 ± 0.01^d^	Na
	Water	1.76 ± 0.02^b^	0.20 ± 0.01^d^	46.34 ± 1.36^a^	0.10 ± 0.01^f^	Na

a Values are reported as mean ± SD of three parallel measurements. GALAE: Galantamine equivalent; KAE: Kojic acid equivalent; ACAE: Acarbose equivalent; and na: not active. Different letters in the same column indicate significant differences among the tested extracts (*p* < 0.05).

*S. confusa* enzyme inhibitory activities are given in Table 5. For the species *S. confusa*, MeOH extracts exhibited the highest inhibition of AChE (2.59 mg GALAE per g) and tyrosinase (46.28 mg KAE per g), along with moderate BChE inhibition (0.99 mg GALAE per g). A previous study of Saleem *et al.*,^67^ recorded that the *S. fruticosa* extracts revealed the highest levels of tyrosinase inhibition, measuring 25.38 mg KAE per g.^61^ These results suggest that the extraction solvents enhance phenolic extraction, with MeOH also being effective in promoting AChE and tyrosinase inhibition. Collectively, these findings indicate that *S. confusa* possesses both neuroprotective^69^ and dermoprotective properties.^71^ All three tested extracts showed no activity against α-glucosidase, but limited α-amylase inhibitory activity.

Correlation analysis (Fig. 1) revealed a strong positive relationship between TPC and glucosidase inhibition (*r* = 0.86) and between TFC and glucosidase inhibition (*r* = 0.96), suggesting that phenolic and flavonoid compounds contribute substantially to the glucosidase inhibitory activity. In contrast, TPC and TFC showed weak to moderate negative correlations with tyrosinase inhibition (*r* = −0.36 and *r* = −0.58, respectively) and negligible correlations with acetylcholinesterase (AChE) inhibition (*r* = −0.01 and *r* = −0.19, respectively).

**Fig. 1:**
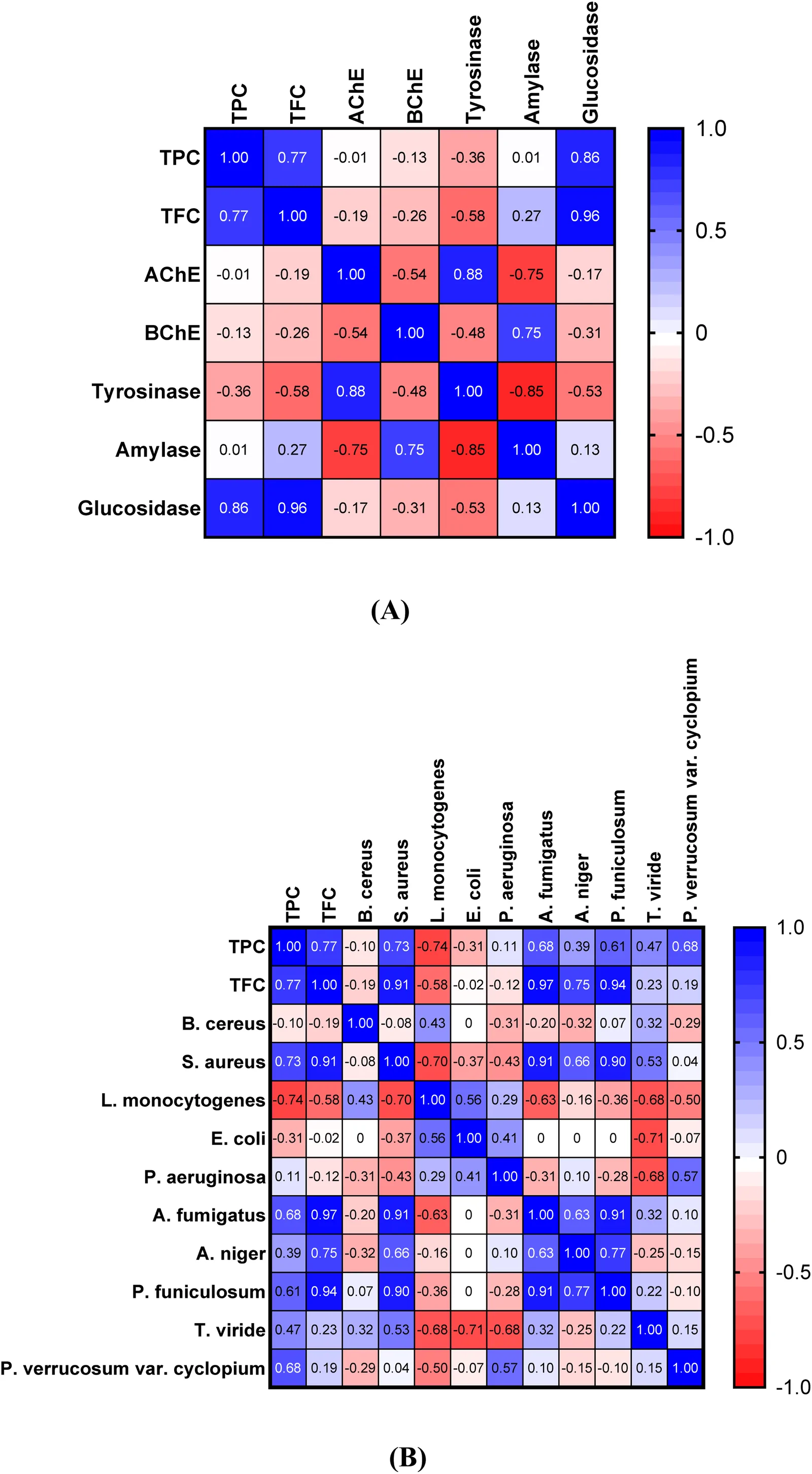
Pearson correlation analysis (A) between the total phenolic/flavonoid content and enzyme inhibitory effects and (B) between the total phenolic/flavonoid content and antimicrobial effects.

### Antimicrobial activity

The antimicrobial potential of *Saponaria pumila* and *Suaeda confusa* extracts was evaluated against a panel of Gram-positive and Gram-negative bacteria, as well as filamentous fungi. The results showed that all extracts exhibited measurable antimicrobial activity, although the degree of inhibition varied considerably depending on the plant species, solvents used, and microbial strain tested. These differences indicate that the antimicrobial activity of the extracts is strongly influenced by their chemical composition, which is determined by the selectivity of the extraction solvent.

The antibacterial activity (Table 6) of the extracts was characterized by the MIC values ranging from 0.25 to 4.0 mg mL^−1^, while the MBC values ranged from 0.5 to 8.0 mg mL^−1^. Although the activity was lower than that of conventional antibiotics used as positive controls, these differences are expected given the use of crude plant extracts. Among the tested microorganisms, *Listeria monocytogenes* showed the highest susceptibility to the extracts. The lowest MIC values were recorded for *S. pumila* ethyl acetate extract and *S. confusa* ethyl acetate and methanolic extracts, with MIC/MBC values of 0.25/0.50 mg mL^−1^. The activity against *Bacillus cereus* was also notable, particularly for the ethyl acetate extract of *S. confusa*, which exhibited MIC and MBC values of 0.50 and 1.0 mg mL^−1^, respectively. In contrast, *Staphylococcus aureus* demonstrated higher resistance, requiring higher extract concentrations for growth inhibition. These differences among Gram-positive bacteria indicate that susceptibility is species-specific and likely related to variations in cell wall structure, membrane composition, and microbial defense mechanisms.

**Table 6:** Antibacterial activity of the extracts of the *Saponaria pumila* and *Suaeda confusa* species

Sample	Concentration (mg mL^−1^)									
	*B. cereus*		*S. aureus*		*L. monocytogenes*		*E. coli*		*Pseudomonas aeruginosa*	
	MIC	MBC	MIC	MBC	MIC	MBC	MIC	MBC	MIC	MBC
*S. pumila*-EA	1.00	2.00	2.00	4.00	0.25	0.50	0.50	1.00	1.00	2.00
*S. pumila*-MeOH	1.00	2.00	2.00	4.00	0.50	1.00	1.00	2.00	2.00	4.00
*S. pumila*-water	1.00	2.00	2.00	4.00	1.00	2.00	1.00	2.00	1.00	2.00
*S*. *confusa*-EA	0.50	1.00	2.00	4.00	0.25	0.50	0.50	1.00	2.00	4.00
*S*. *confusa*-MeOH	1.00	2.00	4.00	8.00	0.25	0.50	0.25	0.50	1.00	2.00
*S*. *confusa*-water	1.00	2.00	2.00	4.00	0.50	1.00	1.00	2.00	4.00	8.00

Control										
Streptomycin	0.02	0.05	0.10	0.20	0.15	0.30	0.10	0.20	0.10	0.20
Ampicillin	0.10	0.15	0.10	0.15	0.15	0.50	0.15	0.20	0.05	0.10

The extracts also displayed activity against Gram-negative bacteria, although with greater variability. The strongest inhibition of *Escherichia coli* was observed for *S. confusa* methanolic extract (MIC/MBC 0.25/0.50 mg mL^−1^) and *S. pumila* ethyl acetate extract (MIC/MBC 0.50/1.0 mg mL^−1^). Conversely, *Pseudomonas aeruginosa* was among the least susceptible strains, consistent with the well-described intrinsic resistance of this species associated with its low outer membrane permeability and efficient efflux mechanisms. Ethyl acetate extracts of both plant species demonstrated consistent bactericidal activity across several bacterial strains.

The antifungal evaluation (Table 7) revealed that both plant species possessed broad-spectrum activities against the tested filamentous fungi, with the MIC values ranging from 0.5 to 4 mg mL^−1^ and the MFC values from 1 to ≥8 mg mL^−1^. Among all samples, *S. pumila* methanolic extract showed the most pronounced antifungal activity, exhibiting MIC values of 0.5 mg mL^−1^ against *Aspergillus fumigatus* and *Penicillium funiculosum*. The enhanced activity of the methanolic extract suggests that moderately polar constituents, such as phenolic compounds, flavonoids, and saponin-related metabolites, may contribute substantially to antifungal effects. The aqueous extract of *S. pumila* also showed considerable activity, particularly against *Trichoderma viride* (MIC/MFC 0.5/1 mg mL^−1^), indicating that water-soluble metabolites may also contribute to the antifungal properties. In comparison, *S. confusa* extracts showed moderate antifungal activity, with the ethyl acetate extract displaying the most consistent inhibition across the tested fungal species.

**Table 7:** Antifungal activity of the extracts of the *Saponaria pumila* and *Suaeda confusa* species

Sample	Concentration (mg mL^−1^)									
	*A. fumigatus*		*A. niger*		*P. funiculosum*		*T. viride*		*P. verrucosum* var. *cyclopium*	
	MIC	MFC	MIC	MFC	MIC	MFC	MIC	MFC	MIC	MFC
*S. pumila*-EA	1	2	1	2	1	2	2	4	2	4
*S. pumila*-MeOH	0.5	1	1	2	0.5	1	1	2	1	2
*S. pumila*-water	2	4	2	4	1	2	0.5	1	1	2
*S*. *confusa*-EA	1	2	2	4	1	2	1	2	2	4
*S*. *confusa*-MeOH	1	2	2	4	2	4	2	≥8	1	2
*S*. *confusa*-water	1	2	2	4	2	4	1	2	4	8

**Control**										
Ketoconazole	0.20	0.50	0.20	0.50	0.20	0.50	1.00	1.50	0.20	0.30
Bifonazole	0.15	0.20	0.1	0.20	0.20	0.2	0.15	0.20	0.10	0.20

The results demonstrate that *S. pumila* and *S. confusa* represent promising sources of antimicrobial metabolites. The distinct activity profiles observed among solvents highlight the importance of extraction strategy in recovering bioactive compounds. The stronger antifungal performance of *S. pumila* methanolic extract and the antibacterial potential of *S. confusa* ethyl acetate and methanolic extracts suggest that these fractions may be suitable candidates for further phytochemical characterization and bioactivity-guided fractionation.

Correlation analysis (Fig. 1) of the antimicrobial activity data showed that the total phenolic content (TPC) and total flavonoid content (TFC) were strongly positively correlated with each other (*r* = 0.77) and with activity against several test organisms, particularly *S. aureus* (*r* = 0.73 and 0.91), *A. fumigatus* (*r* = 0.68 and 0.97), and *P. funiculosum* (*r* = 0.61 and 0.94), while both were strongly negatively correlated with *L. monocytogenes* (*r* = −0.74 and −0.58), indicating that the phenolic and flavonoid compounds are major contributors to antibacterial and antifungal activities, but may be less effective, or act through a different mechanism, against *L. monocytogenes*.

### Molecular docking

Thioredoxin reductase from *A. fumigatus* plays a central role in maintaining fungal redox homeostasis, and is therefore recognized as a compelling antifungal drug target.^75^ In this study, docking analyses performed on 23 candidate molecules (Table S7) revealed a broad spectrum of predicted binding affinities, ranging from the least favorable interaction with isoquercitrin (−71.68 kcal mol^−1^) to the strongest affinity observed for pinellic acid (−105.43 kcal mol^−1^). As expected, more negative binding energies reflect a higher predicted binding strength and a potentially greater inhibitory activity.^76^ Among all the tested compounds, pinellic acid emerged as the top candidate, closely followed by malyngic acid (−99.63 kcal mol^−1^) and jaslanceoside B (−93.88 kcal mol^−1^), highlighting these three molecules as particularly promising inhibitors of *A. fumigatus* thioredoxin reductase. The 2D interaction diagrams generated for the top-performing molecules (Fig. S1) further support these findings by illustrating both shared and distinct features in their binding modes. Icariside B5 (Fig. S1a), jaslanceoside B (Fig. S1b), malyngic acid (Fig. S1c), and pinellic acid (Fig. S1d) consistently form stabilizing hydrogen bonds primarily involving residues such as Asn125, Ala121, and Thr49. Additionally, jaslanceoside B and pinellic acid engage in notable Pi–Alkyl contacts with Pro13 and Ala303, which may further enhance the binding stability. In contrast, quercetin (Fig. S1e) interacts with residues including Ser11, Gly12, Ala121, and Ser306, forming fewer hydrogen bonds but displaying a pronounced Pi–anion interaction with Asp294 likely facilitated by its planar aromatic scaffold. Overall, the hydrogen bonds and Pi-mediated interactions observed across these top candidates demonstrate their effective engagement with the enzyme's binding site. Taken together, these results underscore the potential of pinellic acid, malyngic acid, and jaslanceoside B as lead molecules for the future development of novel antifungal agents targeting thioredoxin reductase in *A. fumigatus*.

Endoglucanase A from *A. niger* is a central enzyme in cellulose degradation,^77^ and therefore, represents an important molecular target for inhibitor discovery. Docking analyses of 23 test molecules against this enzyme revealed a wide range of predicted affinities, from the weakest interaction observed for tomentin (−51.59 kcal mol^−1^) to the strongest for rutin (−99.52 kcal mol^−1^) (Table S7). Among all ligands, rutin clearly stands out with the most favorable predicted binding energy, followed by malyngic acid (−92.81 kcal mol^−1^) and jaslanceoside B (−90.34 kcal mol^−1^). In contrast, molecules such as tomentin, dimethoxycinnamic acid, and dihydroferulic acid exhibit much weaker predicted binding, consistent with their substantially less favorable docking scores. The 2D interaction diagrams for the five best-scoring molecules (Fig. S2) further illustrate the key features underlying their stable binding modes. Isoquercitrin (Fig. S2a), jaslanceoside B (Fig. S2b), malyngic acid (Fig. S2c), pinellic acid (Fig. S2d), and rutin (Fig. S2e) all rely on a conserved network of van der Waals contacts and hydrogen bonds with residues such as Asp90, Thr199, and Gln154, supporting their effective anchoring within the binding site. Additionally, jaslanceoside B, malyngic acid, and pinellic acid form Pi–Alkyl and Pi–Sigma interactions with Val56, Phe202, and Met114, which may further stabilize their binding orientations. Among these top binders, rutin is particularly notable due to its structural complexity and extensive interaction profile. It forms multiple hydrogen bonds with Thr199, Gly21, Gln154, Glu200, and Ser107, while also engaging in both Pi interactions with Val56 and an ionic interaction with the Pd atom (grey line). This rich interaction network suggests a more specific and deeply embedded binding mode, consistent with its docking score. Overall, the docking and interaction analyses point to rutin, malyngic acid, and jaslanceoside B as the most promising inhibitors of *A. niger* endoglucanase A, providing valuable leads for future antifungal or enzyme-targeted studies. Sphingomyelinase C from *B. cereus*, a key virulence factor,^78^ was also evaluated as a molecular target for the 23 molecules. The docking scores for this enzyme show a narrow distribution compared to the other targets, ranging from the weakest predicted interaction with tomentin (−54.28 kcal mol^−1^) to the strongest with pinellic acid (−91.99 kcal mol^−1^) (Table S7). Pinellic acid emerges as the top predicted inhibitor, followed closely by malyngic acid (−89.90 kcal mol^−1^) and azelaic acid (−76.56 kcal mol^−1^). In contrast, tomentin and dimethoxycinnamic acid exhibit the least favorable binding energies and are therefore less likely to establish a stable interaction with the enzyme. The 2D interaction diagrams for the five best-scoring ligands (Fig. S3) provide deeper insights into their binding behavior within the sphingomyelinase C binding site. Azelaic acid (Fig. S3a), icariside B5 (Fig. S3c), and pinellic acid (Fig. S3e) each form stabilizing hydrogen bonds with polar and charged residues such as Asp278, His141, and Asn180. These molecules also prominently engage in Pi-based interactions with aromatic and hydrophobic residues including Trp215, Phe227, and Tyr19, helping to anchor them more firmly within the pocket. Additionally, azelaic acid, icariside B5, malyngic acid (Fig. S3d), and pinellic acid all display a conserved ionic interaction with the Ca atom (grey lines), indicating a common mode of coordination with a metal ion cofactor that may contribute to binding stability. Catechin (Fig. S3b), while still forming hydrogen bonds with Asp278 and His279, appears to adopt a slightly different orientation within the binding site. Its interaction pattern is dominated by Pi–Pi stacking with Phe227 and Trp215, suggesting a preference for aromatic stabilization rather than direct coordination with the Ca ion. Overall, these interaction patterns highlight pinellic acid, malyngic acid, and azelaic acid as the most promising inhibitors of *B. cereus* sphingomyelinase C. Their hydrogen-bond networks, Pi-mediated contacts, and conserved Ca-ion coordination collectively point to their potential utility as lead compounds for targeting this virulence-associated enzyme. The molecular docking analysis performed on *E. coli* DNA gyrase, an essential enzyme for DNA replication and repair,^79^ reveals a moderate yet meaningful variation in predicted binding affinities among the screened compounds. Docking scores span from the weakest predicted interaction with tomentin (−47.01 kcal mol^−1^) to the strongest with malyngic acid (−90.06 kcal mol^−1^) (Table S7). This ∼37 kcal mol^−1^ difference indicates a high degree of structural selectivity within the enzyme's binding site. Malyngic acid exhibits the most favorable predicted affinity, highlighting it as a leading inhibitor candidate, closely followed by pinellic acid (−86.84 kcal mol^−1^) and jaslanceoside B (−80.52 kcal mol^−1^). On the other end of the spectrum, tomentin and dimethoxycinnamic acid yield weak docking scores, suggesting insufficient steric or electrostatic complementarity for stable binding. The 2D interaction graph for the five molecules with the lowest docking score docked with *E. coli* DNA gyrase is given in Fig. S4. Isoquercitrin (Fig. S4a), jaslanceoside B (Fig. S4b), malyngic acid (Fig. S4c), and pinellic acid (Fig. S4d) form key hydrogen bonds with residues such as Asp783 and Ser784, underscoring the critical role of these residues in ligand stabilization. Additionally, Pi-mediated contacts with hydrophobic or aromatic residues, including Ile775, Met821, and Ala112, contribute to the overall binding affinity of these complexes. Notably, isoquercitrin's planar aromatic scaffold enables an extra Pi–anion interaction with Met113, differentiating its binding mode from the others and likely enhancing its stability within the binding site. Listeriolysin O from *L. monocytogenes* is a key pore-forming toxin essential for bacterial virulence.^80^ The docking results show a broad range of predicted affinities, from the least favorable score of −48.05 kcal mol^−1^ (tomentin) to the most favorable −74.61 kcal mol^−1^ (pinellic acid). Pinellic acid demonstrates the strongest predicted binding and thus appears to be the most promising inhibitor candidate, followed by malyngic acid (−71.67 kcal mol^−1^) and isoquercitrin (−70.65 kcal mol^−1^) (Table S7). In contrast, dimethoxycinnamic acid and tomentin exhibit weak predicted interactions, suggesting poor structural or electrostatic complementarity with the toxin's binding site. The 2D interaction diagrams for the top-scoring molecules (Fig. S5) indicate that binding is predominantly mediated by hydrogen bonds, reflecting polar anchoring within the pocket. Astragallin (Fig. S5a), isoquercitrin (Fig. S5b), malyngic acid (Fig. S5c), pinellic acid (Fig. S5d), and rutin (Fig. S5e) each form multiple H-bonds with key polar and charged residues, particularly Asp458, Asp459, and Thr460. The more structurally complex molecules (astragallin, isoquercitrin, and rutin) maximize these interactions through their numerous hydroxyl and carbonyl groups, supporting their favorable binding modes. The molecular docking analysis of *P. aeruginosa* arginine rhamnosyltransferase, an enzyme associated with antibiotic resistance, reveals a wide distribution of predicted binding affinities (Table S8). Docking scores range from the weakest interaction with tomentin (−54.29 kcal mol^−1^) to the strongest with pinellic acid (−85.06 kcal mol^−1^), identifying the latter as the most promising inhibitor candidate. Malyngic acid (−80.99 kcal mol^−1^) and icariside B5 (−73.76 kcal mol^−1^) also demonstrate predicted interactions, whereas tomentin, apigenin (−58.71 kcal mol^−1^), and kaempferol (−59.57 kcal mol^−1^) show comparatively weak affinity, likely due to the poor geometric compatibility or insufficient stabilizing contacts. The 2D interaction diagrams (Fig. S6) indicate that the binding site exhibits a pronounced preference for hydrogen bonding, particularly with polar and charged residues such as Arg184, Glu183, and Gln168. All five analyzed molecules, azelaic acid (Fig. S6a), dihydroferulic acid (Fig. S6b), icariside B5 (Fig. S6c), malyngic acid (Fig. S6d), and pinellic acid (Fig. S6e), establish multiple H-bonds that effectively anchor them within this polar pocket. While azelaic acid, dihydroferulic acid, and malyngic acid rely almost exclusively on these polar interactions, icariside B5 additionally forms Pi-based contacts through its aromatic ring, suggesting a more diverse stabilization strategy that engages both polar and hydrophobic regions. The docking analysis of 23 molecules against mono-/diacylglycerol lipase from *P. cyclopium*, an enzyme central to fungal lipid metabolism, reveals substantial variation in predicted inhibitory potential (Table S8). Docking scores span from the weakest interaction with dimethoxycinnamic acid (−56.43 kcal mol^−1^) to the strongest with pinellic acid (−81.80 kcal mol^−1^), identifying the latter as the most promising inhibitor candidate. Malyngic acid (−77.60 kcal mol^−1^) and icariside B5 (−71.76 kcal mol^−1^) also exhibit binding potential. The 2D interaction diagrams (Fig. S7) show that the binding site strongly favors hydrogen bonding, particularly involving residues Thr61 and Thr63. Aromatic molecules such as eriodictyol (Fig. S7a) and luteolin (Fig. S7c) display enhanced stabilization through prominent Pi–anion interactions with Asp62, where their flavonoid π-systems align closely with the residue's negatively charged carboxylate group. In comparison, icariside B5 (Fig. S7b) relies on H-bonds complemented by a Pi–alkyl contact with Trp89 and Leu146. The non-aromatic ligands malyngic acid (Fig. S7d) and pinellic acid (Fig. S7e) compensate for the absence of Pi interactions by forming a higher number of direct hydrogen bonds with residues such as Trp89, Gly93, and Ser115, resulting in their favorable overall binding. The docking analysis of endo-1,4-β-xylanase C from *P. funiculosum*, a key fungal enzyme involved in xylan degradation,^81^ revealed a broad range of predicted inhibitory potentials across the 23 molecules. Docking scores varied substantially, from the weakest interaction observed for rutin (−57.45 kcal mol^−1^) to the strongest for malyngic acid (−105.54 kcal mol^−1^), positioning the latter as the most promising inhibitor candidate (Table S8). Pinellic acid (−97.88 kcal mol^−1^) and epicatechin (−90.16 kcal mol^−1^) also displayed robust predicted affinities, whereas rutin and jaslanceoside B (−62.17 kcal mol^−1^) showed minimal binding propensity. Analysis of the 2D interaction profiles for five representative molecules (Fig. S8) indicated a conserved binding pattern dominated by both polar and hydrophobic contacts. All molecules (catechin, epicatechin, eriodictyol, malyngic acid, and pinellic acid) formed hydrogen bonds with Glu177, highlighting its role as a central anchoring residue. In addition, each compound engaged in Pi–Pi stacking with Trp19, providing notable hydrophobic stabilization. Epicatechin (Fig. S8b) and eriodictyol (Fig. S8c) further exhibited a Pi–anion interaction with Glu86, suggesting an additional stabilizing feature that may enhance their binding orientation. The molecular docking analysis of *S. aureus* tyrosyl-tRNA synthetase, an essential enzyme in protein biosynthesis,^82^ revealed a wide range of predicted binding affinities. Docking scores extended from the weakest interaction observed for jaslanceoside B (−60.87 kcal mol^−1^) to the strongest for pinellic acid (−101.27 kcal mol^−1^), identifying pinellic acid as the most promising inhibitor candidate (Table S2). Malyngic acid (−100.64 kcal mol^−1^) and icariside B5 (−86.56 kcal mol^−1^) also displayed predicted affinities, whereas jaslanceoside B and tomentin (−63.92 kcal mol^−1^) exhibited minimal binding potential. Analysis of the 2D interaction graphs (Fig. S9) showed that ligand engagement within the binding site is mediated by a combination of hydrogen bonding, π-interactions, and charge-based contacts. All five representative molecules (hyperoside, icariside B5, malyngic acid, pinellic acid, and rutin) form hydrogen bonds with key residues such as Gly192, Gly37, and Asp194. Hyperoside (Fig. S9a) additionally establishes a notable Pi–anion interaction with Asp79, anchoring its aromatic ring near the negatively charged residue. In contrast, the more aliphatic malyngic acid (Fig. S9c) and pinellic acid (Fig. S9d) form multiple Pi-interactions with hydrophobic residues including His49, Phe53, Ala38, and Leu69. The molecular docking analysis of *T. viride*l-lysine oxidase, a key enzyme involved in amino acid metabolism and cellular homeostasis that contributes to fungal growth and survival, revealed a wide range of predicted binding affinities (Table S8). Inhibition of this enzyme may disrupt essential metabolic processes, providing a plausible mechanism for the observed antifungal activity. Docking scores ranged from the weakest interaction with jaslanceoside B (4.19 kcal mol^−1^) to the strongest with pinellic acid (−112.88 kcal mol^−1^), identifying pinellic acid as the most promising inhibitor candidate. Malyngic acid (−107.79 kcal mol^−1^) and catechin (−99.10 kcal mol^−1^) also exhibited predicted binding, whereas jaslanceoside B and rutin (−47.19 kcal mol^−1^) showed poor affinity, likely due to insufficient structural complementarity. The 2D interaction diagrams (Fig. S10) indicate that binding is largely mediated by hydrogen bonds and π-interactions. All five representative molecules (catechin, isorhamnetin, malyngic acid, pinellic acid, and quercetin) engage the positively charged Arg45 residue, as well as polar residues such as Asn431, for stabilization. Aromatic molecules, including catechin (Fig. S10a), isorhamnetin (Fig. S10b), and quercetin (Fig. S10e), additionally exploit Pi–alkyl interactions with hydrophobic residues like Val14 and Leu46, further enhancing their binding within the binding site.

While the *in silico* study identified good molecular targets, these findings represent only one possible mechanism underlying the observed antifungal activity. The antimicrobial activity of the crude extracts is likely a consequence of the complex interactions among the numerous compounds identified in the extract, including synergistic effects and the involvement of additional cellular targets and mechanisms of action. Thus, we suggested that future isolation studies should be performed to understand which compounds are responsible for the observed antimicrobial abilities or their interactions, including synergetic or antagonistic effects.

### Molecular dynamics (MD) simulations and free energy calculations

RMSD profiles from 100 ns MD simulations of *A. fumigatus* thioredoxin reductase complexes (Fig. S11a) show that most complexes reach equilibrium after approximately 40 ns, with protein–jaslanceoside B and protein–quercetin complexes showing stable fluctuations in the 0.3–0.5 nm range for the remainder of the simulation. In contrast, the protein–pinellic acid complex displays higher deviations and greater fluctuation, suggesting a less stable binding mode or more pronounced conformational rearrangements. The RMSF values (Fig. S11b) remain consistent across complexes, with higher fluctuations restricted to loop and terminal regions, indicating that ligand binding does not significantly alter the flexibility of the protein. Similarly, the radius of gyration trends (Fig. S11c) show that most complexes maintain a compact structure around 2.25 nm. Protein–jaslanceoside B complex, however, exhibits the largest Rg variability, pointing to transient expansion events. H-bond analysis (Fig. S11d) helps explain these stability patterns: stable systems such as protein–malyngic acid and protein–pinellic acid sustain 5–10 hydrogen bonds throughout the simulation, whereas the weaker protein–jaslanceoside B complex frequently drops to very low H-bond counts. Consistent with these observations, the MM/PBSA results (Table S9) identify malyngic acid (−50.02 kcal mol^−1^) and pinellic acid (−50.61 kcal mol^−1^) as the strongest binders, while jaslanceoside B (−30.19 kcal mol^−1^) shows the weakest overall affinity. RMSD profiles from 100 ns MD simulations for *A. niger* endoglucanase A complexes (Fig. S12a) show that most complexes, especially protein–isoquercitrin and protein–pinellic acid complexes, rapidly stabilize and maintain values in a narrow range of 0.15–0.20 nm, suggesting rather stable binding conformations. In contrast, the protein–rutin complex displays the largest deviations after 40 ns, suggesting a more transient interaction. RMSF profiles (Fig. S12b) indicate comparable residue-level flexibility across all complexes, confirming that the overall protein fold remains intact regardless of the ligand. The radius of gyration trends (Fig. S12c) further support this stability, with most complexes maintaining a compact structure around 1.67 nm. A notable exception is the protein–jaslanceoside B complex, which exhibits a sharp Rg increase to ∼1.82 nm around 75 ns, implying a temporary loss of compactness or partial opening of the binding site. MM/PBSA results (Table S10) align closely with these observations: rutin (−50.61 kcal mol^−1^) emerges as the strongest predicted binder, followed by isoquercitrin (−43.42 kcal mol^−1^), while jaslanceoside B (−27.74 kcal mol^−1^) shows the weakest affinity. The 100 ns MD simulations of *B. cereus* sphingomyelinase C complexes highlight clear trends in structural stability and binding behavior (Fig. S13). RMSD profiles (Fig. S13a) show that all complexes rapidly reach equilibrium and remain highly stable within the narrow 0.10–0.15 nm range. Consistently, the RMSF values (Fig. S13b) reveal nearly identical residue-level flexibility across complexes, indicating that ligand binding does not induce major local structural perturbations. The radius of gyration plot (Fig. S13c) shows that most complexes maintain a compact fold, although protein–pinellic acid and protein–azelaic acid exhibit noticeably larger fluctuations, suggesting transient loosening of the protein structure. Hydrogen-bond analysis (Fig. S13d) further explains these differences: the most stable complexes, such as those with icariside B5 and malyngic acid, sustain 3–5 intermolecular H-bonds throughout the simulation, whereas the less stable pinellic acid and azelaic acid complexes maintain fewer and more sporadic H-bonds. This indicates that persistent hydrogen bonding is a key determinant of the overall structural stability. The MM/PBSA results (Table S11) align with the MD data, identifying icariside B5 (−30.39 kcal mol^−1^) and malyngic acid (−26.98 kcal mol^−1^) as the strongest predicted binders, while azelaic acid (−2.65 kcal mol^−1^) exhibits extremely weak binding affinity. The 100 ns MD simulations of the *E. coli* DNA gyrase complexes reveal a clear link between ligand stability and the persistence of intermolecular hydrogen bonds (Fig. S14). RMSD analysis (Fig. S14a) shows that all systems equilibrate within the first 20 ns and remain stable thereafter, fluctuating between 0.4 and 0.6 nm. The RMSF profiles (Fig. S14b) indicate largely conserved residue flexibility across the complexes, with the exception of a pronounced fluctuation near residue 400 in the protein–pinellic acid system. The radius of gyration results (Fig. S14c) similarly show no major structural expansion, confirming the preservation of the protein's overall compactness. Notably, the H-bond analysis (Fig. S14d) distinguishes stable from unstable complexes: protein–pinellic acid and protein–rutin consistently maintain 5–9 hydrogen bonds, whereas the protein–isoquercitrin complex forms fewer and more irregular H-bonds, directly correlating with its higher RMSD and greater Rg. MM/PBSA calculations (Table S12) support the MD observations, identifying pinellic acid (−42.58 kcal mol^−1^) and malyngic acid (−40.15 kcal mol^−1^) as the strongest binders, while isoquercitrin (−29.49 kcal mol^−1^) exhibits the weakest predicted affinity. The 100 ns MD simulations of the *L. monocytogenes* listeriolysin O complexes highlight the highly dynamic nature of ligand binding to this large pore-forming toxin (Fig. S15). RMSD data (Fig. S15a) show that most complexes—including protein–rutin—rapidly reach equilibrium and remain relatively stable between 0.2 and 0.6 nm, whereas protein–astragalin and protein–isoquercitrin exhibit reduced stability after ∼60 ns. RMSF profiles (Fig. S15b) are broadly similar across all systems, indicating that ligand binding does not substantially alter residue-level flexibility, with the most mobile regions corresponding to flexible loops or domain interfaces. The radius of gyration plot (Fig. S15c) reveals pronounced fluctuations across all complexes; however, overall compactness is maintained around 3.6 nm, suggesting that despite large internal motions, the global fold remains intact. The hydrogen-bond analysis (Fig. S15d) shows that all complexes form highly transient and intermittent H-bonds, frequently dropping to zero throughout the simulation. Such behavior is characteristic of inhibitors interacting with flexible toxins like listeriolysin O, where continuous hydrogen bonding is difficult to maintain and ligands may shift between adjacent sub-pockets. Consistent with the MD observations, MM/PBSA results (Table S13) identify pinellic acid (−26.40 kcal mol^−1^) and rutin (−21.80 kcal mol^−1^) as the strongest binders, while malyngic acid (−7.72 kcal mol^−1^) shows the weakest predicted affinity. The RMSD results from 100 ns MD simulations for *P. aeruginosa* arginine rhamnosyltransferase complexes (Fig. S16a) show that all complexes rapidly equilibrate and maintain stable orbitals in the range of 0.15–0.25 nm. RMSF profiles (Fig. S16b) further confirm that ligand binding does not significantly alter the protein's intrinsic flexibility, as all systems display comparable residue-level fluctuations. The radius of gyration plot (Fig. S16c) indicates that each complex preserves a compact global structure, fluctuating consistently around 2.25 nm throughout the simulation. Hydrogen bond analysis (Fig. S16d) reveals limited and highly spaced H-bond formation in several complexes, particularly those with low RMSD stability, such as dihydroferulic acid and azelaic acid. H-bond numbers frequently drop to zero, suggesting weak or transient interactions at the binding site. The MM/PBSA data (Table S14) closely agree with these observations: pinellic acid (−28.02 kcal mol^−1^) and icariside B5 (−26.78 kcal mol^−1^) exhibited the relatively strongest predicted binding affinities, while dihydroferulic acid (−11.70 kcal mol^−1^) was identified as the weakest binder.

MD simulation data for the *P. cyclopium* mono-/diacylglycerol lipase complexes highlight a link between hydrogen-bond persistence and overall complex stability (Fig. S17). The RMSD plot (Fig. S17a) shows that most complexes (protein–malyngic acid and protein–luteolin) rapidly reach equilibrium and remain tightly stabilized within the 0.125–0.175 nm range. The RMSF profiles (Fig. S17b) further indicate that ligand binding does not introduce notable local flexibility changes, confirming the preservation of the enzyme's structural integrity. Although the radius of gyration values are generally consistent across complexes (Fig. S17c), a slight loss of compactness is observed around 85 ns in the protein–icariside B5 system. Hydrogen-bond analysis (Fig. S17d) reinforces these trends: luteolin and pinellic acid maintain a steady network of 4–6 H-bonds, whereas the protein–eriodictyol complex shows sporadic and often absent H-bonding. Consistent with these observations, the MM/PBSA results (Table S9) rank malyngic acid (−34.05 kcal mol^−1^) and pinellic acid (−30.56 kcal mol^−1^) as the most favorable binders, while eriodictyol (−16.39 kcal mol^−1^) and icariside B5 (−16.66 kcal mol^−1^) exhibit the weakest binding affinities.

The RMSD profiles obtained from 100 ns MD simulations for *P. funiculosum* endo-1,4-β-xylanase C complexes (Fig. S18a) show that complexes such as protein–epicatechin and protein–eriodictyol rapidly equilibrate and remain stable in the range of 0.15–0.20 nm, while the protein–malyngic acid complex shows significantly higher RMSD values reaching ∼0.3 nm towards the end of the simulation. RMSF analysis (Fig. S18b) reveals generally similar residue flexibility across complexes, with a localized increase around residue 165 in the protein–catechin and protein–malyngic acid systems. The radius of gyration data (Fig. S18c) indicate a late-stage loss of compactness for the malyngic acid complex, further supporting its destabilized conformation. Hydrogen-bond profiles (Fig. S18d) show that pinellic acid and eriodictyol maintain stable networks of 3–5 H-bonds throughout the trajectory. The MM/PBSA results (Table S16) align with these observations, identifying pinellic acid (−34.45 kcal mol^−1^) and epicatechin (−24.45 kcal mol^−1^) as the strongest binders, while malyngic acid (−14.64 kcal mol^−1^) exhibits the weakest binding affinity. MD simulations of the *S. aureus* tyrosyl-tRNA synthetase complexes over 100 ns provide a picture of the overall stability and interaction profiles of the protein–ligand systems (Fig. S19). The RMSD plot (Fig. S19a) shows that most complexes rapidly reach equilibrium and maintain stable conformations within the 0.20–0.30 nm range. RMSF analysis (Fig. S19b) reveals highly consistent residue-level flexibility across all systems, indicating that ligand binding does not disrupt the protein's dynamic architecture. The radius of gyration values (Fig. S19c) further support this stability, showing preserved compactness throughout the simulations. Hydrogen-bond analysis (Fig. S19d) highlights that complexes with rutin and hyperoside sustain moderate to high H-bond numbers (4–8), while the relatively unstable protein–pinellic acid complex displays sparse and low H-bond counts. The MM/PBSA results (Table S11) identify rutin (−62.97 kcal mol^−1^) as the strongest binder, followed by malyngic acid (−51.45 kcal mol^−1^) and icariside B5 (−49.22 kcal mol^−1^), whereas pinellic acid (−46.43 kcal mol^−1^) shows the weakest binding affinity. The RMSD plot from 100 ns MD simulations of *T. viride*l-lysine oxidase complexes (Fig. S20a) shows that most complexes reach equilibrium within ∼15 ns and remain stable between 0.20 and 0.25 nm, indicating that ligand binding largely preserves the structural integrity of the enzyme. RMSF profiles (Fig. S20b) display similar residue-level fluctuations across all complexes, confirming that the overall fold remains intact. The radius of gyration values (Fig. S20c) further support this, with all systems maintaining comparable compactness (2.42–2.47 nm). Hydrogen-bond analysis (Fig. S20d) highlights that complexes such as malyngic acid and pinellic acid maintain consistently higher H-bond numbers (4–8), whereas the protein–isorhamnetin complex exhibits the lowest H-bond counts. The MM/PBSA results align with these observations, with ΔTOTAL binding energies identifying malyngic acid (−57.64 kcal mol^−1^) and pinellic acid (−51.16 kcal mol^−1^) as the strongest binders, while quercetin (−41.09 kcal mol^−1^) shows the weakest affinity (Table S18).

## Conclusion

The current study focuses on chemical constituent profiling of *S. pumila* and *S. confusa* using HPLC-ESI-Orbitrap-MS/MS analysis, and the bioactivity of the extracts was determined by several *in vitro* assays. In summary, the results revealed that the antioxidant activity of the extract correlates with its phenolic content, suggesting that *S. pumila* and *S. confusa*, being rich in phenolics, could serve as a valuable source of antioxidants. The highest phenolic and flavonoid contents were found in MeOH and water extracts. HPLC-ESI-Orbitrap-MS/MS revealed chemical diversity of the different extracts. HPLC-ESI-Orbitrap-MS/MS analysis performed here was qualitative, so we could not determine the relative abundance of the identified metabolites. As a result, the biological effects reported in this study cannot be linked to individual compounds on the basis of concentration. The proposed contributions of selected metabolites are therefore based on their identification and *in silico* binding affinity, not on quantitative evidence. Establishing a clear relationship between metabolite abundance and activity will require future work using targeted or semi-quantitative LC-MS methods. Both plant MeOH and water extracts possessed the most potent antioxidant capacity in most of the tested assays. The *S. pumila* water extracts, while *S. confusa* MeOH extracts, significantly showed the highest anti-AChE and anti-tyrosinase activity. All the tested extracts of *S. pumila* showed lower inhibition of α-amylase and α-glucosidase, while *S. confusa* extracts showed no anti-α-glucosidase activity. On the other hand, the EA extract of *S. pumila* exhibited the highest anti-BChE activity. The different antimicrobial profiles of *S. pumila* and *S. confusa* extracts, particularly the methanolic extracts, show strong bactericidal action against Gram-positive strains and broad-spectrum antifungal activities. The findings highlight that all extracts exhibit notable antioxidant activity, as well as promising neuroprotective and dermoprotective effects, supporting their potential for further exploration in future studies.

## Author contributions

Conceptualization: NN, ECB, JG, DS, CZ, GZ; methodology: ECB, SK, JG, JJ, CZ, NT, GZ; software: NN, ECB, SK; validation: JG, GZ; formal analysis: GZ, EY; investigation: NN, ECB, SK, NP, DS, EY, NT, GZ; resources: EY; data curation: NT, GZ; writing – original draft preparation: NN, ECB, JG, DS, GZ; writing – review and editing: NT, EY, GZ; visualization: ECB, GC; supervision: NT, GZ; project administration: GZ; and funding acquisition: GZ. All authors have read and agreed to the published version of the manuscript.

## Conflicts of interest

The authors declare no conflicts of interest.

## Supplementary Material

RA-016-D6RA06442F-s001

## Data Availability

Data will be made available from the authors upon request. Supplementary information (SI) is available. See DOI: https://doi.org/10.1039/d6ra06442f.
