# Supplementary material for: Metabolomic characterization and multi-target biological activities of Saponaria pumila and Suaeda confusa: integrating in vitro and in silico approaches
Source: RSC Adv. 2026 Sep 29;16(45):58329–45. doi: 10.1039/d6ra06442f (PMC13621350; doi:10.1039/d6ra06442f)
Supplement: Supplementary file 1 [file RA-016-D6RA06442F-s001.pdf]

Supplementary Information (SI) for RSC Advances.
This journal is © The Royal Society of Chemistry

Phytochemical analysis by HPLC-ESI-Orbitrap-MS/MS

Plant extracts are complex mixtures containing hundreds of different organic compounds. Phytochemical analysis by HPLC-ESI-Orbitrap-MS/MS (Ultra High-Performance Liquid Chromatography coupled with Electrospray Ionization Mass Spectrometry) is one of the best method for untargeted analysis, profiling and identifying plant secondary metabolites. UHPLC-ESI-MS has high detection selectivity, high sensitivity and high qualitative productivity. ESI source is most widely used as an ion source for polar and ionic biomolecules. ESI can operate in both positive and negative ion modes Small molecules (<500 Da) with a single functional group predominantly give singly charged ions. Positive ion mode is the best for basic, polar and amine-rich compounds. There is the addition of a proton to the analyte ($M+H^+$). Negative ion mode is the best for acidic compounds, eg phenolics. There is a loss of a proton ($M-H^−$) in negative ion mode. Adduct cations (e.g. $M+NH_4^+$, $M+Na^+$, $M+K^+$) and anions (e.g. M+formate⁻, M+acetate⁻) can occur when salts are present.

The liquid chromatographic system was Dionex 3000RS Ultimate system where separations were achieved on a Thermo Accucore C18 (100 mm x 2.1, mm i. d., 2.6 μm) column themostated at 25 °C (± 1 °C). The solvents used were water (A) and methanol (B). Both was acidified with 0.1 % formic acid. The flow rate was maintained at 0.2 mL min^{-1}. The analysis was performed using the following gradient elution: isocratic 5 % B (0-3 min), a linear gradient increasing from 5% B to 100% (3-43 min), 100% B (43-61 min), a linear gradient decreasing from 100% B to 5% (61-62 min) and 5 % B (62-70 min). The column was coupled with a Thermo Q-Exactive Orbitrap mass spectrometer (Thermo Scientific, USA) equipped with electrospray ionization source. The total run time of analysis was 70 minutes. Samples were analyzed in duplicates, in both positive (ESI+) and negative ionization modes (ESI-). All HPLC–ESI-Orbitrap-MS/MS data acquisitions were performed and monitored using Xcalibur 3.0.63 software (Thermo Scientific Inc, Waltham, MA, USA). All data were processed using Thermo TraceFinder software and tentatively identified by comparing their data (retention time, fragmentation pattern) with our spectral library and online databases (Massbank of North America, m/z Cloud). The difference between the measured and calculated monoisotopic molecular masses was less than 5 ppm in every case.

Table S1. Chemical composition of the ethyl acetate extract of *Suaeda confusa*

No.	Name	Formula	Rt	[M + H]+	[M - H]-	Theoretical m/z	|Δppm|	Fragment 1	Fragment 2	Fragment 3	Fragment 4	Fragment 5	Literature
1	Trigonelline	C7H7NO2	2,01	138.05550[2]		138.05495	3.96	110,0604	96,0445	94,0656	92,0498		
2[1]	Caffeic acid	C9H8O4	17,51		179,03444	179.03498	3.03	135,0439	107,0481				
3[1]	Vanillin (4-Hydroxy-3-methoxybenzaldehyde)	C8H8O3	18,31	153,05517		153.05462	3.59	125,0598	111,0443	110,0366	93,0339	65,0392	
4	Tomenin (Tomentin-5-O-glucoside)	C17H20O10	19,97	385,11348		385.11292	1.45	223,0599	208,0364	190,0259	162,0310		
5	12-Hydroxyjasmonic acid or Tuberonic acid	C12H18O4	20,11		225,11269	225.11323	2.41	165,0907	161,1251	147,0800	59,0123		
6[1]	p-Coumaric acid	C9H8O3	20,67		163,03952	163.04007	3.36	119,0488					
7	N-cis-Feruloyloctopamine	C18H19NO5	20,90		328,11850	328.11905	1.66	310,1086	295,0848	161,0231	149,0595	133,0520	
8	Indole-3-carbaldehyde	C9H7NO	21,41	146,06059		146.06004	3.76	118,0653	117,0578	91,0546			
9	Indole-3-carboxylic acid	C9H7NO2	21,77	162,05551		162.05495	3.43	144,0443	118,0653	116,0499	91,0546		
10[1]	Ferulic acid	C10H10O4	21,84		193,05009	193.05063	2.81	178,0261	149,0595	137,0231	134,0360	121,0281	
11	N-trans-Feruloyloctopamine	C18H19NO5	23,45		328,11850	328.11905	1.66	310,1086	295,0849	161,0231	149,0597	133,0519	
12	Tomentin (6,7-Dimethoxy-5-hydroxycoumarin)	C11H10O5	24,16	223,06065		223.06010	2.47	208,0366	190,0261	162,0311	135,0438		
13	6,7-Dihydroxycoumarin-3-carboxylic acid	C10H6O6	24,42		221,00862	221.00916	2.45	177,0183	151,0021	135,0074	133,0282	89,0382	
14	N-cis-Feruloyltyramine	C18H19NO4	24,44	314,13924		314.13868	1.77	177,0546	149,0595	145,0284	121,0650	117,0337	
15	N-trans-Caffeoyltyramine	C17H17NO4	24,54	300,12359		300.12303	1.85	163,0388	145,0284	135,0441	121,0650	93,0702	
16	Isorhamnetin-3-O-robinobioside	C28H32O16	24,59		623,16122	623.16176	0.86	315,0513	314,0432	300,0279	299,0197	271,0248	
17[1]	Hyperoside (Quercetin-3-O-galactoside)	C21H20O12	25,06		463,08765	463.08820	1.19	301,0346	300,0265	271,0241	255,0280	151,0020	
18[1]	Isoquercitrin (Quercetin-3-O-glucoside)	C21H20O12	25,24		463,08765	463.08820	1.19	301,0355	300,0278	271,0247	255,0278	151,0022	
19[1]	Rutin (Quercetin-3-O-rutinoside)	C27H30O16	25,30		609,14557	609.14611	0.88	301,0374	300,0273	271,0245	255,0297	151,0023	
20	Jaslanceoside B	C26H30O14	25,89		565,15574	565.15628	0.95	221,0449	177,0547	163,0388	145,0282	119,0487	

21	N-trans-Feruloyltyramine	$C_{18}H_{19}NO_4$	26,82	314,13924		314.13868	1.77	177,0546	149,0595	145,0284	121,0650	117,0337	
22	Abscisic acid	$C_{15}H_{20}O_4$	27,11		263,12834	263.12888	2.06	219,1382	204,1145	201,1278	152,0830	151,0751	
23[1]	Isorhamnetin-3-O-glucoside	$C_{22}H_{22}O_{12}$	27,15		477,10330	477.10385	1.15	315,0506	314,0435	299,0199	285,0405	243,0294	
24	Isorhamnetin-3-O-rutinoside (Narcissin)	$C_{28}H_{32}O_{16}$	27,43		623,16122	623.16176	0.86	315,0509	314,0437	300,0281	299,0197	271,0251	
25[1]	Naringenin (4',5,7-Trihydroxyflavanone)	$C_{15}H_{12}O_5$	29,18		271,06065	271.06120	2.02	177,0181	151,0023	119,0487	107,0124		
26	Homoeriodictyol (3'-Methoxy-4',5,7-trihydroxyflavanone)	$C_{16}H_{14}O_6$	29,22		301,07122	301.07176	1.80	151,0023	149,0592	134,0363	83,0121		
27[1]	Quercetin (3,3',4',5,7-Pentahydroxyflavone)	$C_{15}H_{10}O_7$	29,32		301,03483	301.03538	1.81	178,9977	151,0023	121,0281	107,0123		
28[1]	Luteolin (3',4',5,7-Tetrahydroxyflavone)	$C_{15}H_{10}O_6$	30,27		285,03991	285.04046	1.94	175,0388	151,0018	133,0279			
29[1]	Isorhamnetin (3'-Methoxy-3,4',5,7-tetrahydroxyflavone)	$C_{16}H_{12}O_7$	31,98		315,05048	315.05103	1.73	300,0276	283,0256	271,0248	227,0346	151,0024	
30[1]	Chrysoeriol (3'-Methoxy-4',5,7-trihydroxyflavone)	$C_{16}H_{12}O_6$	32,24		299,05556	299.05611	1.84	284,0327	256,0375	255,0288	227,0350		
31	Traumatic acid (2-Dodecenedioic acid)	$C_{12}H_{20}O_4$	33,41		227,12834	227.12888	2.39	209,1171	183,1382	165,1275	111,0801		
32	Traumatic acid isomer	$C_{12}H_{20}O_4$	33,87		227,12834	227.12888	2.39	209,1175	183,1382	165,1273	111,0799		
33[1]	α-Linolenic acid	$C_{18}H_{30}O_2$	45,21		277,21676	277.21730	1.96	259,2064	233,2270	71,0120	59,0122		
34[1]	Linoleic acid	$C_{18}H_{32}O_2$	46,14		279,23241	279.23295	1.95						
35[1]	Palmitic acid	$C_{16}H_{32}O_2$	46,77		255,23241	255.23295	2.13						
36[1]	Oleic acid	$C_{18}H_{34}O_2$	47,09		281,24806	281.24860	1.93						

[1] Confirmed by standard

[2] $[M]^+$

Table S2. Chemical composition of the methanol extract of *Suaeda confusa*

No.	Name	Formula	Rt	[M + H]+	[M - H]-	Theoretical m/z	|Δppm|	Fragment 1	Fragment 2	Fragment 3	Fragment 4	Fragment 5	Literature
1	Trigonelline	C7H7NO2	1,99	138.05550[2]		138.05495	3.96	110,0604	96,0447	94,0656	92,0496		
2	Citric acid	C6H8O7	3,02		191,01918	191.01973	2.86	173,0082	129,0181	111,0072	87,0071	85,0279	
3	Uralenneoside	C12H14O8	14,71		285,06105	285.06159	1.90	153,0180	152,0103	109,0280	108,0202		
4[1]	Caffeic acid	C9H8O4	17,52		179,03444	179.03498	3.03	135,0439	107,0483				
5[1]	Vanillin (4-Hydroxy-3-methoxybenzaldehyde)	C8H8O3	18,34	153,05517		153.05462	3.59	125,0598	111,0443	110,0363	93,0339	65,0392	
6	Kynurenic acid	C10H7NO3	18,64	190,05042		190.04987	2.90	162,0549	144,0434	116,0496	89,0389		
7	Tomenin (Tomentin-5-O-glucoside)	C17H20O10	20,00	385,11348		385.11292	1.45	223,0600	208,0372	190,0264	162,0309		
8	12-Hydroxyjasmonic acid or Tuberonic acid	C12H18O4	20,14		225,11269	225.11323	2.41	165,0909	161,1251	147,0802	59,0123		
9[1]	p-Coumaric acid	C9H8O3	20,70		163,03952	163.04007	3.36	119,0488					
10	N-cis-Feruloyloctopamine	C18H19NO5	20,93		328,11850	328.11905	1.66	310,1089	295,0849	161,0233	149,0592	133,0521	
11[1]	Ferulic acid	C10H10O4	21,84		193,05009	193.05063	2.81	178,0262	149,0595	137,0231	134,0360	121,0282	
12	p-Coumaroylmalic acid	C13H12O7	22,83		279,05048	279.05103	1.96	163,0388	133,0130	119,0487	115,0022	71,0123	
13	Isorhamnetin-di-O-hexoside isomer 1	C28H32O17	22,97		639,15613	639.15667	0.85	477,1049	476,0951	315,0516	313,0356	285,0410	
14	Isorhamnetin-di-O-hexoside isomer 2	C28H32O17	23,20		639,15613	639.15667	0.85	477,1049	476,0967	315,0513	313,0357	285,0409	
15	N-trans-Feruloyloctopamine	C18H19NO5	23,45		328,11850	328.11905	1.66	310,1087	295,0851	161,0232	149,0594	133,0520	
16	Isorhamnetin-3-O-rutinoside-7-O-glucoside	C34H42O21	23,68		785,21404	785.21458	0.69	623,1615	315,0511	314,0436	300,0285	299,0201	
17	6,7-Dihydroxycoumarin-3-carboxylic acid	C10H6O6	24,42		221,00862	221.00916	2.45	177,0183	151,0023	135,0075	133,0282	89,0381	
18	N-cis-Feruloyltyramine	C18H19NO4	24,44	314,13924		314.13868	1.77	177,0547	149,0597	145,0286	121,0651	117,0335	
19	N-trans-Caffeoyltyramine	C17H17NO4	24,54	300,12359		300.12303	1.85	163,0390	145,0287	135,0442	121,0650	93,0702	
20	Isorhamnetin-3-O-robinobioside	C28H32O16	24,59		623,16122	623.16176	0.86	315,0511	314,0433	300,0278	299,0197	271,0248	

21[1]	Hyperoside (Quercetin-3-O-galactoside)	$C_{21}H_{20}O_{12}$	25,06		463,08765	463.08820	1.19	301,0358	300,0282	271,0256	255,0282	151,0021	
22[1]	Isoquercitrin (Quercetin-3-O-glucoside)	$C_{21}H_{20}O_{12}$	25,24		463,08765	463.08820	1.19	301,0355	300,0278	271,0249	255,0298	151,0023	
23[1]	Rutin (Quercetin-3-O-rutinoside)	$C_{27}H_{30}O_{16}$	25,30		609,14557	609.14611	0.88	301,0356	300,0275	271,0251	255,0296	151,0025	
24	Jaslanceoside B	$C_{26}H_{30}O_{14}$	25,89		565,15574	565.15628	0.95	221,0452	177,0550	163,0388	145,0283	119,0487	
25	Quercetin-3-O-(6″-malonyl)glucoside	$C_{24}H_{22}O_{15}$	26,39		549,08805	549.08859	0.99	505,1029	301,0350	300,0282	271,0251		
26	N-trans-Feruloyltyramine	$C_{18}H_{19}NO_4$	26,83	314,13924		314.13868	1.77	177,0546	149,0598	145,0285	121,0650	117,0338	
27[1]	Isorhamnetin-3-O-glucoside	$C_{22}H_{22}O_{12}$	27,16		477,10330	477.10385	1.15	315,0511	314,0434	299,0196	285,0406	243,0295	1
28	Isorhamnetin-3-O-rutinoside (Narcissin)	$C_{28}H_{32}O_{16}$	27,43		623,16122	623.16176	0.86	315,0512	314,0435	300,0276	299,0196	271,0249	
29[1]	Naringenin (4′,5,7-Trihydroxyflavanone)	$C_{15}H_{12}O_5$	29,18		271,06065	271.06120	2.02	177,0186	151,0025	119,0488	107,0124		
30	Homoeriodictyol (3′-Methoxy-4′,5,7-trihydroxyflavanone)	$C_{16}H_{14}O_6$	29,21		301,07122	301.07176	1.80	151,0024	149,0595	134,0359	83,0123		
31[1]	Quercetin (3,3′,4′,5,7-Pentahydroxyflavone)	$C_{15}H_{10}O_7$	29,34		301,03483	301.03538	1.81	178,9973	151,0021	121,0284	107,0125		
32[1]	Luteolin (3′,4′,5,7-Tetrahydroxyflavone)	$C_{15}H_{10}O_6$	30,26		285,03991	285.04046	1.94	175,0390	151,0025	133,0278			
33[1]	Isorhamnetin (3′-Methoxy-3,4′,5,7-tetrahydroxyflavone)	$C_{16}H_{12}O_7$	31,98		315,05048	315.05103	1.73	300,0277	283,0260	271,0249	227,0348	151,0023	1
34[1]	Chrysoeriol (3′-Methoxy-4′,5,7-trihydroxyflavone)	$C_{16}H_{12}O_6$	32,25		299,05556	299.05611	1.84	284,0333	256,0369	255,0286	227,0349		
35	Traumatic acid (2-Dodecenedioic acid)	$C_{12}H_{20}O_4$	33,41		227,12834	227.12888	2.39	209,1182	183,1382	165,1274	111,0801		
36	Traumatic acid isomer	$C_{12}H_{20}O_4$	33,88		227,12834	227.12888	2.39	209,1172	183,1382	165,1273	111,0797		

[1] Confirmed by standard

[2] [M]+

Table S3. Chemical composition of the water extract of *Suaeda confusa*

No.	Name	Formula	Rt	[M + H]+	[M - H]-	Theoretical m/z	|Δppm|	Fragment 1	Fragment 2	Fragment 3	Fragment 4	Fragment 5	Literature
1	Trigonelline	$C_7H_7NO_2$	2,01	138.05550[2]		138.05495	3.96	110,0604	96,0445	94,0655	92,0498		
2	Citric acid	$C_6H_8O_7$	2,96		191,01918	191.01973	2.86	173,0078	129,0181	111,0072	87,0071	85,0279	
3	Uralenneoside	$C_{12}H_{14}O_8$	14,66		285,06105	285.06159	1.90	153,0173	152,0101	109,0278	108,0201		
4[1]	Caffeic acid	$C_9H_8O_4$	17,48		179,03444	179.03498	3.03	135,0437	107,0482				
5	Kynurenic acid	$C_{10}H_7NO_3$	18,65	190,05042		190.04987	2.90	162,0549	144,0433	116,0494	89,0387		
6	12-Hydroxyjasmonic acid or Tuberonic acid	$C_{12}H_{18}O_4$	20,12		225,11269	225.11323	2.41	165,0910	161,1253	147,0809	59,0123		
7[1]	p-Coumaric acid	$C_9H_8O_3$	20,67		163,03952	163.04007	3.36	119,0487					
8	N-cis-Feruloyloctopamine	$C_{18}H_{19}NO_5$	20,91		328,11850	328.11905	1.66	310,1086	295,0855	161,0229	149,0590	133,0520	
9[1]	Ferulic acid	$C_{10}H_{10}O_4$	21,81		193,05009	193.05063	2.81	178,0262	149,0599	137,0231	134,0361	121,0280	
10	p-Coumaroylmalic acid	$C_{13}H_{12}O_7$	22,82		279,05048	279.05103	1.96	163,0388	133,0127	119,0489	115,0022	71,0122	
11	Isorhamnetin-di-O-hexoside isomer 1	$C_{28}H_{32}O_{17}$	22,95		639,15613	639.15667	0.85	477,1053	476,0985	315,0517	313,0359	285,0412	
12	Isorhamnetin-di-O-hexoside isomer 2	$C_{28}H_{32}O_{17}$	23,18		639,15613	639.15667	0.85	477,1044	476,0935	315,0515	313,0359	285,0395	
13	N-trans-Feruloyloctopamine	$C_{18}H_{19}NO_5$	23,44		328,11850	328.11905	1.66	310,1086	295,0847	161,0231	149,0593	133,0521	
14	Isorhamnetin-3-O-rutinoside-7-O-glucoside	$C_{34}H_{42}O_{21}$	23,66		785,21404	785.21458	0.69	623,1619	315,0516	314,0434	300,0297	299,0196	
15	Tomentin (6,7-Dimethoxy-5-hydroxycoumarin)	$C_{11}H_{10}O_5$	24,14	223,06065		223.06010	2.47	208,0363	190,0258	162,0313	135,0443		
16	6,7-Dihydroxycoumarin-3-carboxylic acid	$C_{10}H_6O_6$	24,45		221,00862	221.00916	2.45	177,0184	151,0021	135,0074	133,0280	89,0382	
17	Isorhamnetin-3-O-robinobioside	$C_{28}H_{32}O_{16}$	24,57		623,16122	623.16176	0.86	315,0515	314,0434	300,0273	299,0197	271,0251	
18[1]	Hyperoside (Quercetin-3-O-galactoside)	$C_{21}H_{20}O_{12}$	25,04		463,08765	463.08820	1.19	301,0356	300,0280	271,0255	255,0281	151,0021	

19[1]	Isoquercitrin (Quercetin-3-O-glucoside)	$C_{21}H_{20}O_{12}$	25,23		463,08765	463.08820	1.19	301,0352	300,0273	271,0258	255,0299	151,0023	
20[1]	Rutin (Quercetin-3-O-rutinoside)	$C_{27}H_{30}O_{16}$	25,29		609,14557	609.14611	0.88	301,0349	300,0283	271,0251	255,0290	178,9979	
21	Jaslanceoside B	$C_{26}H_{30}O_{14}$	25,86		565,15574	565.15628	0.95	221,0440	177,0549	163,0385	145,0283	119,0485	
22	N-trans-Feruloyltyramine	$C_{18}H_{19}NO_4$	26,82	314,13924		314.13868	1.77	177,0546	149,0594	145,0284	121,0650	117,0337	
23[1]	Isorhamnetin-3-O-glucoside	$C_{22}H_{22}O_{12}$	27,14		477,10330	477.10385	1.15	315,0508	314,0434	299,0204	285,0403	243,0294	[1]
24	Isorhamnetin-3-O-rutinoside (Narcissin)	$C_{28}H_{32}O_{16}$	27,42		623,16122	623.16176	0.86	315,0513	314,0435	300,0275	299,0199	271,0245	
25[1]	Naringenin (4',5,7-Trihydroxyflavanone)	$C_{15}H_{12}O_5$	29,17		271,06065	271.06120	2.02	177,0184	151,0022	119,0483	107,0121		

[1] Confirmed by standard

[2] [M]+

Table S4. Chemical composition of the ethyl acetate extract of *Saponaria pumila*

No.	Name	Formula	Rt	$[M + H]^+$	$[M - H]^-$	Theoretical m/z	|Δppm|	Fragment 1	Fragment 2	Fragment 3	Fragment 4	Fragment 5
1[1]	Catechin	$C_{15}H_{14}O_6$	15,90		289,07121	289.07176	1.91	245,0809	203,0705	151,0385	123,0437	109,0281
2[1]	Caffeic acid	$C_9H_8O_4$	17,48		179,03444	179.03498	3.03	135,0439	107,0488			
3[1]	Vanillin (4-Hydroxy-3-methoxybenzaldehyde)	$C_8H_8O_3$	18,33	153,05517		153.05462	3.59	125,0599	111,0444	110,0367	93,0340	65,0393
4	Licoagroside B or isomer	$C_{18}H_{24}O_{12}$	18,36		431,11896	431.11950	1.25	203,0558	161,0448	125,0231	99,0437	57,0330
5[1]	Epicatechin	$C_{15}H_{14}O_6$	19,08		289,07121	289.07176	1.91	245,0820	203,0709	151,0388	123,0436	109,0282
6	Icariside B5 or isomer	$C_{19}H_{32}O_8$	19,89	389,21755		389.21699	1.43	227,1640	209,1539	191,1435	173,1326	133,1013
7	Dihydroferulic acid (3-(4-Hydroxy-3-methoxyphenyl)propionic acid)	$C_{10}H_{12}O_4$	20,42		195,06568	195.06628	3.09	151,0388	136,0518	135,0440	121,0281	119,0488
8[1]	p-Coumaric acid	$C_9H_8O_3$	20,68		163,03952	163.04007	3.36	119,0488				
9	Abiesinol G (Vitisinol) or isomer	$C_{30}H_{22}O_{12}$	21,84		573,10331	573.10385	0.94	555,0940	529,1159	447,0725	325,0359	285,0408
10[1]	Ferulic acid	$C_{10}H_{10}O_4$	21,85		193,05009	193.05063	2.81	178,0263	149,0596	137,0232	134,0362	121,0280
11	Eriodictyol-O-hexoside	$C_{21}H_{22}O_{11}$	22,22		449,10839	449.10894	1.21	287,0564	151,0024	135,0439	107,0124	
12[1]	Sinapic acid	$C_{11}H_{12}O_5$	22,29		223,06065	223.06120	2.45	208,0369	193,0138	179,0709	164,0469	149,0227
13	O-Benzyl-O-(3-hydroxy-3-methylglutaroyl)-hexose	$C_{19}H_{26}O_{10}$	23,72		413,14477	413.14532	1.33	269,1034	161,0444	99,0438	59,0123	57,0330

14[1]	Hyperoside (Quercetin-3-O-galactoside)	$C_{21}H_{20}O_{12}$	25,26		463,08765	463.08820	1.19	301,0354	300,0276	271,0251	255,0296	151,0025
15[1]	Rutin (Quercetin-3-O-rutinoside)	$C_{27}H_{30}O_{16}$	25,31		609,14557	609.14611	0.88	301,0355	300,0277	271,0251	255,0298	151,0025
16[1]	Isoquercitrin (Quercetin-3-O-glucoside)	$C_{21}H_{20}O_{12}$	25,42		463,08765	463.08820	1.19	301,0359	300,0281	271,0251	255,0302	151,0025
17	Dimethoxycinnamic acid	$C_{11}H_{12}O_4$	25,61	209,08139		209.08084	2.65	191,0704	176,0471	163,0754	148,0519	105,0700
18[1]	Cosmosiin (Apigenin-7-O-glucoside)	$C_{21}H_{20}O_{10}$	26,24		431,09782	431.09837	1.28	269,0455	268,0386	151,0022	149,0225	
19	Azelaic acid (Nonanedioic acid)	$C_9H_{16}O_4$	26,32		187,09704	187.09758	2.90	169,0858	143,1066	125,0958	123,0802	97,0644
20	Chrysoeriol-7-O-glucoside	$C_{22}H_{22}O_{11}$	26,49		461,10839	461.10894	1.18	446,0870	299,0549	298,0480	283,0249	255,0297
21	O-p-Coumaroyl-O-hydroxybenzoyl-rhamnosylhexoside	$C_{28}H_{32}O_{14}$	26,75		591,17138	591.17193	0.93	445,1158	307,0828	163,0393	145,0285	137,0231
22[1]	Eriodictyol (3',4',5,7-Tetrahydroxyflavanone)	$C_{15}H_{12}O_6$	26,93		287,05556	287.05611	1.92	151,0028	135,0439	125,0230	107,0123	
23[1]	Astragalin (Kaempferol-3-O-glucoside)	$C_{21}H_{20}O_{11}$	27,00		447,09274	447.09329	1.22	285,0413	284,0328	255,0295	227,0336	151,0022
24	Kaempferol-3-O-rutinoside (Nicotiflorin)	$C_{27}H_{30}O_{15}$	27,12		593,15065	593.15119	0.92	327,0515	285,0407	284,0329	255,0299	227,0344
25	O-Feruloyl-O-hydroxybenzoyl-hexose	$C_{23}H_{24}O_{11}$	27,23		475,12404	475.12459	1.15	337,0932	193,0494	175,0391	160,0156	137,0232
26	Isorhamnetin-3-O-rutinoside (Narcissin)	$C_{28}H_{32}O_{16}$	27,47		623,16122	623.16176	0.86	315,0514	314,0435	300,0277	299,0200	271,0250
27[1]	Naringenin (4',5,7-Trihydroxyflavanone)	$C_{15}H_{12}O_5$	29,20		271,06065	271.06120	2.02	177,0184	165,0177	151,0025	119,0488	107,0124
28[1]	Quercetin (3,3',4',5,7-Pentahydroxyflavone)	$C_{15}H_{10}O_7$	29,33		301,03483	301.03538	1.81	273,0416	245,0450	178,9979	151,0025	121,0280
29[1]	Luteolin (3',4',5,7-Tetrahydroxyflavone)	$C_{15}H_{10}O_6$	30,28		285,03991	285.04046	1.94	217,0496	199,0392	175,0392	151,0025	133,0282
30[1]	Kaempferol (3,4',5,7-Tetrahydroxyflavone)	$C_{15}H_{10}O_6$	31,54		285,03991	285.04046	1.94	257,0466	229,0500	213,0552	151,0021	
31[1]	Isorhamnetin (3'-Methoxy-3,4',5,7-tetrahydroxyflavone)	$C_{16}H_{12}O_7$	32,01		315,05048	315.05103	1.73	300,0279	283,0244	271,0261	164,0103	151,0024
32[1]	Apigenin (4',5,7-Trihydroxyflavone)	$C_{15}H_{10}O_5$	32,04		269,04500	269.04555	2.03	227,0346	225,0555	151,0025	149,0231	117,0331
33	Methyl-trihydroxyxanthone	$C_{14}H_{10}O_5$	32,16		257,04500	257.04555	2.13	215,0345	213,0551			
34[1]	Chrysoeriol (3'-Methoxy-4',5,7-trihydroxyflavone)	$C_{16}H_{12}O_6$	32,28		299,05556	299.05611	1.84	284,0330	256,0377	227,0341	151,0026	107,0121
35	Malyngic acid (9,12,13-Trihydroxy-10E,15Z-octadecadienoic acid)	$C_{18}H_{32}O_5$	33,66		327,21715	327.21770	1.67	309,2070	291,1972	229,1441	211,1333	171,1016
36	Pinellic acid (9,12,13-Trihydroxy-10E-octadecenoic acid)	$C_{18}H_{34}O_5$	34,87		329,23280	329.23335	1.66	311,2226	293,2140	229,1442	211,1334	99,0801
37	Dihydroxy-methoxy(iso)flavone	$C_{16}H_{12}O_5$	35,62		283,06065	283.06120	1.93	268,0379	240,0427	151,0021		
38	Kumatakenin (4',5-Dihydroxy-3,7-dimethoxyflavone)	$C_{17}H_{14}O_6$	36,00		313,07122	313.07176	1.73	298,0485	297,0424	283,0252	270,0543	255,0297
39	Hydroxy-trimethoxy(iso)flavone	$C_{18}H_{16}O_6$	37,53	329,10251		329.10196	1.66	314,0783	313,0703	300,0631	299,0560	285,0754

40	Apigenin-4',7-dimethyl ether (4',7-Dimethoxy-5-hydroxyflavone)	$C_{17}H_{14}O_5$	39,21	299,09195		299.09140	1.84	284,0678	256,0727	167,0341		
41[1]	α-Linolenic acid	$C_{18}H_{30}O_2$	45,28		277,21676	277.21730	1.96	259,2075	59,0122			
42[1]	Palmitic acid	$C_{16}H_{32}O_2$	46,84		255,23241	255.23295	2.13					
43[1]	Oleic acid	$C_{18}H_{34}O_2$	47,16		281,24806	281.24860	1.93					

[1] Confirmed by standard

Table S5. Chemical composition of the methanol extract of *Saponaria pumila*

No.	Name	Formula	Rt	$[M + H]^+$	$[M - H]^-$	Theoretical m/z	|Δppm|	Fragment 1	Fragment 2	Fragment 3	Fragment 4	Fragment 5
1	Quinic acid	$C_7H_{12}O_6$	2,07		191,05557	191.05611	2.84	127,0389	111,0439	93,0330	85,0280	
2	Citric acid	$C_6H_8O_7$	2,99		191,01918	191.01973	2.86	173,0082	129,0181	111,0073	87,0072	85,0280
3	Catechin-O-hexoside	$C_{21}H_{24}O_{11}$	12,87		451,12404	451.12459	1.21	289,0721	245,0823	203,0714	151,0390	125,0232
4[1]	Catechin	$C_{15}H_{14}O_6$	15,88		289,07121	289.07176	1.91	245,0808	203,0702	151,0384	123,0437	109,0280
5	Epicatechin-O-hexoside	$C_{21}H_{24}O_{11}$	16,61		451,12404	451.12459	1.21	289,0723	245,0819	203,0695	151,0391	125,0232
6[1]	Caffeic acid	$C_9H_8O_4$	17,49		179,03444	179.03498	3.03	135,0439	107,0490			
7[1]	Vanillin (4-Hydroxy-3-methoxybenzaldehyde)	$C_8H_8O_3$	18,32	153,05517		153.05462	3.59	125,0599	111,0444	110,0366	93,0340	65,0393
8	Licoagroside B or isomer	$C_{18}H_{24}O_{12}$	18,39		431,11896	431.11950	1.25	203,0555	161,0445	125,0231	99,0437	57,0330
9	Kynurenic acid	$C_{10}H_7NO_3$	18,66	190,05042		190.04987	2.90	162,0549	144,0440	116,0497	89,0392	
10[1]	Epicatechin	$C_{15}H_{14}O_6$	19,09		289,07121	289.07176	1.91	245,0820	203,0705	151,0388	123,0436	109,0279
11	Icariside B5 or isomer	$C_{19}H_{32}O_8$	19,87	389,21755		389.21699	1.43	227,1643	209,1536	191,1429	173,1325	133,1014
12	Dihydroferulic acid (3-(4-Hydroxy-3-methoxyphenyl)propionic acid)	$C_{10}H_{12}O_4$	20,44		195,06568	195.06628	3.09	151,0391	136,0518	135,0440	121,0282	119,0488

13[1]	p-Coumaric acid	C9H8O3	20,67		163,03952	163.04007	3.36	119,0488					
14	Phaselic acid (2-O-Caffeoylmalic acid)	C13H12O8	20,70		295,04540	295.04594	1.83	179,0341	135,0439	133,0130	115,0026	71,0121	
15	Abiesinol G (Vitisinol) or isomer	C30H22O12	21,81		573,10331	573.10385	0.94	555,0944	529,1162	447,0725	325,0357	285,0408	
16[1]	Ferulic acid	C10H10O4	21,82		193,05009	193.05063	2.81	178,0263	149,0596	137,0232	134,0361	121,0281	
17	Eriodictyol-O-hexoside	C21H22O11	22,20		449,10839	449.10894	1.21	287,0556	151,0022	135,0443	107,0127		
18[1]	Sinapic acid	C11H12O5	22,29		223,06065	223.06120	2.45	208,0371	193,0136	179,0706	164,0468	149,0231	
19	Quercetin-O-dirhamnosylhexoside	C33H40O20	22,97		755,20347	755.20402	0.72	301,0367	300,0275	271,0245	255,0317		
20	O-Benzyl-O-(3-hydroxy-3-methylglutaroyl)-hexose	C19H26O10	23,73		413,14477	413.14532	1.33	269,1035	161,0445	99,0437	59,0123	57,0330	
21	Kaempferol-O-dirhamnosylhexoside	C33H40O19	24,25		739,20856	739.20910	0.73	285,0409	284,0331	255,0295	227,0343	151,0034	
22	Quercetin-O-rhamnosyldihexoside	C33H40O21	24,73		771,19839	771.19893	0.70	301,0358	300,0275	271,0245	255,0292		
23	Naringenin-O-glucuronide	C21H20O11	24,98		447,09274	447.09329	1.22	271,0615	175,0239	151,0025	113,0230	85,0280	
24[1]	Hyperoside (Quercetin-3-O-galactoside)	C21H20O12	25,19		463,08765	463.08820	1.19	301,0355	300,0277	271,0250	255,0297	151,0025	
25[1]	Rutin (Quercetin-3-O-rutinoside)	C27H30O16	25,24		609,14557	609.14611	0.88	301,0355	300,0277	271,0250	255,0297	151,0024	
26[1]	Isoquercitrin (Quercetin-3-O-glucoside)	C21H20O12	25,37		463,08765	463.08820	1.19	301,0356	300,0277	271,0250	255,0293	151,0026	
27	Dimethoxycinnamic acid	C11H12O4	25,54	209,08139		209.08084	2.65	191,0704	176,0472	163,0753	148,0520	105,0702	
28	Quercetin-O-pentoside isomer 1	C20H18O11	25,64		433,07709	433.07763	1.26	301,0352	300,0270	271,0250	255,0292	151,0023	
29[1]	Cosmosiin (Apigenin-7-O-glucoside)	C21H20O10	26,21		431,09782	431.09837	1.28	269,0459	268,0379	151,0023	149,0228		
30	Azelaic acid (Nonanedioic acid)	C9H16O4	26,31		187,09704	187.09758	2.90	169,0858	143,1067	125,0958	123,0800	97,0644	
31	Chrysoeriol-7-O-glucoside	C22H22O11	26,47		461,10839	461.10894	1.18	446,0858	299,0570	298,0485	283,0251	255,0297	
32	Quercetin-O-pentoside isomer 2	C20H18O11	26,57		433,07709	433.07763	1.26	301,0369	300,0272	271,0240	255,0296	151,0022	
33	O-p-Coumaroyl-O-hydroxybenzoyl-rhamnosylhexoside	C28H32O14	26,73		591,17138	591.17193	0.93	445,1140	307,0826	163,0389	145,0283	137,0232	
34[1]	Eriodictyol (3′,4′,5,7-Tetrahydroxyflavanone)	C15H12O6	26,90		287,05556	287.05611	1.92	151,0025	135,0438	125,0230	107,0123		
35[1]	Astragalin (Kaempferol-3-O-glucoside)	C21H20O11	26,98		447,09274	447.09329	1.22	285,0407	284,0327	255,0299	227,0347	151,0022	
36[1]	Apigenin-7-O-glucuronide	C21H18O11	27,06		445,07709	445.07763	1.22	269,0458	225,0552	175,0236	161,0235	113,0230	
37	Kaempferol-3-O-rutinoside (Nicotiflorin)	C27H30O15	27,11		593,15065	593.15119	0.92	327,0520	285,0408	284,0330	255,0298	227,0347	
38	Isorhamnetin-O-hexoside	C22H22O12	27,17		477,10330	477.10385	1.15	314,0440	299,0197	285,0418	271,0251	243,0291	
39	O-Feruloyl-O-hydroxybenzoyl-hexose	C23H24O11	27,21		475,12404	475.12459	1.15	337,0934	193,0494	175,0390	160,0154	137,0232	

40	Isorhamnetin-3-O-rutinoside (Narcissin)	$C_{28}H_{32}O_{16}$	27,45		623,16122	623.16176	0.86	315,0515	314,0436	300,0278	299,0201	271,0252
41[1]	Naringenin (4',5,7-Trihydroxyflavanone)	$C_{15}H_{12}O_5$	29,19		271,06065	271.06120	2.02	177,0185	165,0178	151,0025	119,0488	107,0125
42[1]	Quercetin (3,3',4',5,7-Pentahydroxyflavone)	$C_{15}H_{10}O_7$	29,29		301,03483	301.03538	1.81	273,0411	245,0451	178,9978	151,0025	121,0281
43[1]	Luteolin (3',4',5,7-Tetrahydroxyflavone)	$C_{15}H_{10}O_6$	30,26		285,03991	285.04046	1.94	217,0503	199,0393	175,0389	151,0025	133,0282
44[1]	Kaempferol (3,4',5,7-Tetrahydroxyflavone)	$C_{15}H_{10}O_6$	31,50		285,03991	285.04046	1.94	257,0468	229,0502	213,0555	151,0022	
45[1]	Isorhamnetin (3'-Methoxy-3,4',5,7-tetrahydroxyflavone)	$C_{16}H_{12}O_7$	31,99		315,05048	315.05103	1.73	300,0278	283,0252	271,0243	164,0104	151,0026
46[1]	Apigenin (4',5,7-Trihydroxyflavone)	$C_{15}H_{10}O_5$	32,01		269,04500	269.04555	2.03	227,0344	225,0549	151,0025	149,0233	117,0331
47	Methyl-trihydroxyxanthone	$C_{14}H_{10}O_5$	32,15		257,04500	257.04555	2.13	215,0344	213,0559			
48[1]	Chrysoeriol (3'-Methoxy-4',5,7-trihydroxyflavone)	$C_{16}H_{12}O_6$	32,26		299,05556	299.05611	1.84	284,0330	256,0377	227,0344	151,0026	107,0122
49	Malyngic acid (9,12,13-Trihydroxy-10E,15Z-octadecadienoic acid)	$C_{18}H_{32}O_5$	33,63		327,21715	327.21770	1.67	309,2079	291,1975	229,1442	211,1334	171,1016
50	Pinellic acid (9,12,13-Trihydroxy-10E-octadecenoic acid)	$C_{18}H_{34}O_5$	34,84		329,23280	329.23335	1.66	311,2231	293,2143	229,1441	211,1334	99,0802
51	Dihydroxy-methoxy(iso)flavone	$C_{16}H_{12}O_5$	35,59		283,06065	283.06120	1.93	268,0379	240,0417	151,0024		
52	Artemetin (5-Hydroxy-3,3',4',6,7-pentamethoxyflavone)	$C_{20}H_{20}O_8$	35,73	389,12365		389.12309	1.43	373,0916	359,0757	356,0891	331,0809	313,0704
53	Kumatakenin (4',5-Dihydroxy-3,7-dimethoxyflavone)	$C_{17}H_{14}O_6$	35,97		313,07122	313.07176	1.73	298,0486	297,0403	283,0249	270,0539	255,0299
54	Hydroxy-trimethoxy(iso)flavone	$C_{18}H_{16}O_6$	37,37	329,10251		329.10196	1.66	314,0781	313,0704	300,0632	299,0544	285,0755
55	Apigenin-4',7-dimethyl ether (4',7-Dimethoxy-5-hydroxyflavone)	$C_{17}H_{14}O_5$	39,05	299,09195		299.09140	1.84	284,0678	256,0727	167,0341		

[1] Confirmed by standard

Table S6. Chemical composition of the water extract of *Saponaria pumila*

No.	Name	Formula	Rt	$[M + H]^+$	$[M - H]^-$	Theoretical m/z	|Δppm|	Fragment 1	Fragment 2	Fragment 3	Fragment 4	Fragment 5
1	Quinic acid	$C_7H_{12}O_6$	2,15		191,05557	191.05611	2.84	127,0388	111,0439	93,0331	85,0280	
2	Citric acid	$C_6H_8O_7$	2,86		191,01918	191.01973	2.86	173,0080	129,0181	111,0073	87,0072	85,0280
3[1]	Tryptamine	$C_{10}H_{12}N_2$	8,18	161,10788		161.10732	3.45	144,0808	143,0732	117,0701	115,0545	103,0548
4[1]	Catechin	$C_{15}H_{14}O_6$	15,88		289,07121	289.07176	1.91	245,0813	203,0699	151,0385	123,0437	109,0278
5[1]	Caffeic acid	$C_9H_8O_4$	17,49		179,03444	179.03498	3.03	135,0440	107,0493			
6	Licoagroside B or isomer	$C_{18}H_{24}O_{12}$	18,39		431,11896	431.11950	1.25	203,0557	161,0442	125,0230	99,0437	57,0330
7	Kynurenic acid	$C_{10}H_7NO_3$	18,64	190,05042		190.04987	2.90	162,0549	144,0445	116,0499	89,0391	
8[1]	Epicatechin	$C_{15}H_{14}O_6$	19,09		289,07121	289.07176	1.91	245,0814	203,0710	151,0393	123,0433	109,0278
9	Quercetin-di-O-rhamnosylhexoside isomer 1	$C_{39}H_{50}O_{25}$	20,00		917,25630	917.25684	0.59	609,1467	608,1395	301,0356	299,0201	271,0253

10	Dihydroferulic acid (3-(4-Hydroxy-3-methoxyphenyl)propionic acid)	$C_{10}H_{12}O_4$	20,44		195,06568	195.06628	3.09	151,0388	136,0517	135,0440	121,0281	119,0488
11[1]	Riboflavin	$C_{17}H_{20}N_4O_6$	20,47	377,14611		377.14556	1.46	359,1361	243,0877	216,0769	200,0822	172,0869
12[1]	p-Coumaric acid	$C_9H_8O_3$	20,67		163,03952	163.04007	3.36	119,0488				
13	Phaselic acid (2-O-Caffeoylmalic acid)	$C_{13}H_{12}O_8$	20,70		295,04540	295.04594	1.83	179,0345	135,0440	133,0131	115,0025	71,0124
14[1]	Ferulic acid	$C_{10}H_{10}O_4$	21,81		193,05009	193.05063	2.81	178,0262	149,0595	137,0235	134,0361	121,0281
15[1]	Sinapic acid	$C_{11}H_{12}O_5$	22,29		223,06065	223.06120	2.45	208,0371	193,0134	179,0708	164,0468	149,0232
16	Quercetin-di-O-rhamnosylhexoside isomer 2	$C_{39}H_{50}O_{25}$	22,74		917,25630	917.25684	0.59	609,1470	301,0355	300,0278	271,0253	151,0021
17	Quercetin-di-O-rhamnosylhexoside isomer 3	$C_{39}H_{50}O_{25}$	23,34		917,25630	917.25684	0.59	609,1470	301,0358	300,0278	271,0236	151,0026
18	O-Benzyl-O-(3-hydroxy-3-methylglutaroyl)-hexose	$C_{19}H_{26}O_{10}$	23,72		413,14477	413.14532	1.33	269,1034	161,0444	99,0438	59,0123	57,0330
19	Naringenin-O-glucuronide	$C_{21}H_{20}O_{11}$	24,98		447,09274	447.09329	1.22	271,0615	175,0238	151,0025	113,0230	85,0280
20	Quercetin-O-dirhamnosyldihexoside	$C_{39}H_{50}O_{25}$	25,15		917,25630	917.25684	0.59	301,0356	300,0278	271,0250	255,0297	151,0024
21[1]	Hyperoside (Quercetin-3-O-galactoside)	$C_{21}H_{20}O_{12}$	25,24		463,08765	463.08820	1.19	301,0356	300,0277	271,0251	255,0296	151,0024
22[1]	Rutin (Quercetin-3-O-rutinoside)	$C_{27}H_{30}O_{16}$	25,29		609,14557	609.14611	0.88	301,0356	300,0277	271,0250	255,0297	151,0024
23[1]	Isoquercitrin (Quercetin-3-O-glucoside)	$C_{21}H_{20}O_{12}$	25,45		463,08765	463.08820	1.19	301,0355	300,0277	271,0251	255,0297	151,0024
24	Dimethoxycinnamic acid	$C_{11}H_{12}O_4$	25,52	209,08139		209.08084	2.65	191,0703	176,0469	163,0753	148,0519	105,0701
25	Azelaic acid (Nonanedioic acid)	$C_9H_{16}O_4$	26,30		187,09704	187.09758	2.90	169,0857	143,1065	125,0958	123,0801	97,0645
26[1]	Eriodictyol (3′,4′,5,7-Tetrahydroxyflavanone)	$C_{15}H_{12}O_6$	26,92		287,05556	287.05611	1.92	151,0024	135,0440	125,0229	107,0123	
27[1]	Astragalin (Kaempferol-3-O-glucoside)	$C_{21}H_{20}O_{11}$	26,97		447,09274	447.09329	1.22	285,0408	284,0330	255,0298	227,0344	151,0023
28[1]	Apigenin-7-O-glucuronide	$C_{21}H_{18}O_{11}$	27,07		445,07709	445.07763	1.22	269,0458	225,0552	175,0232	161,0237	113,0230
29	Kaempferol-3-O-rutinoside (Nicotiflorin)	$C_{27}H_{30}O_{15}$	27,11		593,15065	593.15119	0.92	327,0522	285,0409	284,0332	255,0300	227,0342
30	Isorhamnetin-O-hexoside	$C_{22}H_{22}O_{12}$	27,18		477,10330	477.10385	1.15	314,0437	299,0202	285,0408	271,0251	243,0298
31	Isorhamnetin-3-O-rutinoside (Narcissin)	$C_{28}H_{32}O_{16}$	27,46		623,16122	623.16176	0.86	315,0515	314,0437	300,0277	299,0199	271,0251
32[1]	Naringenin (4′,5,7-Trihydroxyflavanone)	$C_{15}H_{12}O_5$	29,18		271,06065	271.06120	2.02	177,0188	165,0181	151,0026	119,0489	107,0125
33[1]	Quercetin (3,3′,4′,5,7-Pentahydroxyflavone)	$C_{15}H_{10}O_7$	29,29		301,03483	301.03538	1.81	273,0412	245,0460	178,9979	151,0026	121,0281
34[1]	Luteolin (3′,4′,5,7-Tetrahydroxyflavone)	$C_{15}H_{10}O_6$	30,28		285,03991	285.04046	1.94	217,0501	199,0390	175,0388	151,0023	133,0279

35[1]	Kaempferol (3,4',5,7-Tetrahydroxyflavone)	C15H10O6	31,50		285,03991	285.04046	1.94	257,0449	229,0500	213,0552	151,0023	
36[1]	Isorhamnetin (3'-Methoxy-3,4',5,7-tetrahydroxyflavone)	C16H12O7	31,98		315,05048	315.05103	1.73	300,0277	283,0252	271,0252	164,0103	151,0025
37[1]	Apigenin (4',5,7-Trihydroxyflavone)	C15H10O5	32,00		269,04500	269.04555	2.03	227,0351	225,0551	151,0024	149,0232	117,0331
38[1]	Chrysoeriol (3'-Methoxy-4',5,7-trihydroxyflavone)	C16H12O6	32,25		299,05556	299.05611	1.84	284,0329	256,0369	227,0342	151,0017	107,0120
39	Malyngic acid (9,12,13-Trihydroxy-10E,15Z-octadecadienoic acid)	C18H32O5	33,63		327,21715	327.21770	1.67	309,2073	291,1981	229,1444	211,1335	171,1018
40	Pinellic acid (9,12,13-Trihydroxy-10E-octadecenoic acid)	C18H34O5	34,84		329,23280	329.23335	1.66	311,2232	293,2127	229,1444	211,1335	99,0802

[1] Confirmed by standard

Table S7. Docking scores of the molecular library against key enzymes from *A. fumigatus*, *A. niger*, *B. cereus*, *E. coli*, and *L. monocytogenes*.

Molecules/Enzymes	Thioredoxin reductase (*A. fumigatus*)	Endoglucanase A (*A. niger*)	Sphingomyelinase C (*B. cereus*)	DNA gyrase (*E. coli*)	Listeriolysin O (*L. monocytogenes*)
Apigenin	-79.51	-69.78	-64.54	-65.43	-61.70
Astragalin	-67.23	-81.60	-67.61	-66.99	-67.24
Azelaic acid	-73.65	-70.75	-76.56	-61.39	-55.81
Caffeic acid	-75.46	-61.99	-58.98	-54.88	-60.04
Catechin	-82.75	-77.28	-71.30	-61.90	-62.56
Chrysoeriol	-79.98	-68.40	-65.77	-64.66	-60.59
Dihydroferulic acid	-73.56	-60.38	-68.98	-57.33	-55.11
Dimethoxycinnamic acid	-75.79	-57.75	-55.50	-52.52	-52.47
Epicatechin	-78.64	-74.29	-65.91	-64.96	-63.76

Eriodictyol	-82.17	-72.01	-64.48	-62.24	-66.33
Homoeriodictyol	-79.44	-70.82	-64.05	-63.60	-62.56
Hyperoside	-73.41	-81.05	-69.99	-66.68	-66.91
Icariside B5	-90.51	-71.58	-72.98	-62.23	-64.11
Isoquercitrin	-71.68	-84.22	-66.30	-68.18	-70.65
Isorhamnetin	-85.26	-69.97	-63.42	-65.82	-62.93
Jaslanceoside B	-93.88	-90.34	-63.33	-80.52	-60.14
Kaempferol	-85.12	-68.10	-63.84	-61.69	-58.41
Luteolin	-85.92	-71.82	-64.48	-65.54	-66.97
Malyngic acid	-99.63	-92.81	-89.90	-90.06	-71.67
Pinellic acid	-105.43	-83.35	-91.99	-86.84	-74.61
Quercetin	-90.10	-72.59	-61.81	-64.18	-63.38
Rutin	-87.24	-99.52	-61.68	-73.40	-67.44
Tomentin	-74.32	-51.59	-54.28	-47.01	-48.05

Table S8. Docking scores of the molecular library against key enzymes from *P. aeruginosa*, *P. cyclopium*, *P. funiculosum*, *S. aureus*, and *T. viride*.

**Molecules/Enzymes**	**Arginine Rhamnosyltransferase (*P. aeruginosa*)**	**mono-/diacylglycerol lipase (*P. cyclopium*)**	**endo-1,4-β-xylanase C (*P. funiculosum*)**	**tyrosyl-tRNA synthetase (*S. aureus*)**	**L-lysine oxidase (*T. viride*)**
Apigenin	-58.71	-66.87	-82.25	-78.13	-86.76
Astragalin	-66.67	-61.57	-79.34	-79.18	-66.55
Azelaic acid	-67.67	-65.24	-78.45	-73.64	-75.94
Caffeic acid	-63.04	-66.01	-77.57	-69.78	-75.87

Catechin	-62.91	-65.07	-88.07	-79.39	-99.10
Chrysoeriol	-60.05	-63.13	-83.90	-80.72	-89.81
Dihydroferulic acid	-68.28	-63.04	-74.56	-72.34	-76.49
Dimethoxycinnamic acid	-62.99	-56.43	-73.24	-65.79	-71.91
Epicatechin	-65.13	-63.35	-90.16	-82.80	-88.13
Eriodictyol	-63.40	-68.90	-89.87	-81.77	-90.39
Homoeriodictyol	-62.09	-66.02	-86.84	-80.64	-93.71
Hyperoside	-60.71	-62.95	-78.67	-84.65	-75.77
Icariside B5	-73.76	-71.76	-79.55	-86.56	-88.73
Isoquercitrin	-64.45	-57.71	-80.86	-83.47	-73.74
Isorhamnetin	-62.00	-63.24	-82.76	-78.01	-97.93
Jaslanceoside B	-62.29	-67.08	-62.17	-60.87	4.19
Kaempferol	-59.57	-63.36	-80.23	-75.96	-95.85
Luteolin	-64.66	-67.08	-85.75	-82.83	-87.98
Malyngic acid	-80.99	-77.60	-105.54	-100.64	-107.79
Pinellic acid	-85.06	-81.80	-97.88	-101.27	-112.88
Quercetin	-61.48	-66.30	-85.03	-82.22	-96.23
Rutin	-66.97	-57.63	-57.45	-85.24	-47.19
Tomentin	-54.29	-60.63	-74.28	-63.92	-69.21

Table S9. Free energy components of five selected molecules in the complex with Thioredoxin reductase protein (*A. fumigatus*).

Parameters/Enzymes	Icariside B5	Jaslanceoside B	Malyngic acid	Pinellic acid	Quercetin
ΔVDWAALS	-39.88	-27.24	-43.73	-43.75	-30.67
ΔEEL	-4.06	-1.76	-4.62	-5.53	-3.13
ΔEGB	4.95	3.04	5.42	6.12	4.10

ΔESURF	-6.33	-4.23	-7.08	-7.44	-4.38
ΔGGAS	-43.94	-29.00	-48.35	-49.28	-33.80
ΔGSOLV	-1.37	-1.19	-1.66	-1.32	-0.27
ΔTOTAL	-45.32	-30.19	-50.02	-50.61	-34.07

Table S10. Free energy components of five selected molecules in the complex with Endoglucanase A protein (*A. niger*).

Parameters/Enzymes	Isoquercitrin	Jaslanceoside B	Malyngic acid	Pinellic acid	Rutin
ΔVDWAALS	-37.38	-24.38	-32.04	-35.37	-44.79
ΔEEL	-7.31	-3.00	-2.58	-3.57	-4.72
ΔEGB	7.45	3.81	3.59	3.86	6.25
ΔESURF	-6.17	-4.17	-5.02	-5.35	-7.35
ΔGGAS	-44.69	-27.38	-34.62	-38.94	-49.50
ΔGSOLV	1.27	-0.36	-1.43	-1.49	-1.10
ΔTOTAL	-43.42	-27.74	-30.06	-40.43	-50.61

Table S11. Free energy components of five selected molecules in the complex with Sphingomyelinase C protein (*B. cereus*).

Parameters/Enzymes	Azelaic acid	Catechin	Icariside B5	Malyngic acid	Pinellic acid
ΔVDWAALS	-2.19	-22.15	-27.30	-23.72	-11.97
ΔEEL	-0.56	-3.02	-1.70	-1.76	-1.08
ΔEGB	0.56	3.41	2.49	2.55	1.34
ΔESURF	-0.46	-3.63	-3.88	-4.04	-1.97
ΔGGAS	-2.75	-3.63	-29.62	-25.49	-13.04
ΔGSOLV	0.10	-0.22	-1.39	-1.49	-0.63
ΔTOTAL	-2.65	-25.39	-30.39	-26.98	-13.67

Table S12. Free energy components of five selected molecules in the complex with DNA gyrase protein (*E. coli*).

Parameters/Enzymes	Isoquercitrin	Jaslanceoside B	Malyngic acid	Pinellic acid	Rutin
ΔVDWAALS	-26.52	-28.25	-34.57	-36.72	-29.29
ΔEEL	-2.76	-4.55	-3.06	-2.97	-6.67
ΔEGB	3.92	4.80	3.27	3.05	6.18
ΔESURF	-4.14	-5.21	-5.78	-5.93	-4.97
ΔGGAS	-29.27	-32.79	-37.63	-39.70	-35.96
ΔGSOLV	0.22	-0.41	-2.51	-2.88	1.21
ΔTOTAL	-29.49	-33.21	-40.15	-42.58	-34.74

Table S13. Free energy components of five selected molecules in the complex with Listeriolysin O protein (*L. monocytogenes*).

Parameters/Enzymes	Astragalin	Isoquercitrin	Malyngic acid	Pinellic acid	Rutin
ΔVDWAALS	-15.01	-18.04	-6.78	-22.94	-18.93
ΔEEL	-0.68	-2.95	-0.61	-1.84	-3.55
ΔEGB	1.26	3.11	0.79	2.17	3.55
ΔESURF	-1.95	-2.73	-1.12	-3.79	-2.87
ΔGGAS	-15.69	-20.99	-7.39	-24.79	-22.48
ΔGSOLV	-0.68	0.38	-0.33	-1.61	0.68
ΔTOTAL	-16.37	-20.61	-7.72	-26.40	-21.80

Table S14. Free energy components of five selected molecules in the complex with Arginine Rhamnosyltransferase protein (*P. aeruginosa*).

Parameters/Enzymes	Azelaic acid	Dihydroferulic acid	Icariside B5	Malyngic acid	Pinellic acid
ΔVDWAALS	-16.88	-10.01	-23.90	-21.22	-24.71
ΔEEL	-1.08	-1.73	-2.09	-3.29	-2.02
ΔEGB	1.67	1.85	2.97	3.62	2.77
ΔESURF	-2.83	-1.80	-3.76	-3.99	-4.07
ΔGGAS	-17.97	-11.74	-25.99	-24.51	-26.72
ΔGSOLV	-1.16	0.04	-0.79	-0.37	-1.30
ΔTOTAL	-19.12	-11.70	-26.78	-24.88	-28.02

Table S15. Free energy components of five selected molecules in the complex with mono-/diacylglycerol lipase protein (*P. cyclopium*).

Parameters/Enzymes	Eriodictyol	Icariside B5	Luteolin	Malyngic acid	Pinellic acid
ΔVDWAALS	-14.59	-14.75	-21.35	-29.86	-26.59
ΔEEL	-1.25	-1.16	-3.66	-3.42	-2.25
ΔEGB	1.62	1.58	3.40	4.11	2.62
ΔESURF	-2.16	-2.33	-3.77	-4.88	-4.34
ΔGGAS	-15.85	-15.92	-25.01	-33.28	-28.84
ΔGSOLV	-0.54	-0.75	-0.37	-0.77	-1.72
ΔTOTAL	-16.39	-16.66	-25.38	-34.05	-30.56

Table S16. Free energy components of five selected molecules in the complex with endo-1,4-β-xylanase C protein (*P. funiculosum*).

Parameters/Enzymes	Catechin	Epicatechin	Eriodictyol	Malyngic acid	Pinellic acid
ΔVDWAALS	-15.34	-22.75	-19.35	-12.90	-30.57
ΔEEL	-1.47	-0.73	-1.11	-1.02	-1.94
ΔEGB	1.96	1.85	1.91	1.38	2.72
ΔESURF	-2.26	-2.82	-2.61	-2.11	-4.66
ΔGGAS	-16.81	-23.48	-20.46	-13.92	-32.51
ΔGSOLV	-0.30	-0.97	-0.70	-0.73	-1.94
ΔTOTAL	-17.11	-24.45	-21.16	-14.64	-34.45

Table S17. Free energy components of five selected molecules in the complex with tyrosyl-tRNA synthetase protein (*S. aureus*).

Parameters/Enzymes	Hyperoside	Icariside B5	Malyngic acid	Pinellic acid	Rutin
ΔVDWAALS	-41.54	-43.59	-45.21	-40.81	-56.51
ΔEEL	-6.97	-4.83	-3.42	-3.88	-6.36
ΔEGB	7.41	5.91	4.31	4.78	8.34

ΔESURF	-6.95	-6.72	-7.14	-6.52	-8.43
ΔGGAS	-48.51	-48.41	-48.62	-44.69	-62.88
ΔGSOLV	0.47	-0.80	-2.82	-1.74	-0.09
ΔTOTAL	-48.05	-49.22	-51.45	-46.43	-62.97

Table S18. Free energy components of five selected molecules in the complex with L-lysine oxidase protein (*T. viride*).

Parameters/Enzymes	Catechin	Isoquercitrin	Malyngic acid	Pinellic acid	Quercetin
ΔVDWAALS	-38.50	-40.85	-51.33	-45.65	-37.00
ΔEEL	-4.28	-2.76	-3.72	-3.63	-4.38
ΔEGB	5.24	4.38	5.16	5.11	5.51
ΔESURF	-5.24	-5.43	-7.74	-6.99	-5.22
ΔGGAS	-42.78	-43.61	-55.06	-49.28	-41.38
ΔGSOLV	0.00	-1.05	-2.58	-1.88	0.29
ΔTOTAL	-42.79	-44.66	-57.64	-51.16	-41.09

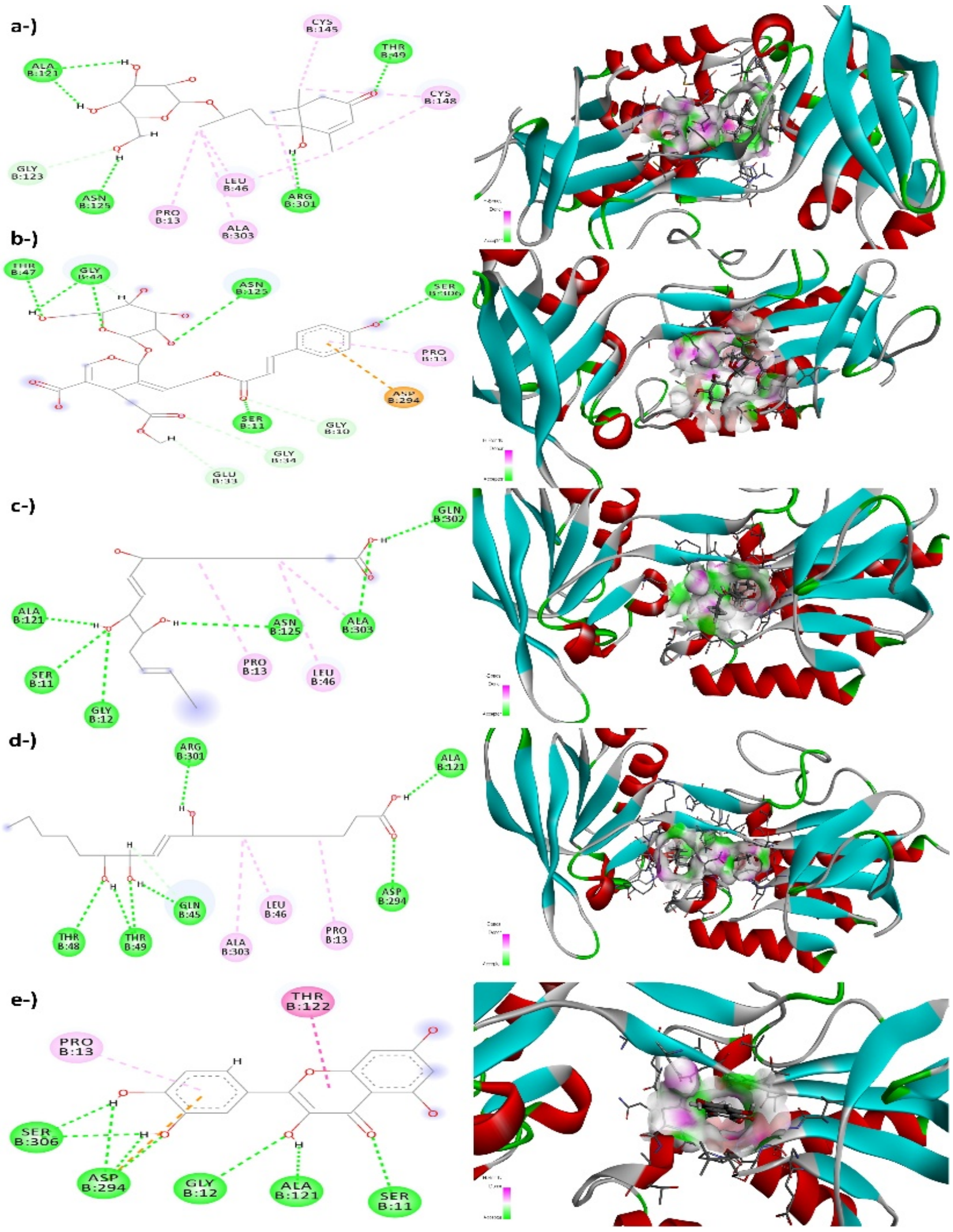

Figure S1. Interaction graphs of Thioredoxin reductase protein (*A. fumigatus*) with the five molecules with the lowest docking scores. a-) Icariside B5 b-) Jaslanceoside B c-) Malyngic acid d-) Pinellic acid e-) Quercetin.

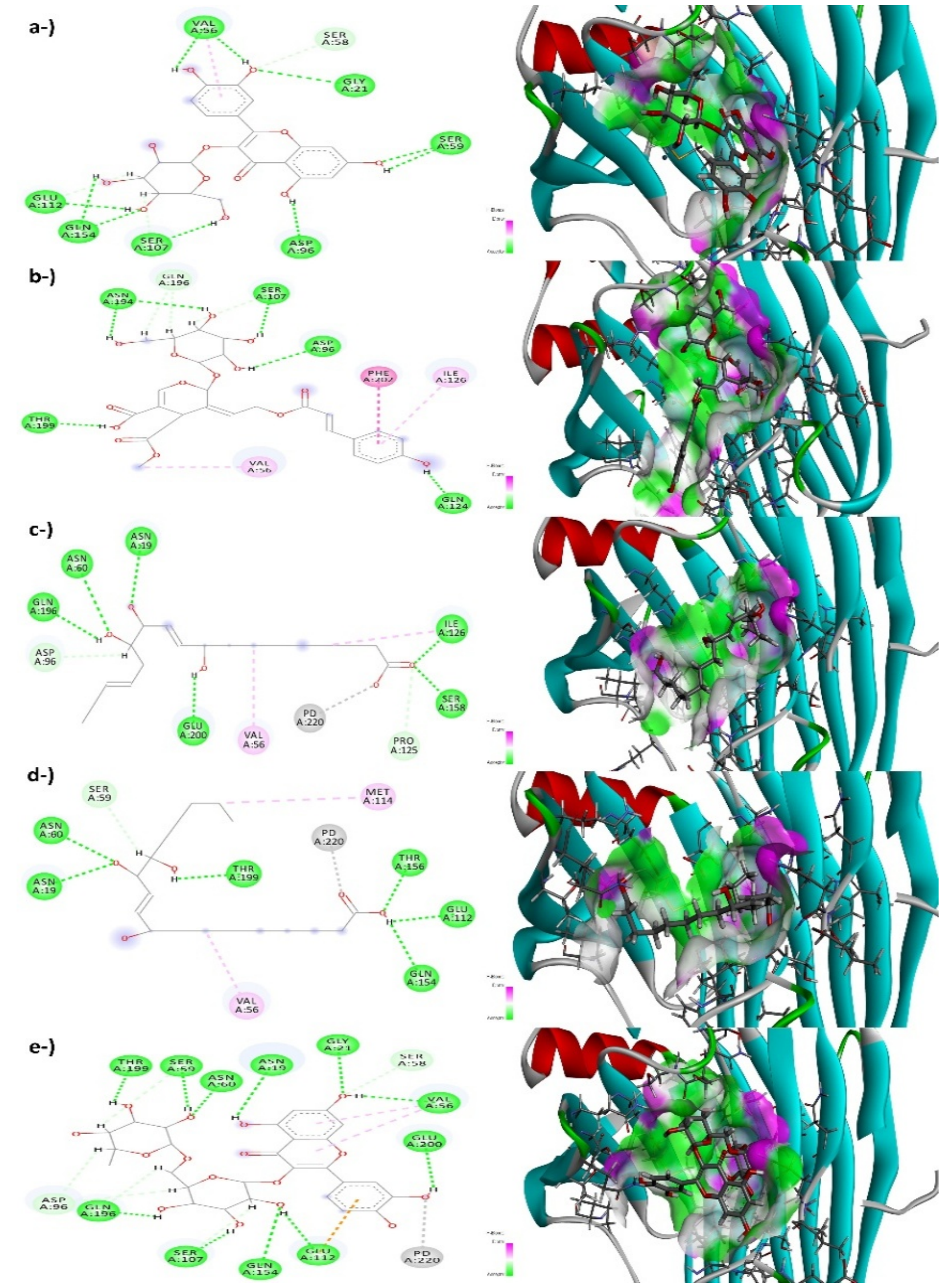

Figure S2. Interaction graphs of Endoglucanase A protein (*A. niger*) with the five molecules with the lowest docking scores. a-) Isoquercitrin b-) Jaslanceoside B c-) Malyngic acid d-) Pinellic acid e-) Rutin.

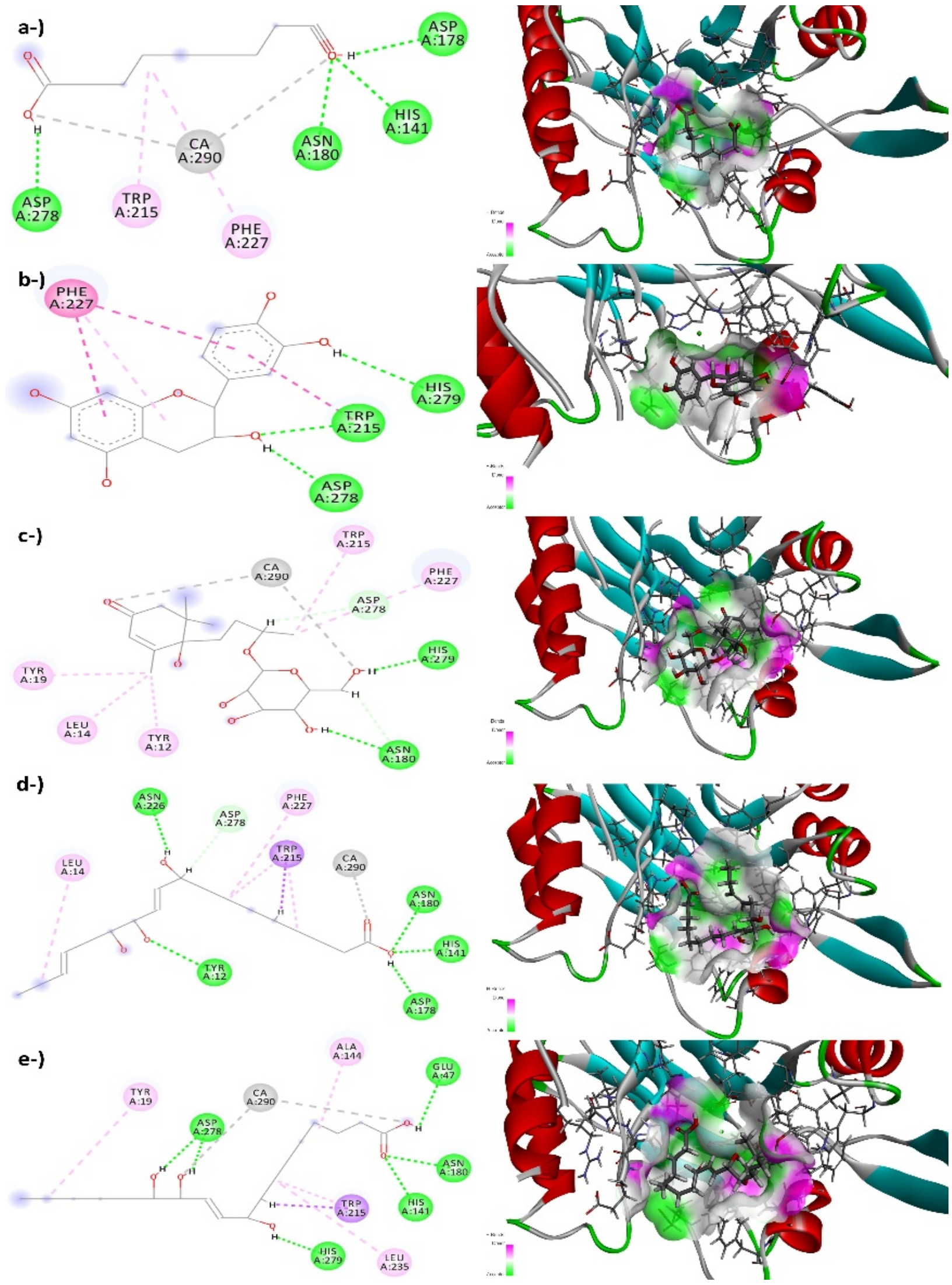

Figure S3. Interaction graphs of Sphingomyelinase C protein (*B. cereus*) with the five molecules with the lowest docking scores. a-) Azelaic acid b-) Catechin B c-) Icariside B5 d-) Malyngic acid e-) Pinellic acid.

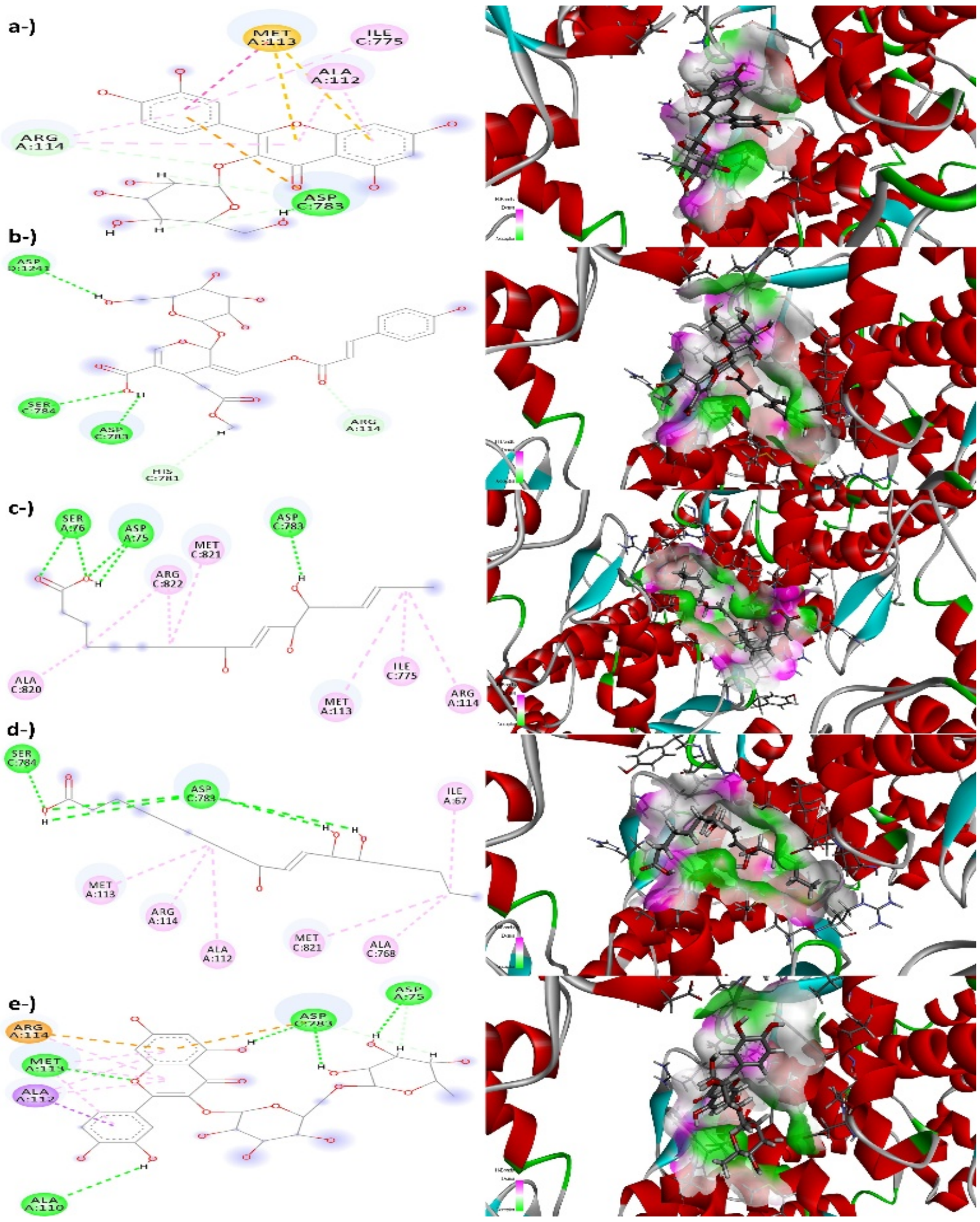

Figure S4. Interaction graphs of DNA gyrase protein (*E. coli*) with the five molecules with the lowest docking scores. a-) Isoquercitrin b-) Jaslanceoside B c-) Malyngic acid d-) Pinellic acid e-) Rutin.

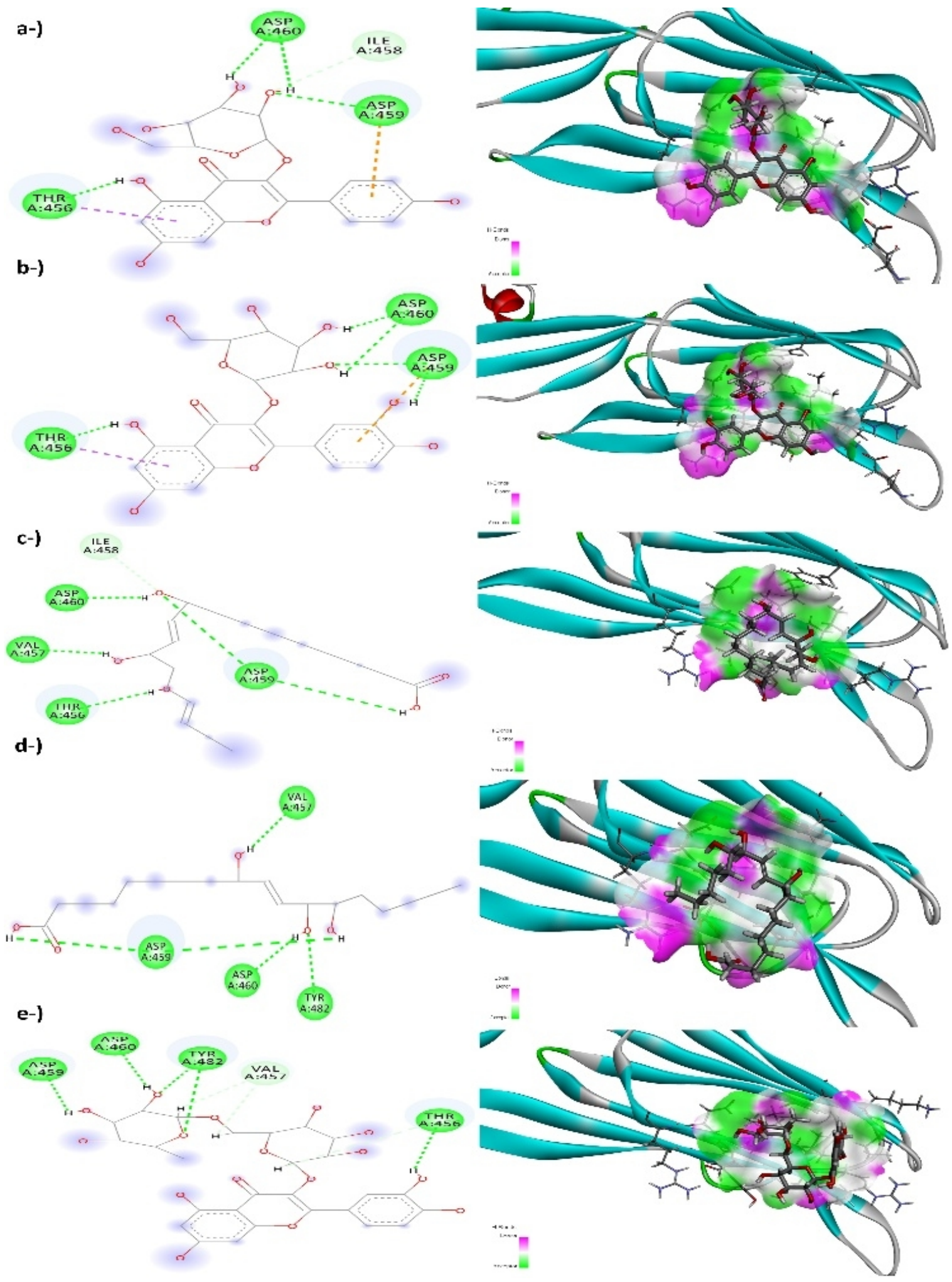

Figure S5. Interaction graphs of Listeriolysin O protein (*L. monocytogenes*) with the five molecules with the lowest docking scores. a-) Astragalin b-) Isoquercitrin c-) Malyngic acid d-) Pinellic acid e-) Rutin.

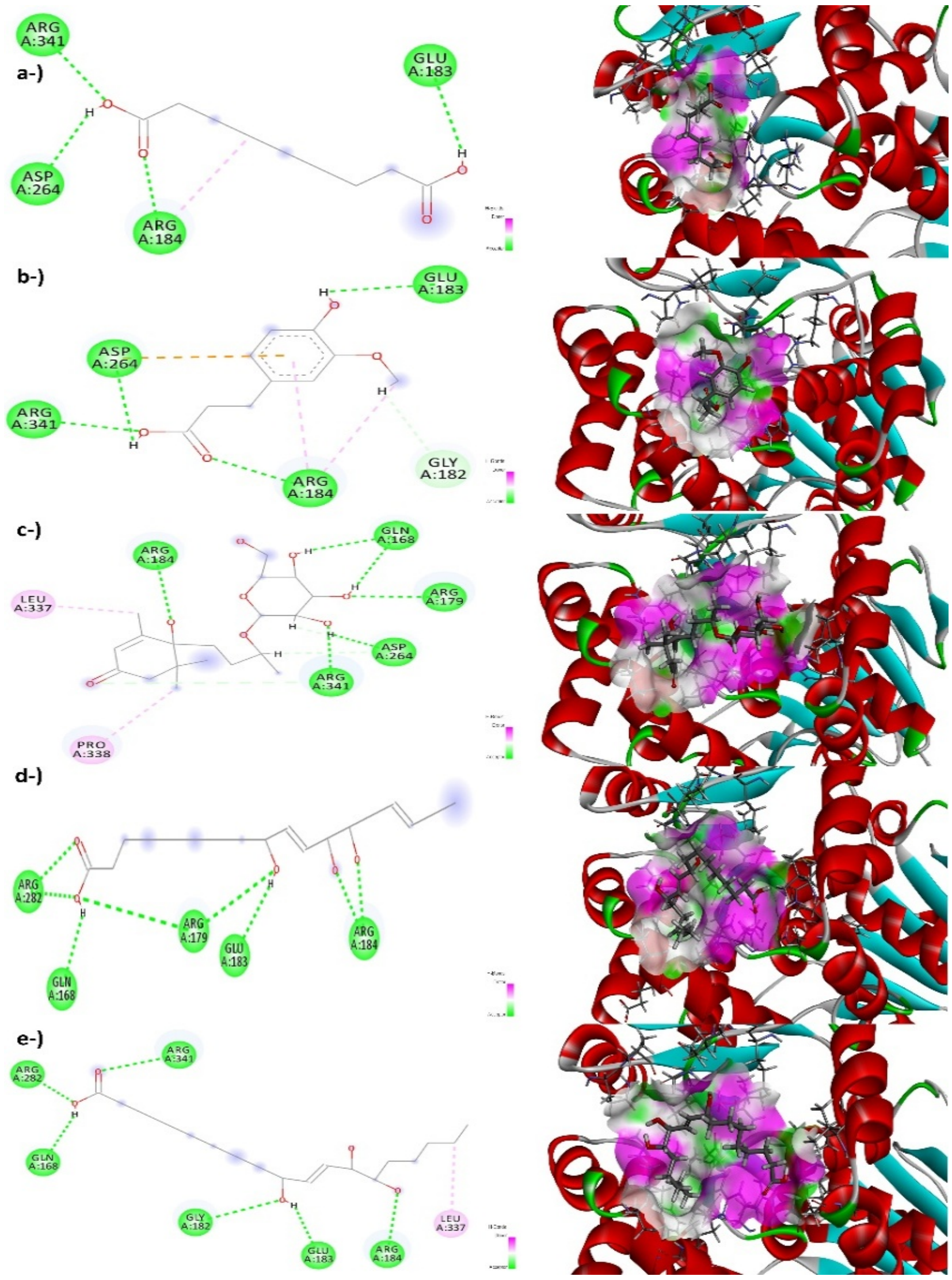

Figure S6. Interaction graphs of arginine rhamnosyltransferase protein (*P. aeruginosa*) with the five molecules with the lowest docking scores. a-) Azelaic acid b-) Dihydroferulic acid c-) Icariside B5 d-) Malyngic acid e-) Pinellic acid.

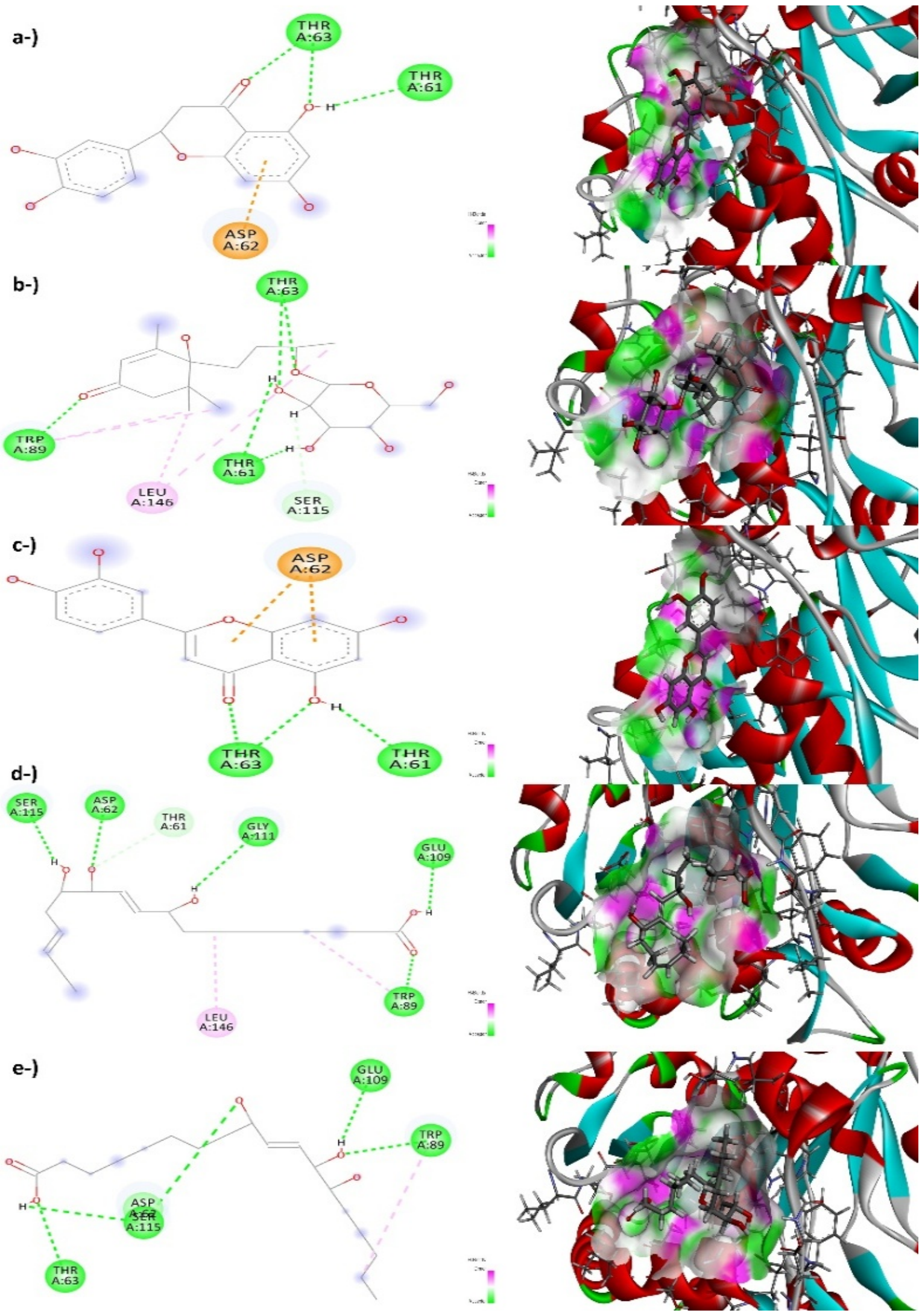

Figure S7. Interaction graphs of mono-/diacylglycerol lipase protein (*P. cyclopium*) with the five molecules with the lowest docking scores. a-) Eriodictyol b-) Icariside B5 c-) Luteolin d-) Malyngic acid e-) Pinellic acid.

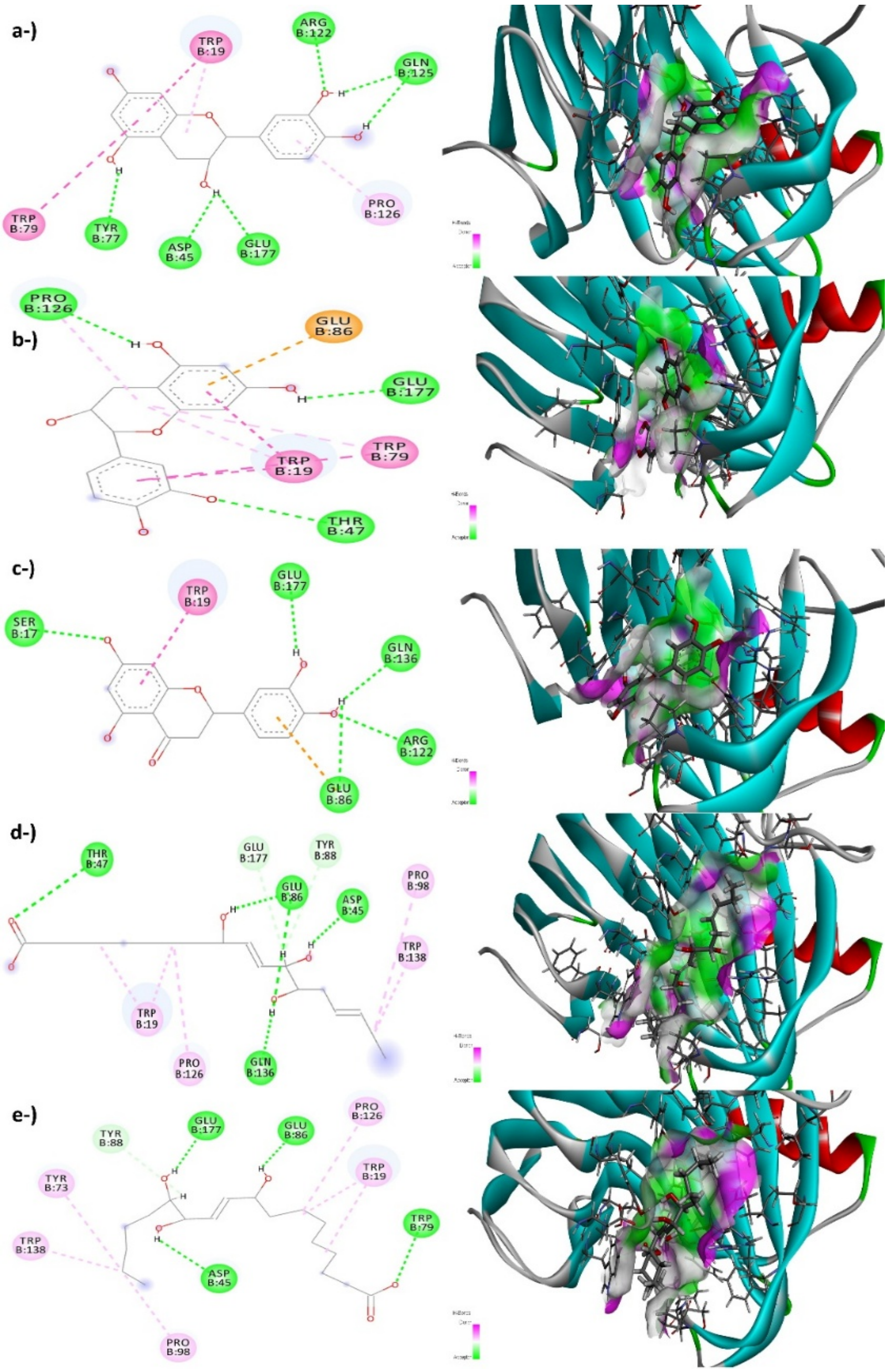

Figure S8. Interaction graphs of endo-1,4-β-xylanase C protein (*P. funiculosum*) with the five molecules with the lowest docking scores. a-) Catechin b-) Epicatechin c-) Eriodictyol d-) Malyngic acid e-) Pinellic acid.

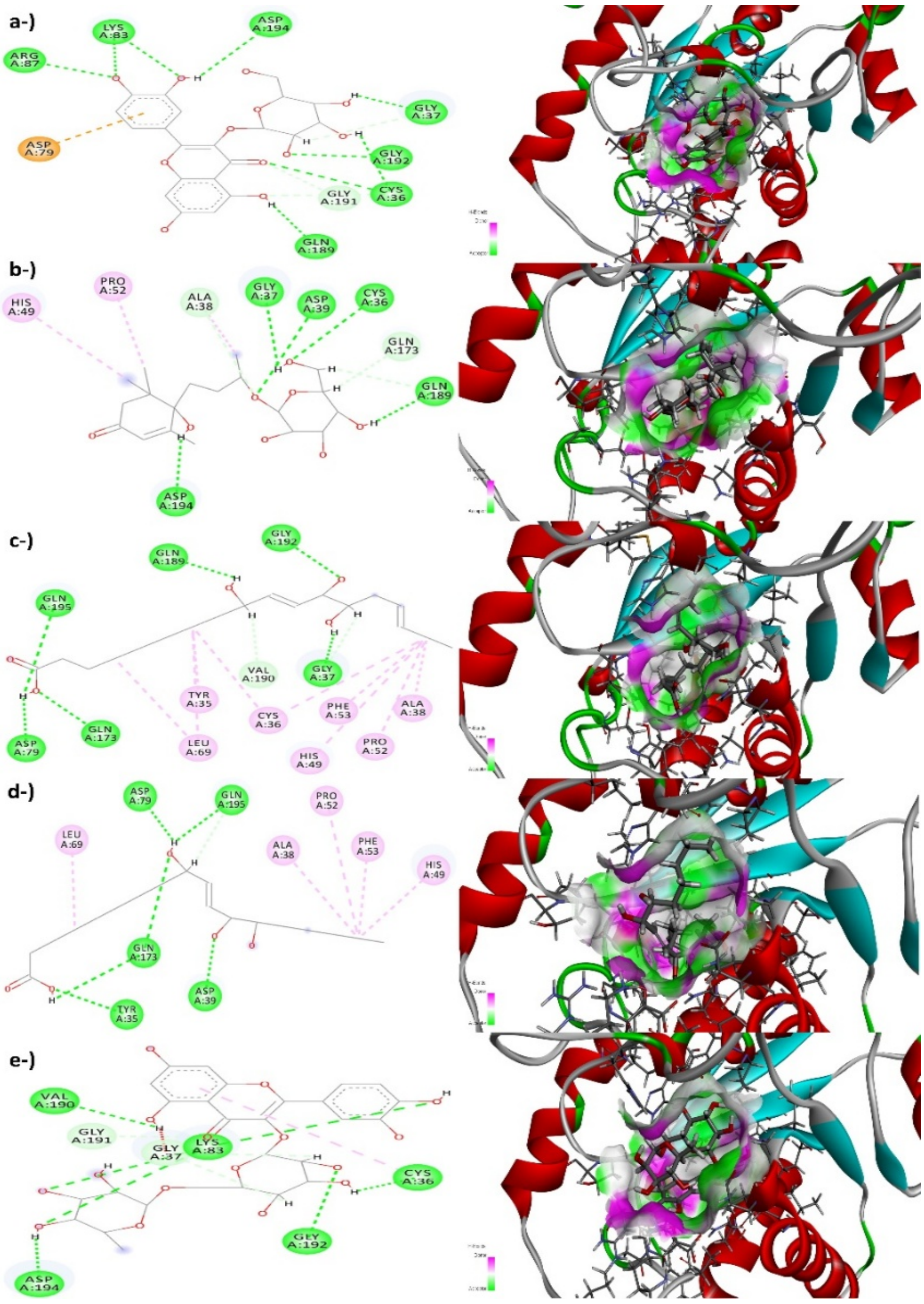

Figure S9. Interaction graphs of tyrosyl-tRNA synthetase protein (*S. aureus*) with the five molecules with the lowest docking scores. a-) Hyperoside b-) Icariside B5 B c-) Malyngic acid d-) Pinellic acid e-) Rutin.

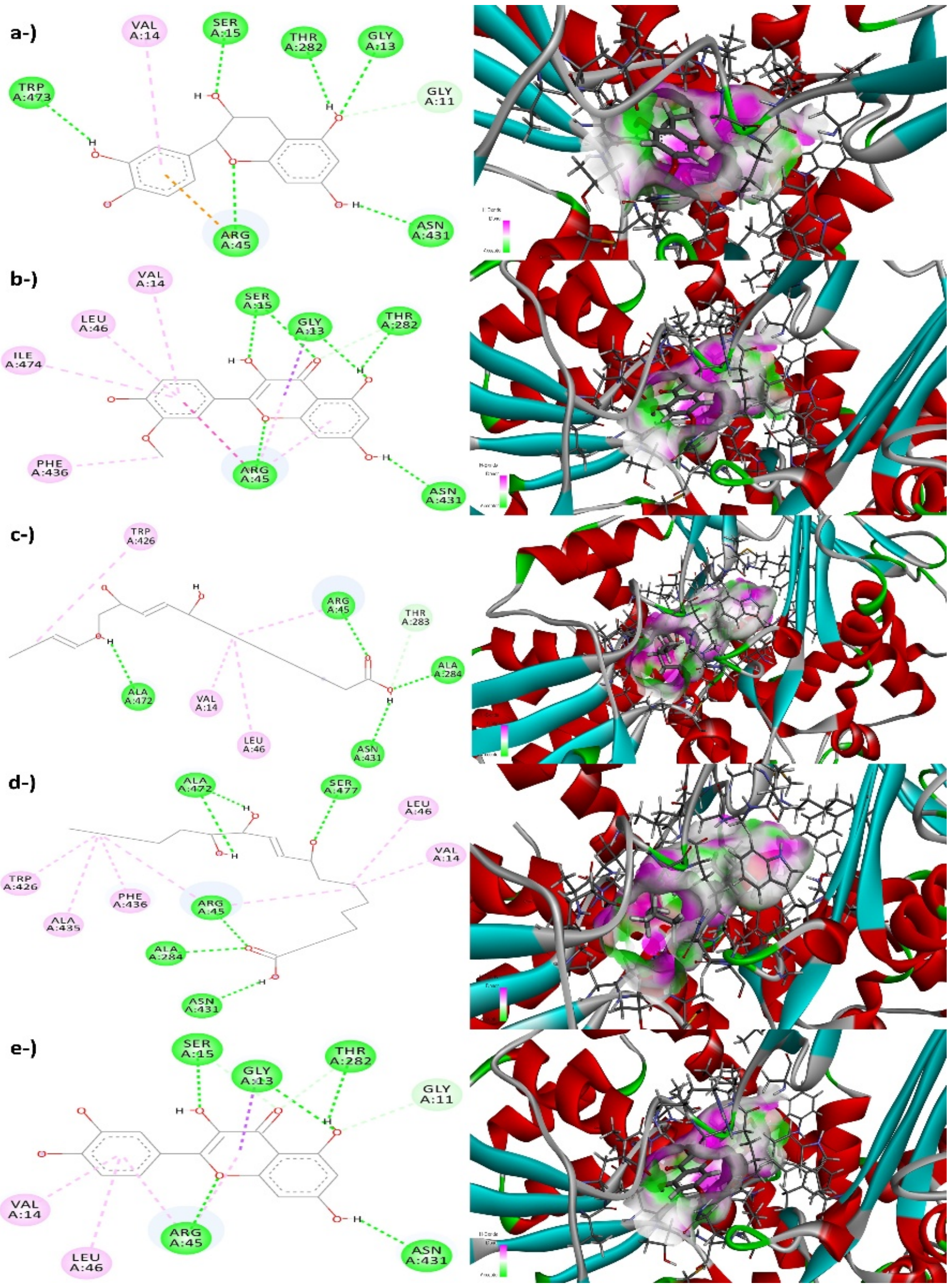

Figure S10. Interaction graphs of L-lysine oxidase protein (*T. viride*) with the five molecules with the lowest docking scores. a-) Catechin b-) Isoquercitrin B c-) Malyngic acid d-) Pinellic acid e-) Quercetin.

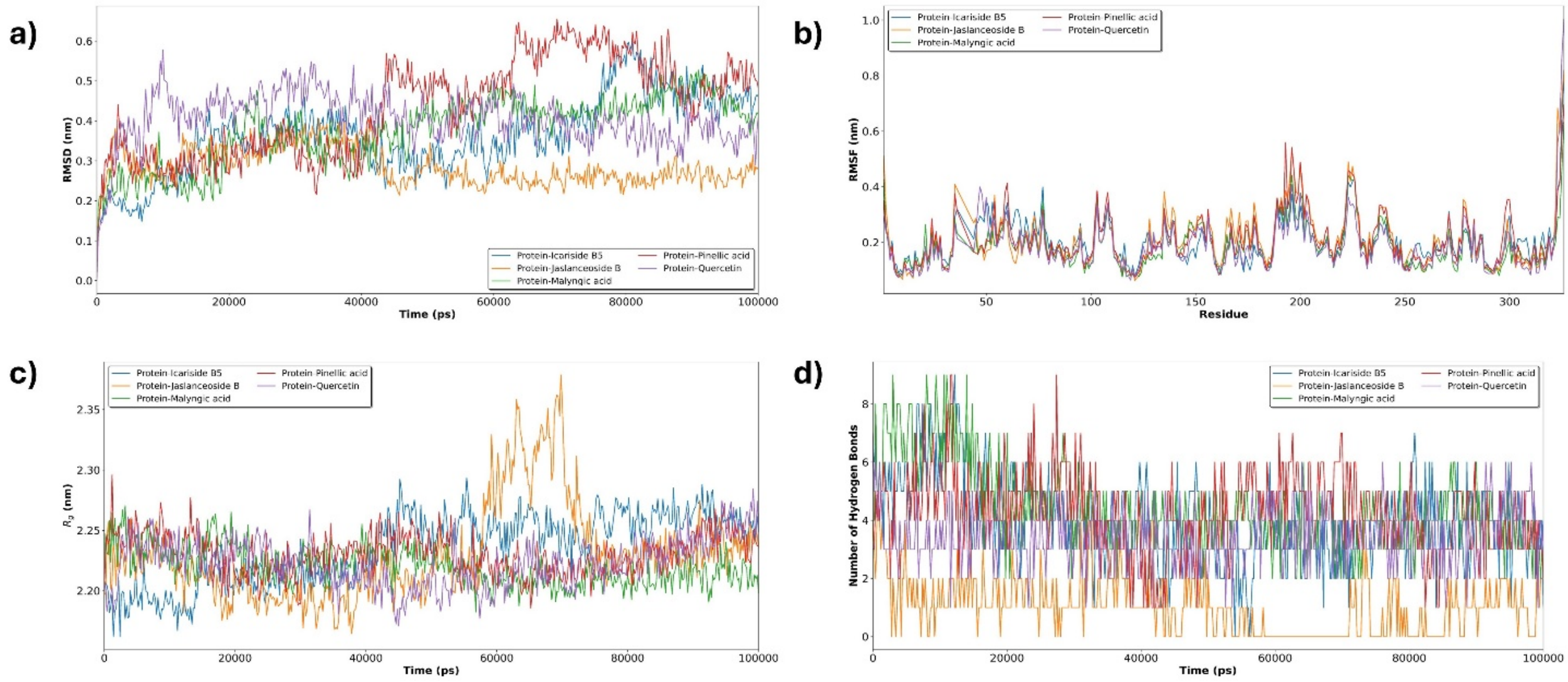

Figure S11. Plots of data obtained from the Molecular Dynamics (MD) simulation of the Thioredoxin reductase protein from *A. fumigatus* with the five best docking scores. a-) RMSD b-) RMSF c-) Radius of gyration d-) H-bond.

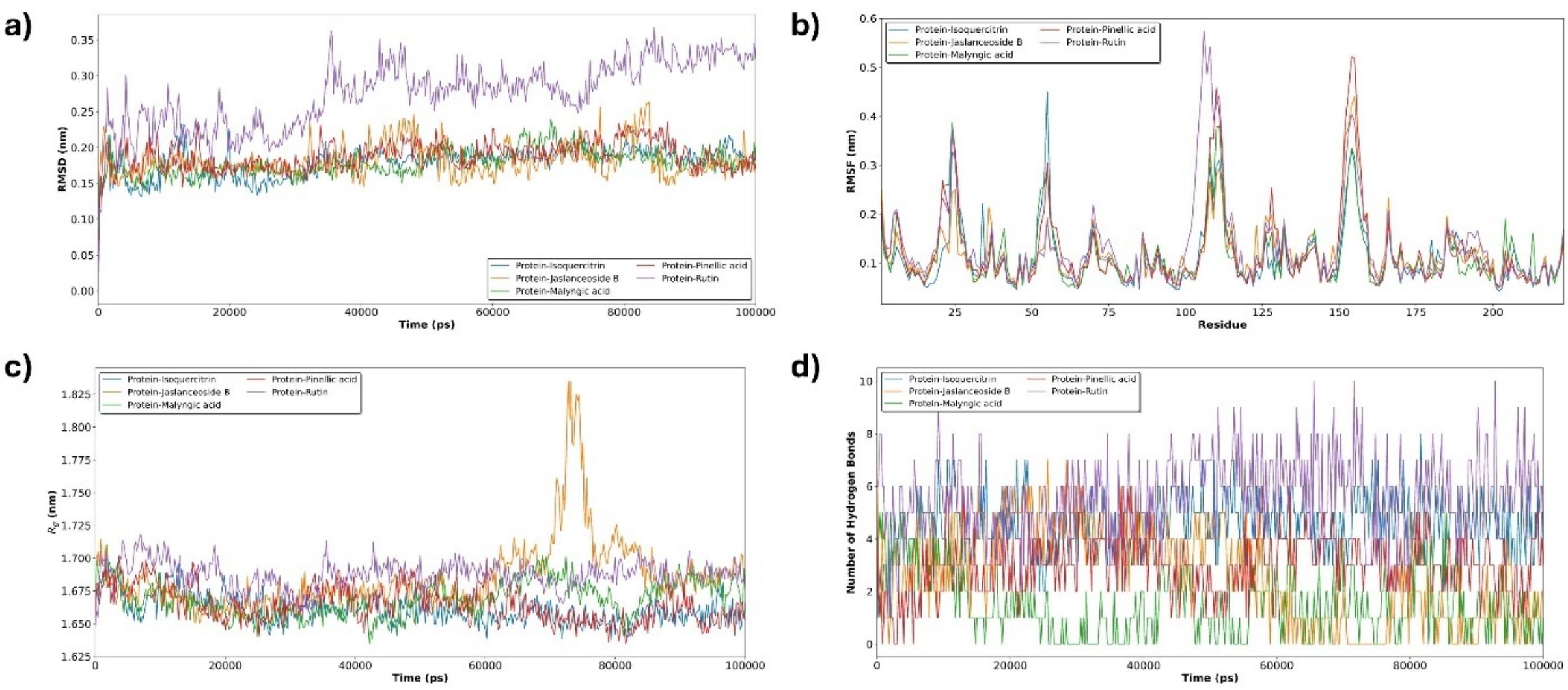

Figure S12. Plots of data obtained from the Molecular Dynamics (MD) simulation of the Endoglucanase A protein from *A. niger* with the five best docking scores. a-) RMSD b-) RMSF c-) Radius of gyration d-) H-bond.

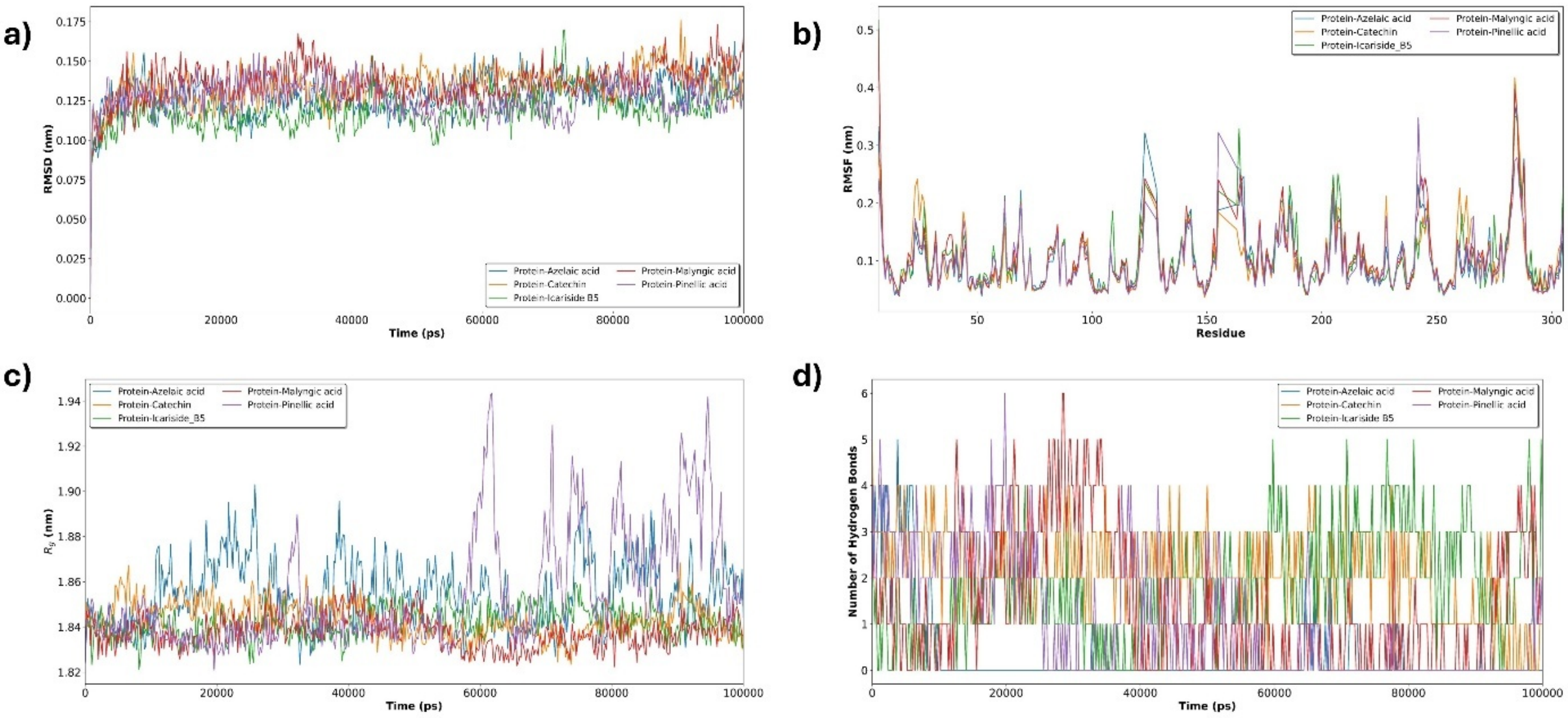

Figure S13. Plots of data obtained from the Molecular Dynamics (MD) simulation of the Sphingomyelinase C protein from *B. cereus* with the five best docking scores. a-) RMSD b-) RMSF c-) Radius of gyration d-) H-bond.

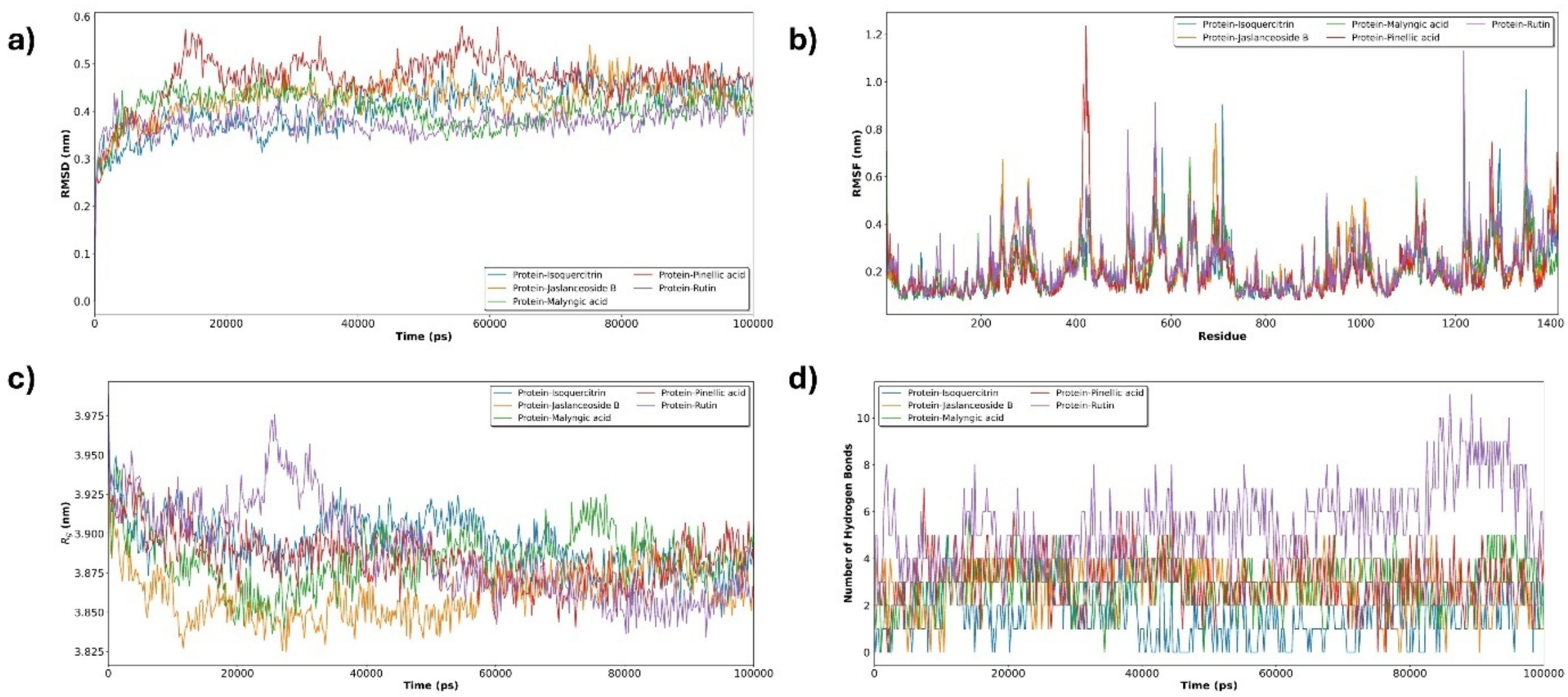

Figure S14. Plots of data obtained from the Molecular Dynamics (MD) simulation of the DNA gyrase protein from *E. coli* with the five best docking scores. a-) RMSD b-) RMSF c-) Radius of gyration d-) H-bond.

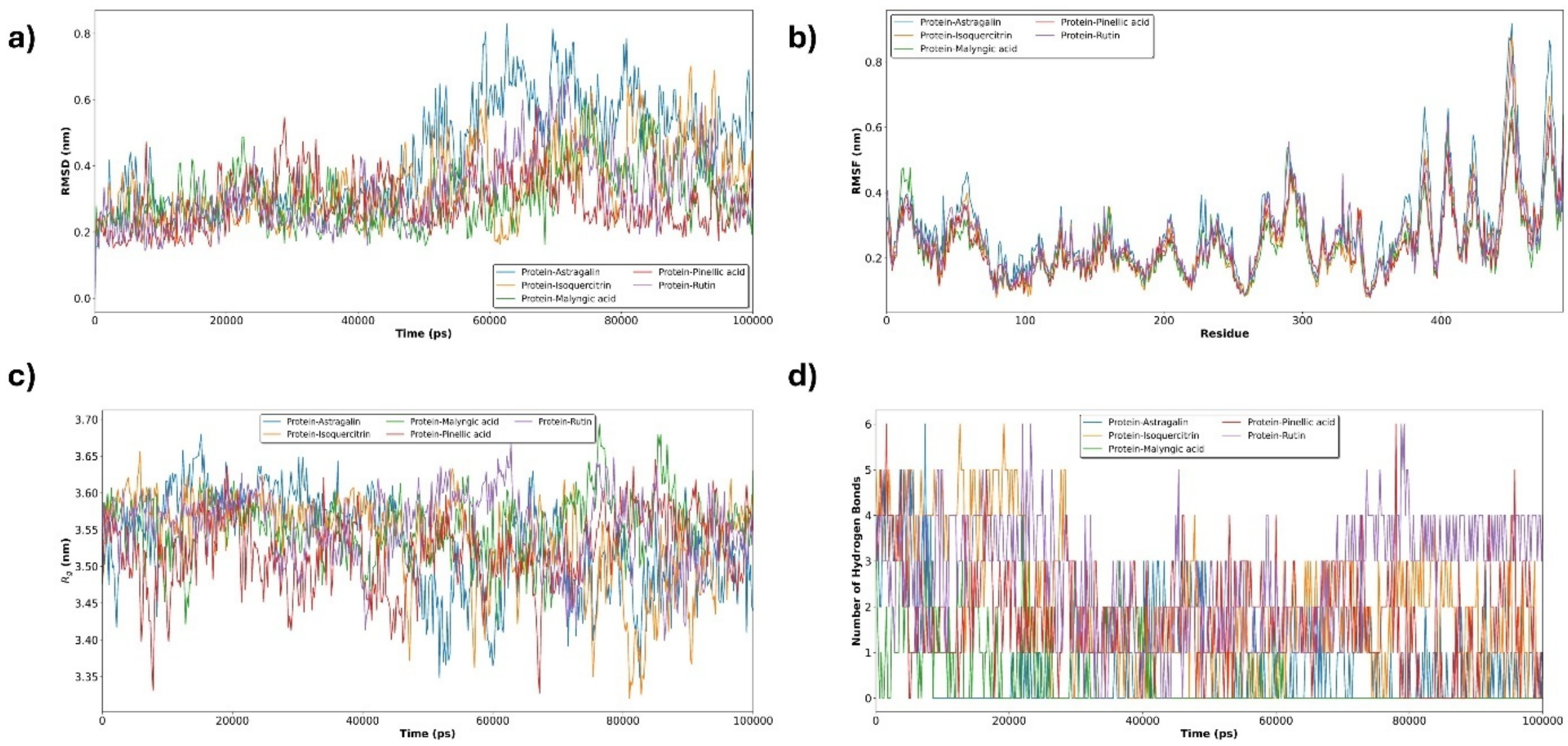

Figure S15. Plots of data obtained from the Molecular Dynamics (MD) simulation of the Listeriolysin O protein from *L. monocytogenes* with the five best docking scores. a-) RMSD b-) RMSF c-) Radius of gyration d-) H-bond.

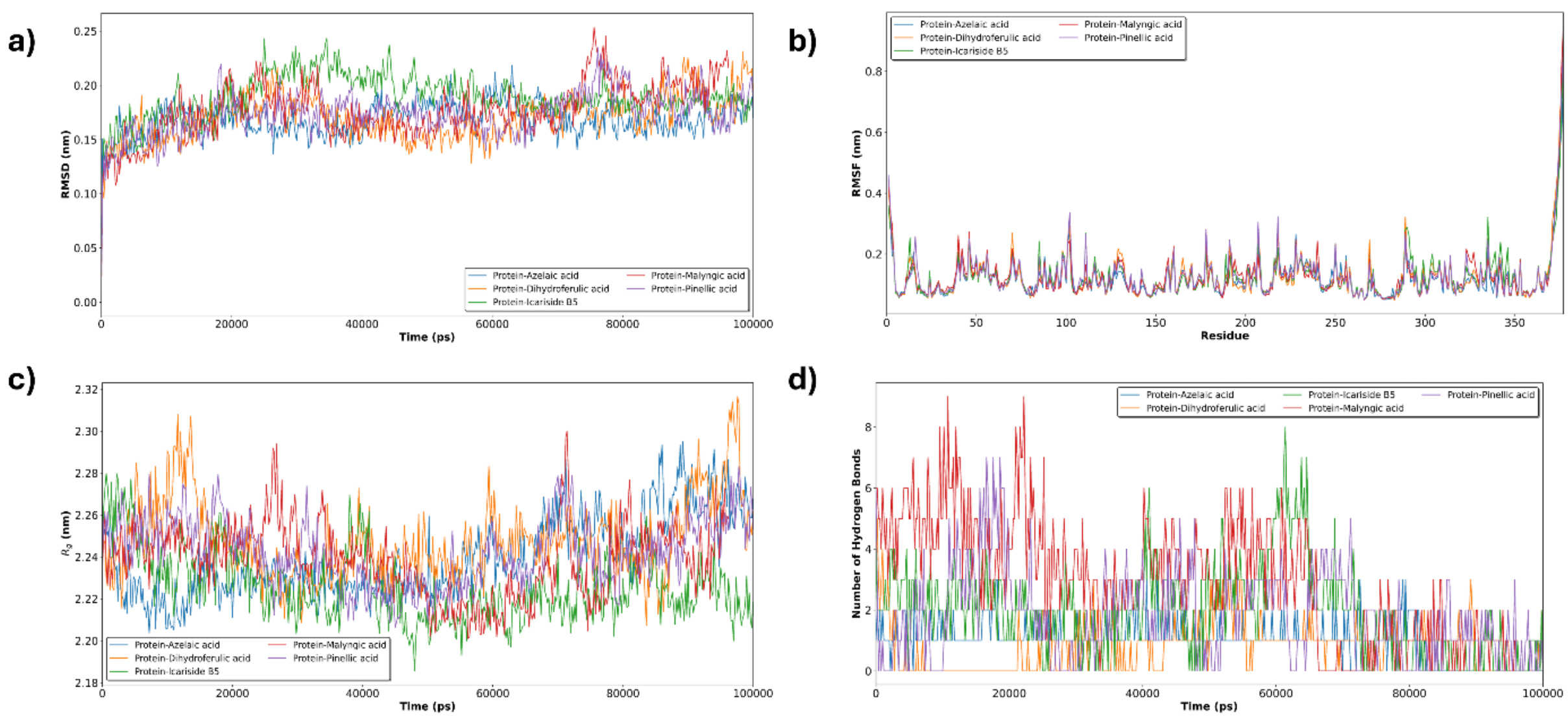

Figure S16. Plots of data obtained from the Molecular Dynamics (MD) simulation of the Arginine rhamnosyltransferase protein from *P. aeruginosa* with the five best docking scores. a-) RMSD b-) RMSF c-) Radius of gyration d-) H-bond.

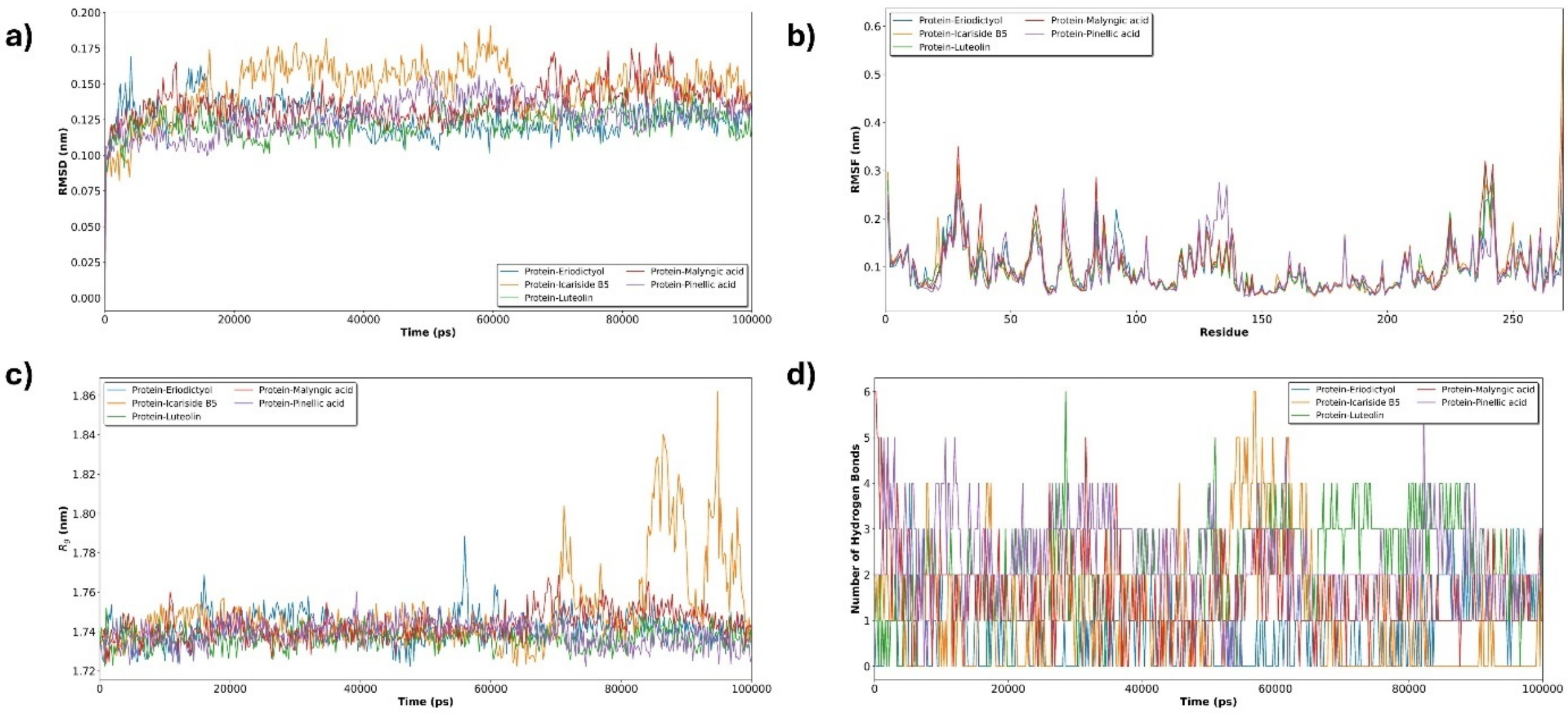

Figure S17. Plots of data obtained from the Molecular Dynamics (MD) simulation of the mono-/diacylglycerol lipase protein from *P. cyclopium* with the five best docking scores. a-) RMSD b-) RMSF c-) Radius of gyration d-) H-bond.

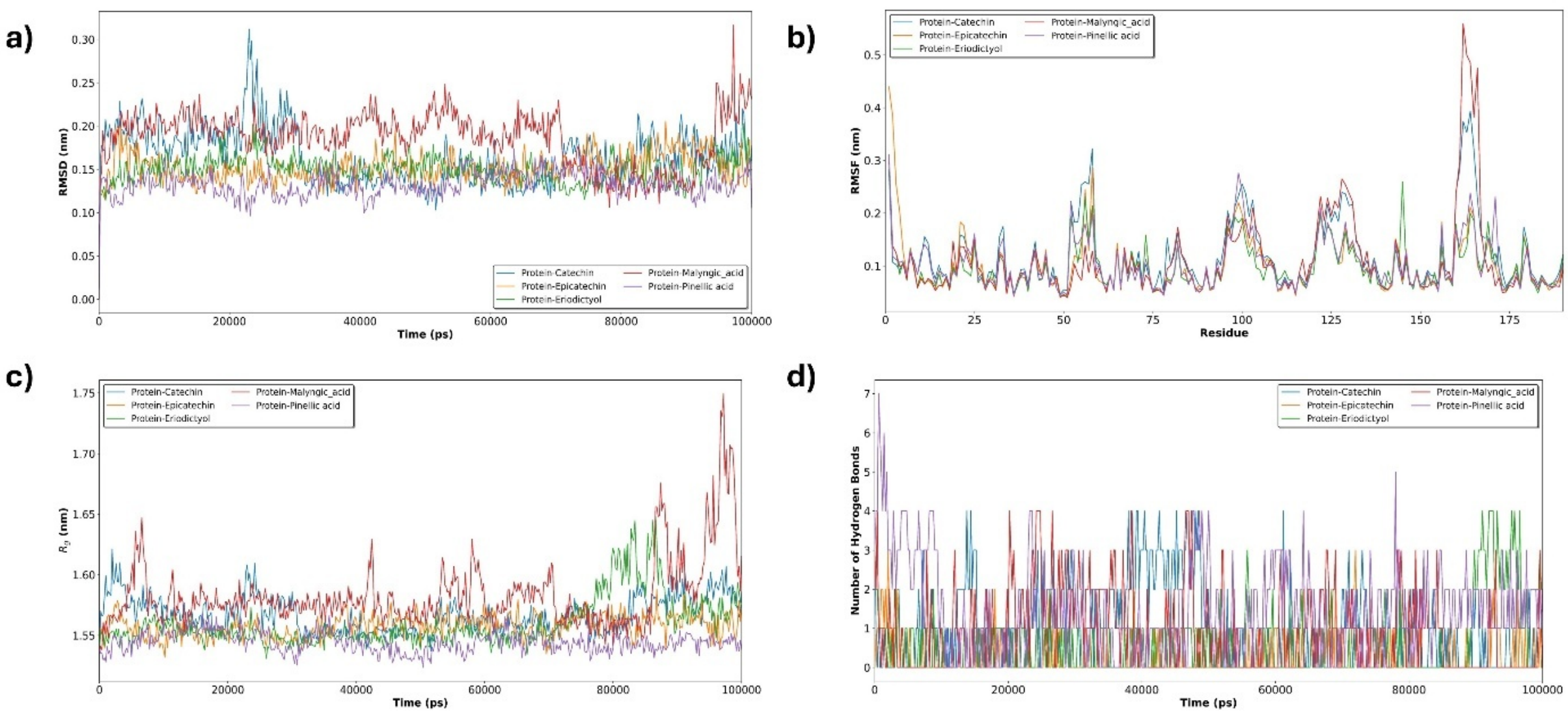

Figure S18. Plots of data obtained from the Molecular Dynamics (MD) simulation of the endo-1,4-β-xylanase C protein from *P. funiculosum* with the five best docking scores. a-) RMSD b-) RMSF c-) Radius of gyration d-) H-bond.

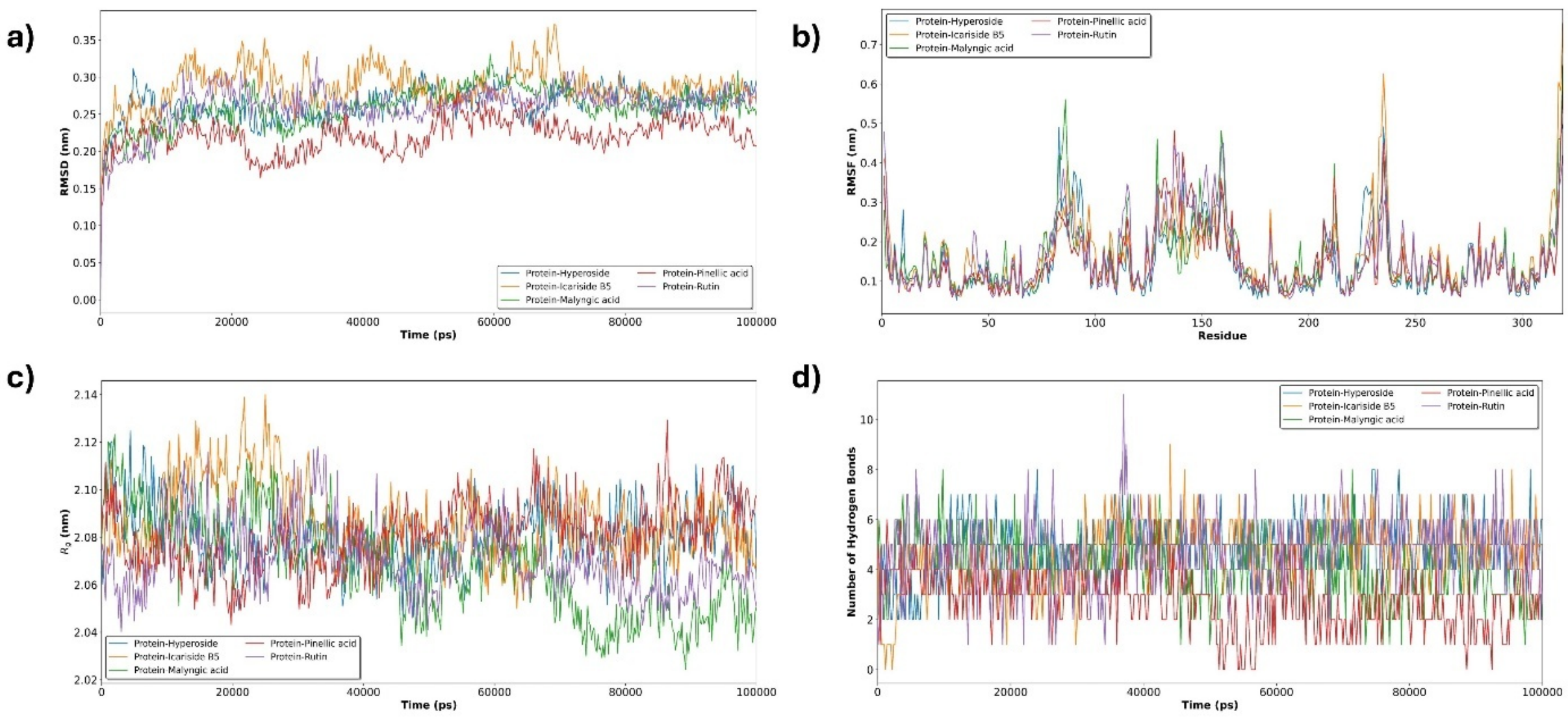

Figure S19. Plots of data obtained from the Molecular Dynamics (MD) simulation of the tyrosyl-tRNA synthetase protein from *S. aureus* with the five best docking scores. a-) RMSD b-) RMSF c-) Radius of gyration d-) H-bond.

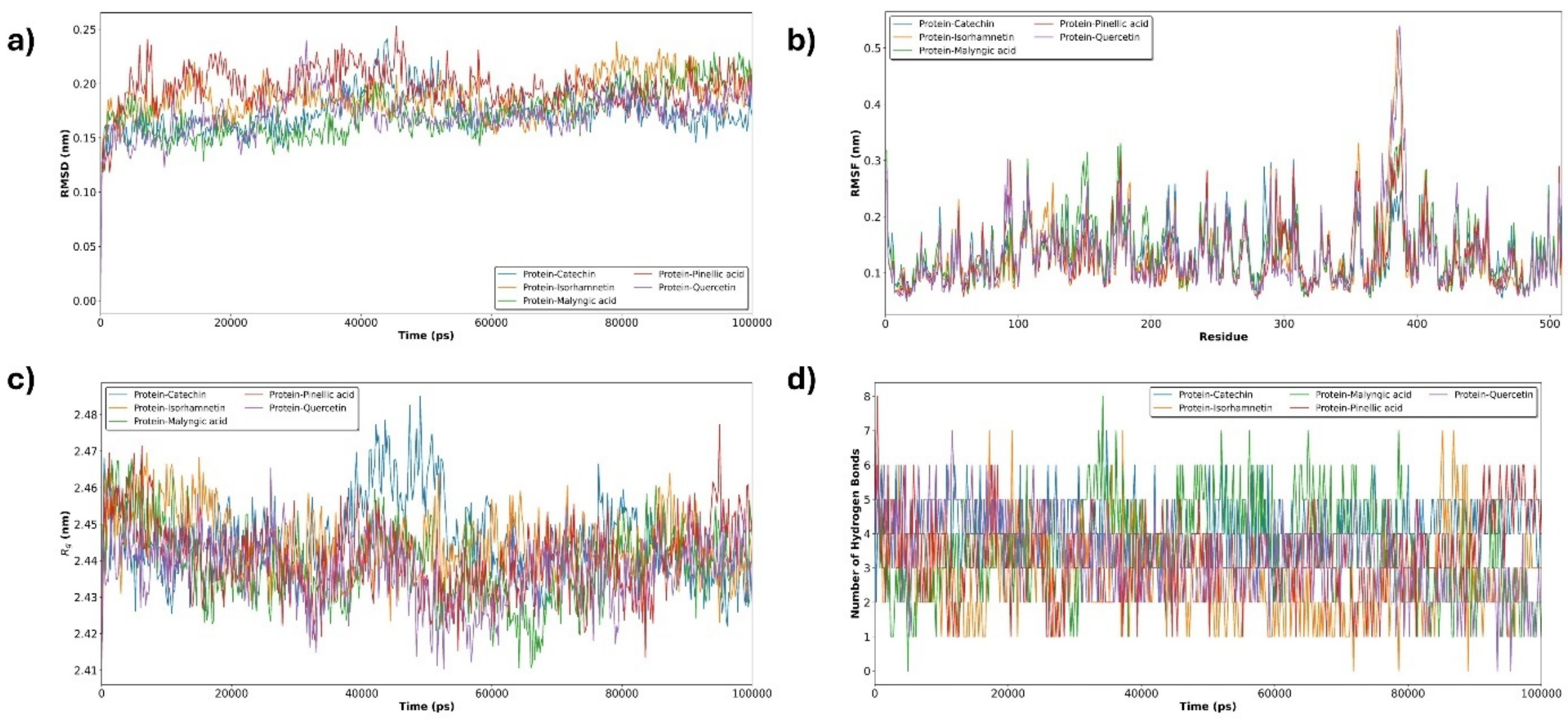

Figure S20. Plots of data obtained from the Molecular Dynamics (MD) simulation of the L-lysine oxidase protein from *T. viride* with the five best docking scores. a-) RMSD b-) RMSF c-) Radius of gyration d-) H-bond.

References

1. Y. A. Litvinenko, G. Seitimova, G. S. Burasheva and M. I. Choudhary, Chemistry of Natural Compounds, 2019, 55, 133–134.
